# Discovery of Novel 2-Substituted Aniline Pyrimidine Based Derivatives as Potent Mer/c-Met Dual Inhibitors with Improvement Bioavailability

**DOI:** 10.3390/biom15081180

**Published:** 2025-08-18

**Authors:** Jixia Yang, Daowei Huang, Ruojin Wang, Pengxin Fan, Rourou Li, Donglai Ma

**Affiliations:** 1 School of Pharmacy, Hebei University of Chinese Medicine, Shijiazhuang 050200, China; jixiayang@hebcm.edu.cn (J.Y.); 13333223653@163.com (R.W.); 18000397929@163.com (P.F.); lirou2021@outlook.com (R.L.); 2 School of Chemical and Pharmaceutical Engineering, Hebei University of Science and Technology, Shijiazhuang 050018, China

**Keywords:** Mer kinase, c-Met kinase, Mer/c-Met dual inhibitor, 2-substituted aniline pyrimidine, anti-cancer

## Abstract

This study reports the rational design and systematic evaluation of a novel series of 2-substituted aniline pyrimidine derivatives as dual Mer/c-Met inhibitors. Among the synthesized compounds, 17c demonstrated potent dual kinase inhibition, with IC_50_ values of 6.4 ± 1.8 nM (Mer) and 26.1 ± 7.7 nM (c-Met). The compound exhibited significant antiproliferative activity across multiple cancer cell lines (HepG2, MDA-MB-231, and HCT116), while showing minimal hERG channel inhibition (IC_50_ > 40 μM), indicating favorable cardiac safety. Pharmacokinetic profiling revealed high metabolic stability in human liver microsomes (t_1/2_ = 53.1 min) and moderate oral bioavailability (*F*: 45.3%), with strong plasma protein-binding affinity (>95%). Mechanistic studies further demonstrated that 17c dose-dependently suppressed HCT116 cell migration and induced apoptosis. These integrated pharmacological properties position 17c as a promising therapeutic candidate for dual Mer/c-Met drive malignancies.

## 1. Introduction

Mer kinase (monocytes epithelial tissue reproductive tissue), a key member of the TAM (Tyro3-Axl-Mer) receptor tyrosine kinase family [1,2,3], orchestrates multiple cellular processes critical to immune regulation, apoptosis resistance, and inflammatory signaling. Structurally, Mer is characterized by a conserved intracellular tyrosine kinase domain and extracellular ligand-binding region. Its expression is predominantly localized to immune cells (e.g., macrophages and dendritic cells), where it mediates efferocytosis (apoptotic cell clearance) and suppresses pro-inflammatory cytokine production, thereby maintaining immune tolerance [4]. Mechanistically, Mer activation occurs through Gas6 ligand binding, initiating downstream signaling cascades, e.g., PI3K/AKT (phosphatidylinositol 3-kinase/protein kinase B) and MAPK (mitogen-activated protein kinase), that promote cell survival, chemotaxis, and tissue remodeling [4]. Importantly, Mer dysregulation has been pathologically linked to oncogenesis: overexpression in tumor cells enhances survival under chemotherapy (via BCL-2 upregulation), facilitates metastatic dissemination (through EMT activation), and fosters immunosuppressive microenvironments (by polarizing tumor-associated macrophages) [5,6,7,8]. These multifaceted roles have established Mer as a high-value therapeutic target for precision oncology strategies targeting both tumor-intrinsic and immune evasion mechanisms.

**Figure 1:**
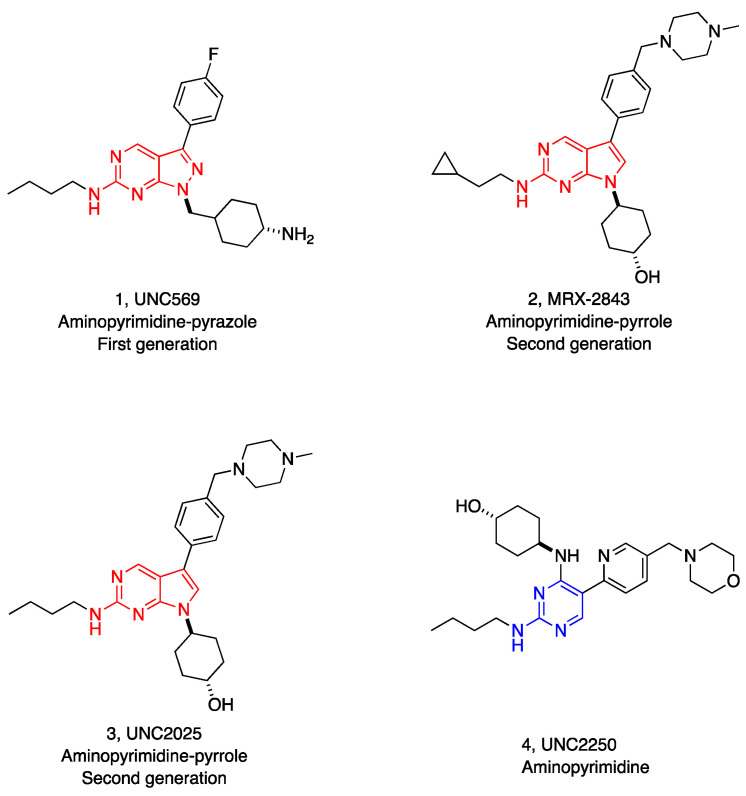
The typical Mer inhibitors.

Current Mer kinase inhibitors are structurally categorized into two major chemotypes: aminopyrimidine-pyrazole (pyrrole) and aminopyrimidine derivatives (Figure 1). The first-generation inhibitor UNC569 [9,10], a pyrazole-based compound, selectively inhibits Mer kinase (IC_50_ = 1.2 nM) and suppresses downstream ERK (extracellular signal-regulated kinase/AKT signaling. However, its clinical translation is hindered by its rapid metabolism (human microsomal t*_1/2_*< 15 min) and low oral bioavailability (F < 10%) [11]. The structural optimization of UNC569 yielded second-generation analogs. UNC2025 enhanced metabolic stability (t_1/2_ = 68 min in human hepatocytes) and maintained Mer potency (IC_50_ = 1.8 nM) [12,13]. MRX2843, a dual Mer/Flt3 (fms-like tyrosine kinase 3) inhibitor (Mer IC_50_ = 1.2 nM), showed improved CNS penetration (brain/plasma ration = 0.4) [11] and is currently in phase II trails for relapsed/refractory acute lymphoblastic leukemia (NCT04872478) [14]. Notably, the aminopyrimidine-class inhibitor UNC2250 demonstrates exceptional Mer targeting (IC_50_ = 1.7 nM) and unique activity against Mer-EGFR (epidermal growth factor receptor) fusion protein [15,16]. In xenograft models, UNC2250 induced tumor regression (~60% volume reduction vs. control) by the dual blockade of Mer-mediated survival signaling and EGFR-driven proliferation [16].

The c-Met kinase (cellular mesenchymal–epithelial transition factor), also known as the hepatocyte growth factor receptor (HGFR) [17,18], is a transmembrane receptor tyrosine kinase that plays a pivotal role in regulating critical cellular processes, including proliferation, survival, migration, and angiogenesis [19,20]. The activation of c-Met occurs through binding to its specific ligand HGF, which triggers receptor dimerization and autophosphorylation. This activation initiates downstream signaling cascades, such as the MAPK, PI3K/AKT, and STAT (signal transducer and activator of transcription) pathways. Aberrant c-Met signaling, caused by overexpression, mutations, or gene amplification, is strongly associated with tumorigenesis, promoting cancer progression, metastasis, and therapy resistance. Given its oncogenic significance, c-Met has become a promising therapeutic target. Current clinical investigations are actively exploring both small-molecule inhibitors and monoclonal antibodies targeting c-Met for the treatment of diverse malignancies.

**Figure 2:**
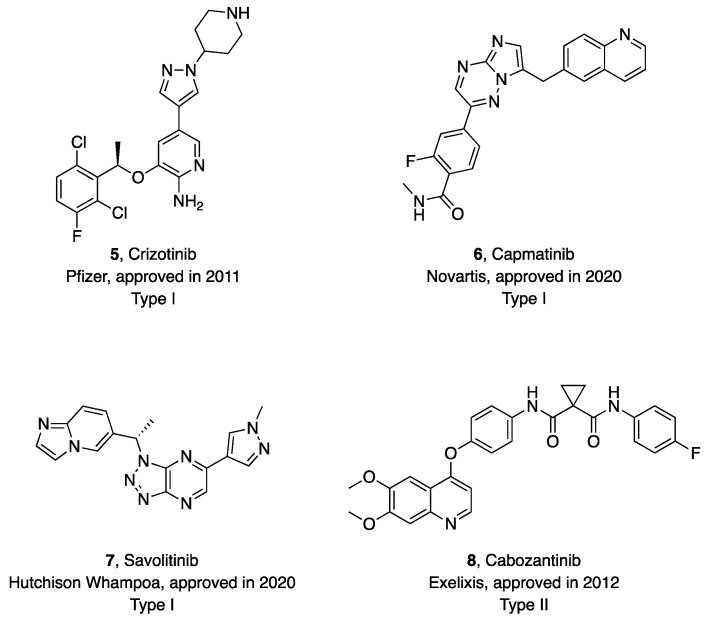
The representative c-Met inhibitors.

Multiple c-Met inhibitors have entered clinical practice or are under clinical investigation (Figure 2); these could be classified into Type I and Type II inhibitors based on their binding modes to the target kinase. Notably, crizotinib (Type I), approved by the FDA in 2011, is primarily used for ALK (anaplastic lymphoma kinase)-positive metastatic non-small cell lung cancer (NSCLC) but also exhibits c-Met inhibitory activity as part of its multi-target mechanism [21,22]. Cabozantinib (Type II), another tyrosine inhibitor targeting c-Met, VEGFR (vascular endothelial growth factor receptor), Mer, and Kit, has been approved for multiple indications: metastatic medullary thyroid cancer, as a second-line treatment of advanced renal cell carcinoma post-antiangiogenic therapy, and as a first-line therapy for advanced renal cell carcinoma [23,24]. Additionally, savolitinib (Type I), a highly selective c-Met inhibitor, is currently in Phase III clinical trials for papillary renal cell carcinoma and other c-Met-driven malignancies [25]. Capmatinib (Type I), a selective c-Met inhibitor, received FDA approval specifically for metastatic NSCLC with MET exon 14 skipping mutations [26,27].

Dual-target inhibition strategies have gained significant traction in anti-tumor drug development, as exemplified by the FDA-approved agent cabozantinib-a potent c-Met/VEGFR-2 dual kinase inhibitor. Structurally, cabozantinib’s quinoline core competitively binds to the hinge region of c-Met kinase, while its cyclopropylamide side chain enables optimal hydrophobic interaction within the VEGFR-2 active site. The bifunction design confers robust inhibitory activity against both targets, underpinning its clinical approval for advanced thyroid carcinoma, hepatocellular carcinoma (HCC), and renal cell carcinoma (RCC). Additionally, the dual-target strategy has also been effectively applied in other disease areas, such as in the treatment of neglected diseases and neurological disorders [28,29]. Consequently, the development of dual inhibitors represents a paradigm shift in oncology drug discovery, balancing enhanced therapeutic outcomes with improved safety profiles through rationally engineered polypharmacology.

Both Mer and c-Met, members of the receptor tyrosine kinase family, possess structurally homologous extracellular domains and share overlapping downstream signaling pathways. Notably, their functional convergence in regulating tumor proliferation, metastasis, and immune evasion establishes a compelling rationale for the dual targeting of Mer and c-Met kinases. To date, cabozantinib and its derivatives remain the only clinical available agent with demonstrated inhibitory activity against both targets [30]. However, cabozantinib’s clinical utility is predominantly attributed to its potent inhibition of c-Met and VEGFR-2, while the structural determinants governing its Mer kinase inhibitory activity remains poorly characterized. Furthermore, the emergence of cabozantinib resistance underscores the critical need to develop novel Mer/c-Met dual inhibitors with distinct chemical scaffolds. Importantly, the structure–activity relationship (SAR) of Mer/c-Met dual inhibitors remains underexplored, and the systematic exploration of these aspects through rational drug design is therefore essential to advance the development of next-generation dual-targeting agents.

**Figure 3:**
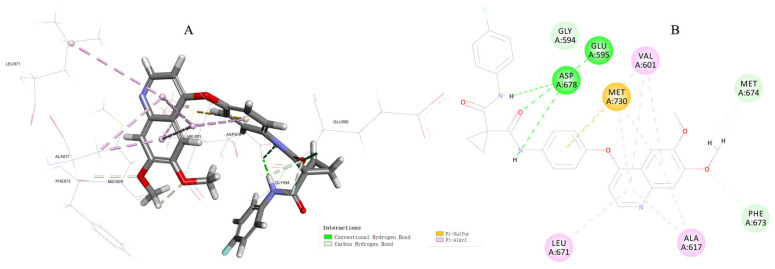
Cabozantinib binding mode in Mer kinase (PDB: 4M3Q), (**A**) 3D ligand–protein interactions; (**B**) 2D ligand interaction diagram.

**Figure 4:**
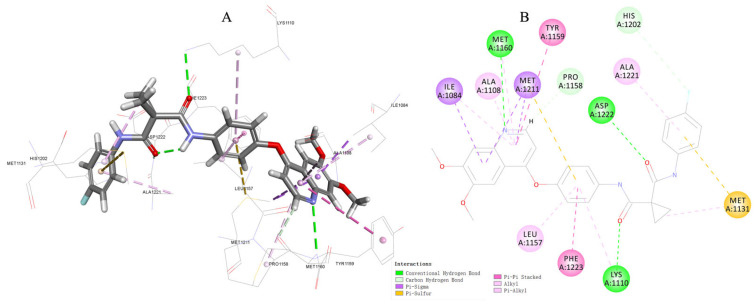
Cabozantinib binding mode in Mer kinase (PDB: 3LQ8), (**A**) 3D ligand–protein interactions; (**B**) 2D ligand interaction diagram.

**Figure 5:**
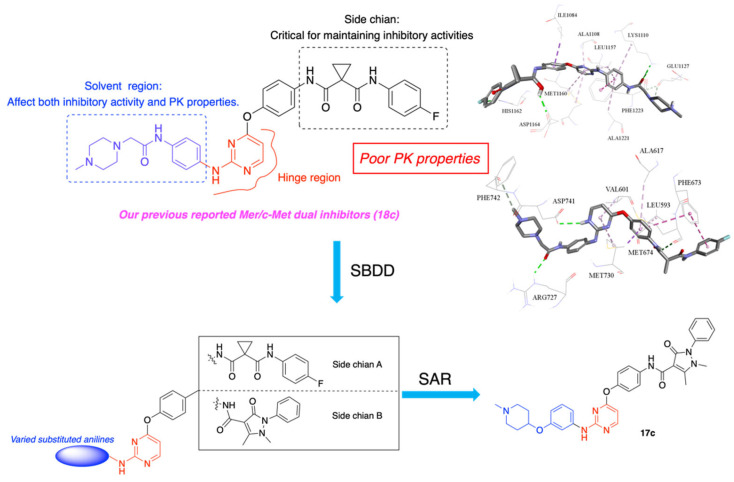
Design strategy of target compounds.

The 2-substituted aniline scaffold has been widely applied in anti-tumor drug development. For instance, the Mer inhibitor UNC2250 (Figure 1) exhibits potent Mer inhibitory activity [15,16]. Additionally, our previous study identified compound **18c** (Figure 5) as a dual Mer/c-Met dual inhibitor with potent dual-target inhibitory activity [31]. The IC_50_ values were 18.5 ± 2.3 nM and 33.6 ± 4.3 nM, respectively. Furthermore, compound **18c** also demonstrated potent antiproliferation activities against HepG2, MDA-MB-231, and HCT116 cancer cell lines and showed a low risk in hERG assays. However, pharmacokinetic analysis revealed low oral bioavailability (F: 2.84%), promoting structural optimization. To elucidate the key interactions in dual-target inhibition, we analyzed the binding modes of cabozantinib with Mer (PDB: 4M3Q) [32,33] and c-Met (PDB: 3LQ8) [34,35]. Molecular docking showed that in Mer kinase, cabozantinib’s amide group forms hydrogen bonds with the hinge region (Pro672 and Met674), while the quinoline moiety occupies a hydrophobic cavity without direct hydrogen bonding (Figure 3). In c-Met kinase, both the quinoline core and the amide group participate in hydrogen bonding with residues in the ATP-binding pocket (Figure 4). Based on these observations and the binding mode of **18c** [31], we propose modifying the 2-position of the pyrimidine ring to introduce hydrophilic groups. This strategy aims to enhance solubility and bioavailability while preserving dual-target activity.

Building upon our previous work (Figure 5), we designed and synthesized a series of 2-substituted aniline pyrimidine derivatives, with a subsequent evaluation of their biological activities. Among these, compound **17c** emerged as a lead candidate due to its superior metabolic stability in human liver microsomes and potent antiproliferative activities against the HepG2 (liver), MDA-MB-231 (breast), and HCT116 (colon) cancer cell lines. **17c** also revealed favorable pharmacokinetic properties, plasma protein binding, and a favorable safety profile in hERG assays. Mechanistic studies further showed that **17c** induced dose-dependent apoptosis in HCT116 cells and effectively inhibited HCT116 cell migration. These results position **17c** as a promising dual inhibitor of Mer and c-Met with significant development potential.

## 2. Materials and Methods

### 2.1. Chemical Part

All reagents and solvents were sourced from Titan Chemical Co., Ltd. (Shanghai, China), and Shijiazhuang Kehai Huabo Instruments Co., Ltd. (Shijiazhuang, China), respectively, and used without further purification unless specified. The reaction process was monitored by TLC analysis on silica GF254 Plates. High-resolution mass spectra (HRMS) data were acquired in ESI mode using a Water Q-Tof mass spectrometer (Waters Xevo G2-XS QTof). ^1^H- and ^13^C-NMR spectra were recorded on Bruker AM-400/500 spectrometers (Bruker Bioscience, USA) with DMSO-*d*_6_ used as solvent and tetramethylsilane (TMS) used as the internal reference, and chemical shift values were reported in ppm. The multiplicity of the signals is denoted as follows: s (singlet); d (doublet), t (triplet), q (quadruplet), qui (quintuplet), m (multiplet), dd (doublet of doublets), and dt (doublet of triplets). Coupling constants (*J*) are given in hertz (Hz). Flash chromatography was performed using silica gel (200–300 mesh) as the stationary phase, with a mobile phase consisting of a mixture of methanol (MeOH), ethyl acetate (EA), and petroleum ether (PE). The purity of all synthesized compounds was confirmed to be >95% by high-performance liquid chromatography (HPLC) using a Waters HPLC system equipped with a UV/visible detector (Waters Corporation, MA, USA), monitored at 254 nm. HPLC analysis was carried out on a 5 μm, 4.6 × 250 mm Hypersil ODS2 column, with a mobile phase consisting of a 3:7 (*v*/*v*) mixture of potassium dihydrogen phosphate solution and methanol, at a flow rate of 1.0 mL/min and an injection volume of 10 μL. All chemicals were of analytical grade and used without further purification.

#### 2.1.1. Preparation of *N*-(4-Fluorophenyl)-*N*-(4-hydroxyphenyl)cyclopropane-1,1-dicarboxamide (Intermediate **11**)

Intermediate **11** was prepared as a white solid in an 80.6% yield according to our previous work [31]. Consistent with prior methodology, the crude product required no purification and was employed directly in subsequent steps.

#### 2.1.2. Preparation of *N*-(4-((2-Chloropyrimidin-4-yl)oxy)phenyl)-*N*-(4-fluorophenyl)cyclopropane-1,1-dicarboxamide (Intermediate **12**)

Intermediate **12** was prepared as a white solid in a 75.7% yield according to our previous work [31].

#### 2.1.3. General Procedure for Preparation of Target Compounds **13a**–**13n**

Target compounds **13a**–**13n** were prepared according to our previous work [31].

*N*-(4-fluorophenyl)-*N*-(4-((2-((4-((1-methylpiperidin-4-yl)oxy)phenyl)amino)pyrimidin-4-yl)oxy)phenyl)cyclopropane-1,1-dicarboxamide (compound **13a**): White solid, yield: 23.27%. ^1^H NMR (500 MHz, DMSO-*d*_6_) *δ* 10.18 (s, 1H), 10.11 (s, 1H), 9.41 (s, 1H), 8.30 (d, *J* = 5.6 Hz, 1H), 7.70 (d, *J* = 8.9 Hz, 2H), 7.66 (dd, *J* = 9.1, 5.1 Hz, 2H), 7.43 (d, *J* = 7.5 Hz, 2H), 7.19–7.14 (m, 4H), 6.80 (d, *J* = 8.7 Hz, 2H), 6.35 (d, *J* = 5.6 Hz, 1H), 4.44 (s, 1H), 3.16–2.87 (m, 4H), 2.63 (s, 3H), 2.10–2.01 (m, 2H), 1.85 (s, 2H), 1.51 (d, *J* = 4.6 Hz, 4H). ^13^C NMR (126 MHz, DMSO-*d*_6_) *δ* 170.06, 168.73 (d, *J* = 4.3 Hz, 1C), 160.19, 159.70, 157.79, 151.65, 148.59, 136.63, 135.66, 134.42, 122.86 (d, *J* = 7.7 Hz, 1C), 122.43 (d, *J* = 10.0 Hz, 1C), 120.91,116.61, 115.62, 115.44, 98.07, 78.48, 50.54, 43.37, 32.01, 28.45, 15.91. HRMS: *m*/*z* C_33_H_33_FN_6_O_4_ [M + H]^+^ 597.2547, Found 597.2628. Purity: >95% (HPLC).

*N*-(4-fluorophenyl)-*N*-(4-((2-((3-((2-methoxyethyl)amino)phenyl)amino)pyrimidin-4-yl)oxy)phenyl)cyclopropane-1,1-dicarboxamide (compound **13b**): White solid, yield: 20.08%. ^1^H NMR (500 MHz, DMSO-*d*_6_) *δ* 10.15 (s, 1H), 10.08 (s, 1H), 9.27 (s, 1H), 8.31 (d, *J* = 5.6 Hz, 1H), 7.71 (d, *J* = 9.0 Hz, 2H), 7.66 (dd, *J* = 9.0, 5.1 Hz, 2H), 7.21–7.14 (m, 4H), 6.86–6.79 (m, 3H), 6.34 (d, *J* = 5.6 Hz, 1H), 6.16 (d, *J* = 7.3 Hz, 1H), 5.21 (s, 1H), 3.44 (t, *J* = 5.9 Hz, 2H), 3.26 (s, 3H), 3.06 (t, *J* = 5.8 Hz, 2H), 1.49 (s, 4H). ^13^C NMR (126 MHz, DMSO-*d*_6_) *δ* 169.90, 168.67, 160.37, 160.22, 159.70, 157.79, 148.56, 141.27, 136.65, 135.67 (d, *J* = 2.0 Hz, 1C), 129.15, 122.82 (d, *J* = 7.7 Hz, 1C), 122.27, 115.61, 115.43, 107.93, 106.71, 103.23, 98.27, 70.93, 58.45, 42.93, 31.95, 15.86. HRMS: *m*/*z* C_30_H_29_FN_6_O_4_ [M + H]^+^ 557.2234, Found 557.2312. Purity: >95% (HPLC).

*N*-(4-((2-((1*H*-pyrazol-4-yl)amino)pyrimidin-4-yl)oxy)phenyl)-*N*-(4-fluorophenyl)cyclopropane-1,1-dicarboxamide (compound **13c**): White solid, yield: 31.52%. ^1^H NMR (500 MHz, DMSO-*d*_6_) *δ* 12.31 (s, 1H), 10.17 (s, 1H), 10.08 (s, 1H), 9.65–9.18 (m, 1H), 8.29 (s, 1H), 7.72 (s, 2H), 7.65 (dd, *J* = 8.7, 5.0 Hz, 3H), 7.30–7.11 (m, 5H), 6.30 (d, *J* = 5.5 Hz, 1H), 1.50 (s, 4H). ^13^C NMR (126 MHz, DMSO-*d*_6_) *δ* 170.46, 168.68 (d, *J* = 11.7 Hz, 1C), 160.42, 159.84, 159.71, 157.80, 148.56, 136.67, 135.63, 130.12, 122.87 (d, *J* = 6.0 Hz, 1C), 122.40, 122.13, 115.62, 115.44, 98.04, 31.89, 15.97. HRMS: *m*/*z* C_24_H_20_FN_7_O_3_ [M + H]^+^ 474.1612, Found 474.1686. Purity: >95% (HPLC).

*N*-(4-((2-((3,5-difluorophenyl)amino)pyrimidin-4-yl)oxy)phenyl)-*N*-(4-fluorophenyl)cyclopropane-1,1-dicarboxamide (compound **13d**): White solid, yield: 19.40%. ^1^H NMR (400 MHz, DMSO-*d*_6_) *δ* 10.23 (s, 1H), 10.07 (s, 1H), 10.01 (s, 1H), 8.47 (d, *J* = 5.6 Hz, 1H), 7.78 (d, *J* = 8.8 Hz, 2H), 7.69 (dd, *J* = 8.7, 5.1 Hz, 2H), 7.34 (d, *J* = 9.4 Hz, 2H), 7.28–7.17 (m, 4H), 6.67 (t, *J* = 9.1 Hz, 1H), 6.59 (d, *J* = 5.6 Hz, 1H), 1.53 (d, *J* = 4.1 Hz, 4H). ^13^C NMR (101 MHz, DMSO-*d*_6_) *δ* 170.09, 168.56, 160.40, 159.61, 157.59, 148.25, 143.31, 137.04, 135.59 (d, *J* = 2.6 Hz, 1C), 122.93 (d, *J* = 7.8 Hz, 1C), 122.29, 122.05, 115.62, 115.40, 101.74, 101.44, 99.97, 31.88, 15.93. HRMS: *m*/*z* C_27_H_20_F_3_N_5_O_3_ [M + H]^+^ 520.1518, Found 520.1594. Purity: >95% (HPLC).

*N*-(4-((2-((3-(4-acetylpiperazin-1-yl)phenyl)amino)pyrimidin-4-yl)oxy)phenyl)-*N*-(4-fluorophenyl)cyclopropane-1,1-dicarboxamide (compound **13e**): White solid, yield: 23.07%. ^1^H NMR (400 MHz, DMSO-*d*_6_) δ 10.15 (s, 1H), 10.07 (s, 1H), 9.38 (s, 1H), 8.34 (d, *J* = 5.6 Hz, 1H), 7.70 (d, *J* = 9.0 Hz, 2H), 7.64 (dd, *J* = 9.1, 5.1 Hz, 2H), 7.20–7.13 (m, 5H), 7.08 (d, *J* = 8.1 Hz, 1H), 6.97 (t, *J* = 8.1 Hz, 1H), 6.47 (dd, *J* = 8.1, 1.7 Hz, 1H), 6.39 (d, *J* = 5.6 Hz, 1H), 3.56–3.47 (dd, *J* = 10.5, 7.0 Hz, 4H), 2.98 (t, *J* = 4.7 Hz, 2H), 2.93 (t, *J* = 4.9 Hz, 2H), 2.03 (s, 3H), 1.47 (s, 4H). ^13^C NMR (101 MHz, DMSO-*d*_6_) δ 169.82, 168.71 (d, *J* = 4.3 Hz, 1C), 168.60, 160.30 (d, *J* = 7.60 Hz, 1C), 159.95, 157.56, 151.52, 148.56, 141.30, 136.59, 135.66 (d, *J* = 2.5 Hz, 1C), 129.26, 122.85 (d, *J* = 7.9 Hz, 1C), 122.10, 115.62, 115.40, 111.05, 110.13, 107.05, 98.72, 49.23, 45.87, 31.97, 21.64, 15.85. HRMS: *m*/*z* C_33_H_32_FN_7_O_4_ [M + H]^+^ 610.2500, Found 610.2576. Purity: >95% (HPLC).

*N*-(4-fluorophenyl)-*N*-(4-((2-((3-(piperidin-3-ylcarbamoyl)phenyl)amino)pyrimidin-4-yl)oxy)phenyl)cyclopropane-1,1-dicarboxamide (compound **13f**): White solid, yield: 31.00%. ^1^H NMR (500 MHz, DMSO-*d*_6_) δ 10.11 (s, 1H), 10.07 (s, 1H), 8.23 (d, *J* = 5.6 Hz, 1H), 8.10 (d, *J* = 8.0 Hz, 1H), 7.67 (d, *J* = 8.9 Hz, 2H), 7.64 (dd, *J* = 8.9, 5.1 Hz, 2H), 7.19–7.12 (m, 4H), 7.07 (t, *J* = 7.8 Hz, 1H), 7.02 (s, 1H), 6.94 (d, *J* = 7.7 Hz, 1H), 6.69 (dd, *J* = 7.9, 1.5 Hz, 1H), 6.07 (d, *J* = 5.4 Hz, 1H), 5.27 (s, 1H), 4.59–4.20 (m, 2H), 3.83–3.74 (m, 1H), 2.88–2.76 (m, 2H), 1.93–1.85 (m, 1H), 1.75–1.68 (m, 1H), 1.64–1.54 (m, 1H), 1.46 (s, 4H), 1.44–1.34 (m, 2H). ^13^C NMR (126 MHz, DMSO-*d*_6_) δ 169.81, 168.62 (d, *J* = 11.21 Hz, 1C), 167.20, 161.64, 160.43, 159.70, 157.79, 148.95, 148.31, 136.40, 136.11, 135.63 (d, *J* = 1.61 Hz, 1C), 129.02, 122.88 (d, *J* = 7.70 Hz, 1C), 121.96, 116.86, 115.58, 115.41, 115.05, 113.40, 95.84, 48.53, 46.15, 43.81, 31.91, 30.76, 23.97, 15.91. HRMS: *m*/*z* C_24_H_20_FN_7_O_3_ [M + H]^+^ 610.2500, Found 610.2576. Purity: >95% (HPLC).

*N*-(4-fluorophenyl)-*N*-(4-((2-((4-(piperidin-3-ylcarbamoyl)phenyl)amino)pyrimidin-4-yl)oxy)phenyl)cyclopropane-1,1-dicarboxamide (compound **13g**): White solid, yield: 28.15%. ^1^H NMR (500 MHz, DMSO-*d*_6_) δ 10.10 (s, 1H), 10.08 (s, 1H), 8.22 (d, *J* = 5.5 Hz, 1H), 7.81 (d, *J* = 7.9 Hz, 1H), 7.69–7.61 (m, 4H), 7.58 (d, *J* = 8.4 Hz, 2H), 7.15 (t, *J* = 8.2 Hz, 4H), 6.54 (d, *J* = 8.4 Hz, 2H), 6.06 (d, *J* = 4.9 Hz, 1H), 5.64 (s, 1H), 4.62–4.15 (m, 2H), 3.83–3.72 (m, 1H), 2.82 (t, *J* = 11.5 Hz, 2H), 1.88 (d, *J* = 9.6 Hz, 1H), 1.71 (d, *J* = 11.6 Hz, 1H), 1.61–1.51 (m, 1H), 1.46 (s, 4H), 1.44–1.33 (m, 2H). ^13^C NMR (126 MHz, DMSO-*d*_6_) δ 169.81, 168.62 (d, *J* = 9.89 Hz, 1C), 166.27, 161.62, 160.39, 159.70, 157.79, 152.00, 148.32, 136.37, 135.62 (d, *J* = 1.39 Hz, 1C), 129.33, 122.88 (d, *J* = 7.70 Hz, 1C), 122.04, 121.94, 115.59, 115.41, 112.95, 95.78, 48.76, 45.99, 43.83, 31.88, 30.93, 24.04, 15.92. HRMS: *m*/*z* C_24_H_20_FN_7_O_3_ [M + H]^+^ 610.2500, Found 610.2567. Purity: >95% (HPLC).

*N*-(4-fluorophenyl)-*N*-(4-((2-((4-(4-methylpiperazine-1-carbonyl)phenyl)amino)pyrimidin-4-yl)oxy)phenyl)cyclopropane-1,1-dicarboxamide (compound **13h**): White solid, yield: 18.28%. ^1^H NMR (400 MHz, DMSO-*d*_6_) δ 10.18 (s, 1H), 10.09 (s, 1H), 9.80 (s, 1H), 8.38 (d, *J* = 5.6 Hz, 1H), 7.71 (d, *J* = 9.0 Hz, 2H), 7.65 (dd, *J* = 9.1, 5.1 Hz, 2H), 7.58 (d, *J* = 8.5 Hz, 2H), 7.21 (d, *J* = 9.0 Hz, 2H), 7.19–7.13 (m, 4H), 6.48 (d, *J* = 5.6 Hz, 1H), 3.46 (s, 4H), 2.32 (s, 4H), 2.21 (s, 3H), 1.50 (s, 4H). ^13^C NMR (101 MHz, DMSO-*d*_6_) δ 170.11, 169.51, 168.71, 160.41, 159.90, 157.56, 148.52, 141.96, 136.78, 135.62 (d, *J* = 2.5 Hz, 1C), 128.19, 122.89 (d, *J* = 7.9 Hz, 1C), 122.51, 122.33, 118.39, 115.62, 115.40, 99.15, 54.92, 45.97, 31.81, 31.62, 16.07. HRMS: *m*/*z* C_33_H_32_FN_7_O_4_ [M + H]^+^ 610.2500, Found 610.2568. Purity: >95% (HPLC).

*N*-(4-((2-((4-(2-((2-(diethylamino)ethyl)amino)acetamido)phenyl)amino)pyrimidin-4-yl)oxy)phenyl)-*N*-(4-fluorophenyl)cyclopropane-1,1-dicarboxamide (compound **13i**): White solid, yield: 16.50%. ^1^H NMR (400 MHz, DMSO-*d*_6_) δ 10.15 (s, 1H), 10.09 (s, 1H), 9.86 (s, 1H), 9.48 (s, 1H), 8.32 (d, *J* = 5.6 Hz, 1H), 7.71 (d, *J* = 8.9 Hz, 2H), 7.65 (dd, *J* = 9.0, 5.1 Hz, 2H), 7.51 (d, *J* = 8.7 Hz, 2H), 7.40 (d, *J* = 8.8 Hz, 2H), 7.20–7.13 (m, 4H), 6.36 (d, *J* = 5.6 Hz, 1H), 3.36 (s, 3H), 2.90 (s, 2H), 2.75 (s, 6H), 1.51 (d, *J* = 5.2 Hz, 4H), 1.06 (t, *J* = 7.1 Hz, 6H). ^13^C NMR (101 MHz, DMSO-*d*_6_) δ 170.02, 169.51, 168.74 (d, *J* = 2.1 Hz, 1C), 160.17, 159.94, 157.55, 148.51, 136.72, 136.35, 135.64 (d, *J* = 2.6 Hz, 1C), 133.07, 122.86 (d, *J* = 7.8 Hz, 1C), 122.25 (d, *J* = 12.7 Hz, 1C), 120.10, 119.64, 115.62, 115.40, 98.40, 52.32, 51.46, 46.90, 45.95, 31.92, 15.99, 10.73. HRMS: *m*/*z* C_35_H_39_FN_8_O_4_ [M + H]^+^ 655.3078, Found 655.3160. Purity: >95% (HPLC).

*N*-(4-((2-((4-(2-((2-(dimethylamino)ethyl)amino)acetamido)phenyl)amino)pyrimidin-4-yl)oxy)phenyl)-*N*-(4-fluorophenyl)cyclopropane-1,1-dicarboxamide (compound **13j**): White solid, yield: 30.11%. ^1^H NMR (400 MHz, DMSO-*d*_6_) δ 10.16 (s, 1H), 10.12 (s, 1H), 9.91 (s, 1H), 9.48 (s, 1H), 8.32 (s, 1H), 7.71 (s, 2H), 7.65 (s, 2H), 7.49 (s, 2H), 7.42 (s, 2H), 7.18 (s, 4H), 6.36 (s, 1H), 3.32 (s, 3H), 2.71 (s, 2H), 2.55 (s, 2H), 2.32 (s, 6H), 1.51 (s, 4H). ^13^C NMR (101 MHz, DMSO-*d*_6_) δ 170.03, 169.76, 168.76 (d, *J* = 3.0 Hz, 1C), 160.18, 157.57, 148.54, 136.70, 136.30, 135.61 (d, *J* = 2.6 Hz, 1C) 133.12, 122.89 (d, *J* = 7.9 Hz, 1C), 122.32, 122.19, 120.01, 119.65, 115.62, 115.40, 98.39, 58.06, 52.53, 46.03, 44.87, 31.88, 16.02. HRMS: *m*/*z* C_33_H_35_FN_8_O_4_ [M + H]^+^ 627.2765, Found 627.2841. Purity: >95% (HPLC).

*N*-(4-fluorophenyl)-*N*-(4-((2-((4-(2-(4-(2-methoxyethyl)piperazin-1-yl)acetamido)phenyl)amino)pyrimidin-4-yl)oxy)phenyl)cyclopropane-1,1-dicarboxamide (compound **13k**): White solid, yield: 33.25%. ^1^H NMR (400 MHz, DMSO-*d*_6_) δ 10.14 (s, 1H), 10.09 (s, 1H), 9.48 (s, 1H), 9.46 (s, 1H), 8.32 (d, *J* = 5.6 Hz, 1H), 7.71 (d, *J* = 8.8 Hz, 2H), 7.64 (dd, *J* = 8.9, 5.1 Hz, 2H), 7.47 (d, *J* = 8.6 Hz, 2H), 7.37 (d, *J* = 8.8 Hz, 2H), 7.16 (q, *J* = 8.6 Hz, 4H), 6.36 (d, *J* = 5.6 Hz, 1H), 3.42 (t, *J* = 5.8 Hz, 2H), 3.22 (s, 3H), 3.04 (s, 2H), 2.50 (s, 2H), 2.47 (s, 8H), 1.51 (d, *J* = 8.2 Hz, 4H). ^13^C NMR (101 MHz, DMSO-*d*_6_) δ 170.05, 168.72, 168.18, 160.15, 157.56, 148.54, 136.71, 136.39, 135.63 (d, *J* = 2.5 Hz, 1C), 132.92, 122.87 (d, *J* = 7.9 Hz, 1C), 122.35, 122.17, 120.33, 119.54, 115.62, 115.40, 98.36, 70.34, 62.22, 58.46, 57.46, 53.42, 53.27, 31.89, 16.01. HRMS: *m*/*z* C_36_H_39_FN_8_O_5_ [M + H]^+^ 683.3027, Found 683.3107. Purity: >95% (HPLC).

#### 2.1.4. Preparation of *N*-(4-Hydroxyphenyl)-1,5-dimethyl-3-oxo-2-phenyl-2,3-dihydro-1*H*-pyrazole-4-carboxamide (Intermediate **15**)

A mixture of 4-antipyrine carboxylic acid (**14**, 2.0 g, 8.61 mmol) and 4-aminophenol (**10**, 1.13 g, 10.33 mmol) in DMF (20 mL) was treated with HBTU (3.92 g, 10.33 mmol) and TEA (2.61 g, 25.84 mmol). After stirring at ambient temperature for 8 h (reaction monitored by TLC), the mixture was quenched by pouring into ice water (200 mL). The resulting precipitate was collected by filtration, washed thoroughly, and vacuum-dried to afford intermediate **15** as a white solid (1.75 g, 63.0%). This product was used directly in subsequent without purification.

#### 2.1.5. Preparation of *N*-(4-((2-Chloropyrimidin-4-yl)oxy)phenyl)-1,5-dimethyl-3-oxo-2-phenyl-2,3-dihydro-1*H*-pyrazole-4-carboxamide (Intermediate **16**)

A mixture of intermediate **15** (1.75 g, 5.41 mmol) and 2,4-dichloropyrimidine (0.81 g, 5.41 mmol) in DMF (15 mL) was treated with K_2_CO_3_ (0.82 g, 5.95 mmol). The mixture was stirred at 80 °C for 4.5 h. The reaction solution was poured into ice water (100 mL), and the precipitate was filtered off, washed, and dried in a vacuum to yield intermediate **16** as a white solid (1.85 g, 78.0%), which can be used directly without any further purification.

#### 2.1.6. General Procedure for Preparation of Target Compounds **17a**–**17h**

Intermediate **16** (1.2 mmol) and substituted aniline (1.0 mmol) were dissolved in DMF (8 mL), followed by the addition of PTSA (4.00 mmol). The reaction mixture was stirred at 90 °C for 4 h under N_2_ atmosphere. After cooling to ambient temperature, the solution was quenched with ice water (100 mL). The precipitate solid was isolated by filtration, washed, and vacuum-dried. Crude product purification via silica gel chromatography (gradient elution: DCM/MeOH 100:1–30:1) afforded compounds **17a**–**17h**.

1,5-dimethyl-*N*-(4-((2-((4-((1-methylpiperidin-4-yl)oxy)phenyl)amino)pyrimidin-4-yl)oxy)phenyl)-3-oxo-2-phenyl-2,3-dihydro-1*H*-pyrazole-4-carboxamide (compound **17a**): White solid, yield: 15.05%. ^1^H NMR (500 MHz, DMSO-*d*_6_) δ 10.82 (s, 1H), 9.41 (s, 1H), 8.29 (d, *J* = 5.6 Hz, 1H), 7.68 (d, *J* = 8.8 Hz, 2H), 7.60 (t, *J* = 7.7 Hz, 2H), 7.53 (d, *J* = 7.3 Hz, 1H), 7.45 (d, *J* = 7.5 Hz, 2H), 7.40–7.34 (m, 2H), 7.18 (d, *J* = 8.8 Hz, 2H), 6.71 (d, *J* = 7.7 Hz, 2H), 6.38 (d, *J* = 5.6 Hz, 1H), 4.25 (s, 1H), 3.38 (s, 3H), 2.77 (s, 2H), 2.74 (s, 3H), 2.45 (s, 2H), 2.31 (d, *J* = 13.7 Hz, 3H), 1.90 (s, 2H), 1.68 (s, 2H).^13^C NMR (126 MHz, DMSO-*d*_6_) δ 170.12, 163.52, 161.67, 160.36, 160.15, 154.26, 151.89, 148.17, 136.67, 134.09, 133.49, 129.97, 129.33, 127.59, 123.09, 120.95, 120.76, 116.33, 97.94, 97.60, 44.97, 33.83, 21.25, 11.98. HRMS: *m*/*z* C_34_H_36_N_7_O_4_ [M + H]^+^ 606.2751, Found 606.2832. Purity: >95% (HPLC).

*N*-(4-((2-((3-((2-methoxyethyl)amino)phenyl)amino)pyrimidin-4-yl)oxy)phenyl)-1,5-dimethyl-3-oxo-2-phenyl-2,3-dihydro-1*H*-pyrazole-4-carboxamide (compound **17b**): White solid, yield: 5.80%. ^1^H NMR (500 MHz, DMSO-*d*_6_) δ 10.82 (s, 1H), 9.28 (s, 1H), 8.31 (d, *J* = 5.4 Hz, 1H), 7.69 (d, *J* = 8.5 Hz, 2H), 7.60 (t, *J* = 7.4 Hz, 3H), 7.52 (t, *J* = 7.2 Hz, 1H), 7.45 (d, *J* = 7.4 Hz, 2H), 7.19 (d, *J* = 8.6 Hz, 2H), 6.87–6.77 (m, 3H), 6.36 (d, *J* = 5.4 Hz, 1H), 6.16 (d, *J* = 6.8 Hz, 1H), 3.42 (t, *J* = 5.5 Hz, 2H), 3.37 (s, 3H), 3.24 (s, 3H), 3.05 (s, 2H), 2.72 (s, 3H).^13^C NMR (126 MHz, DMSO-*d*_6_) δ 169.92, 163.53, 161.65, 160.35, 160.23, 154.29, 149.19, 148.09, 141.27, 136.65, 133.52, 129.95, 129.32, 129.04, 127.58, 122.77, 120.61, 107.94, 106.65, 103.26, 98.29, 97.57, 70.93, 58.42, 42.94, 33.79, 11.93. HRMS: *m*/*z* C_31_H_21_N_7_O_4_Na [M + Na]^+^ 588.2438, Found 588.2337. Purity: >95% (HPLC).

1,5-dimethyl-*N*-(4-((2-((3-((1-methylpiperidin-4-yl)oxy)phenyl)amino)pyrimidin-4-yl)oxy)phenyl)-3-oxo-2-phenyl-2,3-dihydro-1*H*-pyrazole-4-carboxamide (compound **17c**): White solid, yield: 7.01%. ^1^H NMR (500 MHz, DMSO-*d*_6_) δ 10.81 (s, 1H), 9.51 (s, 1H), 8.35 (d, *J* = 5.5 Hz, 1H), 7.68 (d, *J* = 8.7 Hz, 2H), 7.60 (t, *J* = 7.5 Hz, 2H), 7.52 (t, *J* = 7.4 Hz, 1H), 7.45 (d, *J* = 7.5 Hz, 2H), 7.27 (s, 1H), 7.20 (d, *J* = 8.7 Hz, 2H), 7.16 (d, *J* = 7.8 Hz, 1H), 7.00 (t, *J* = 8.0 Hz, 1H), 6.51 (d, *J* = 7.7 Hz, 1H), 6.42 (d, *J* = 5.5 Hz, 1H), 4.30 (s, 1H), 3.34 (s, 2H), 2.93 (s, 2H), 2.72 (s, 3H), 2.45 (s, 3H), 2.00 (s, 2H), 1.76 (s, 2H).^13^C NMR (126 MHz, DMSO-*d*_6_) δ 169.95, 163.51, 161.64, 160.33, 160.13, 157.35, 154.28, 147.98, 141.90, 136.69, 133.50, 129.96, 129.49, 129.33, 127.59, 122.70, 120.68, 112.14, 108.92, 107.25, 98.85, 97.57, 51.75, 44.37, 33.80, 31.62, 30.30, 11.94. HRMS: *m*/*z* C_34_H_36_N_7_O_4_ [M + H]^+^ 606.2751, Found 606.2823. Purity: >95% (HPLC).

1,5-dimethyl-*N*-(4-((2-((4-(4-methylpiperazine-1-carbonyl)phenyl)amino)pyrimidin-4-yl)oxy)phenyl)-3-oxo-2-phenyl-2,3-dihydro-1*H*-pyrazole-4-carboxamide (compound **17d**): White solid, yield: 63.0%. ^1^H NMR (500 MHz, DMSO-*d*_6_) δ 10.85 (s, 1H), 9.82 (s, 1H), 8.38 (d, *J* = 5.6 Hz, 1H), 7.71 (d, *J* = 8.8 Hz, 2H), 7.60 (t, *J* = 7.6 Hz, 2H), 7.49–7.56 (m, 3H), 7.45 (d, *J* = 7.3 Hz, 2H), 7.21 (d, *J* = 8.8 Hz, 2H), 7.13 (d, *J* = 8.3 Hz, 2H), 6.51 (d, *J* = 5.6 Hz, 1H), 3.37 (s, 4H), 3.33 (s, 3H), 2.73 (s, 3H), 2.27 (s, 4H), 2.15 (s, 3H).^13^C NMR (126 MHz, DMSO-*d*_6_) δ 170.17, 169.40, 163.51, 161.67, 160.46, 159.84, 154.35, 148.05, 141.99, 136.85, 133.49, 129.95, 129.30, 128.45, 128.17, 127.57, 123.12, 120.68, 118.37, 99.11, 97.65, 54.90, 33.80, 11.98. HRMS: *m*/*z* C_34_H_34_N_8_O_4_Na [M + Na]^+^ 641.2703, Found 641.2603. Purity: >95% (HPLC).

*N*-(4-((2-((4-(4-acetylpiperazin-1-yl)phenyl)amino)pyrimidin-4-yl)oxy)phenyl)-1,5-dimethyl-3-oxo-2-phenyl-2,3-dihydro-1*H*-pyrazole-4-carboxamide (compound **17e**): White solid, yield: 15.79%. ^1^H NMR (500 MHz, DMSO-*d*_6_) δ 10.85 (s, 1H), 9.36 (s, 1H), 8.28 (d, *J* = 5.5 Hz, 1H), 7.69 (d, *J* = 8.7 Hz, 2H), 7.60 (t, *J* = 7.6 Hz, 2H), 7.52 (t, *J* = 7.4 Hz, 1H), 7.47 (d, *J* = 7.4 Hz, 2H), 7.26 (s, 2H), 7.16 (d, *J* = 8.8 Hz, 2H), 6.68 (d, *J* = 6.9 Hz, 2H), 6.38 (d, *J* = 5.5 Hz, 1H), 3.48 (s, 4H), 3.40 (s, 3H), 2.99 (s, 2H), 2.87 (s, 2H), 2.74 (s, 3H), 2.00 (s, 3H).^13^C NMR (126 MHz, DMSO-*d*_6_) δ 170.26, 168.66, 163.48, 161.69, 160.29, 160.03, 154.04, 148.29, 145.98, 136.72, 133.47, 133.25, 129.97, 129.38, 127.69, 123.28, 120.82, 120.48, 116.55, 97.44, 50.18, 49.24, 45.98, 33.75, 21.60, 11.88. HRMS: *m*/*z* C_34_H_34_N_8_O_4_Na [M + Na]^+^ 641.2703, Found 641.2607. Purity: >95% (HPLC).

*N*-(4-((2-((3-(4-acetylpiperazin-1-yl)phenyl)amino)pyrimidin-4-yl)oxy)phenyl)-1,5-dimethyl-3-oxo-2-phenyl-2,3-dihydro-1*H*-pyrazole-4-carboxamide (compound **17f**): White solid, yield: 20.0%. ^1^H NMR (500 MHz, DMSO-*d*_6_) δ 10.82 (s, 1H), 9.40 (s, 1H), 8.34 (d, *J* = 5.5 Hz, 1H), 7.68 (d, *J* = 8.8 Hz, 2H), 7.60 (t, *J* = 7.6 Hz, 2H), 7.52 (t, *J* = 7.4 Hz, 1H), 7.45 (d, *J* = 7.4 Hz, 2H), 7.19 (d, *J* = 8.5 Hz, 3H), 7.09 (d, *J* = 7.3 Hz, 1H), 6.96 (t, *J* = 8.0 Hz, 1H), 6.51 (d, *J* = 7.5 Hz, 1H), 6.41 (d, *J* = 5.5 Hz, 1H), 3.51 (t, *J* = 5.7 Hz, 4H), 3.37 (s, 4H), 2.96 (s, 4H), 2.72 (s, 3H), 2.01 (s, 3H).^13^C NMR (126 MHz, DMSO-*d*_6_) δ 169.84, 168.70, 163.53, 161.64, 160.33, 160.26, 154.27, 151.57, 148.05, 141.31, 136.61, 133.51, 129.95, 129.32, 129.14, 127.60, 122.59, 120.57, 111.11, 110.15, 107.18, 98.73, 97.56, 49.29, 48.88, 45.86, 33.78, 21.63, 11.92. HRMS: *m*/*z* C_34_H_34_N_8_O_4_Na [M + Na]^+^ 641.2703, Found 641.2599. Purity: >95% (HPLC).

1,5-dimethyl-*N*-(4-((2-((6-(2-(4-methylpiperazin-1-yl)acetamido)pyridin-3-yl)amino)pyrimidin-4-yl)oxy)phenyl)-3-oxo-2-phenyl-2,3-dihydro-1*H*-pyrazole-4-carboxamide (compound **17g**): White solid, yield: 18.6%. ^1^H NMR (600 MHz, DMSO-*d*_6_) δ 10.81 (s, 1H), 9.72 (s, 1H), 9.67 (s, 1H), 8.49 (s, 1H), 8.35 (d, *J* = 5.6 Hz, 1H), 7.98 (s, 1H), 7.88 (d, *J* = 8.6 Hz, 1H), 7.69 (d, *J* = 8.9 Hz, 2H), 7.60 (t, *J* = 7.7 Hz, 2H), 7.52 (t, *J* = 7.5 Hz, 1H), 7.48–7.43 (m, 2H), 7.20 (d, *J* = 8.9 Hz, 2H), 6.43 (d, *J* = 5.6 Hz, 1H), 3.37 (s, 5H), 3.11 (s, 2H), 2.72 (s, 3H), 2.52–2.50 (m, 2H), 2.38 (s, 4H), 2.18 (s, 3H).^13^C NMR (126 MHz, DMSO-*d*_6_) δ 170.10, 168.61, 163.52, 161.66, 161.66, 160.40, 160.08, 154.31, 147.90, 145.69, 139.23, 136.78, 133.75, 133.50, 129.95, 129.32, 128.77, 127.57, 122.68, 120.68, 113.01, 99.15, 97.62, 61.50, 55.13, 52.97, 46.02, 33.78, 11.94. HRMS: *m*/*z* C_34_H_36_N_10_O_4_Na [M + Na]^+^ 671.2921, Found 671.2823. Purity: >95% (HPLC).

1,5-dimethyl-*N*-(4-((2-((4-(2-(4-methylpiperazin-1-yl)propanamido)phenyl)amino)pyrimidin-4-yl)oxy)phenyl)-3-oxo-2-phenyl-2,3-dihydro-1*H*-pyrazole-4-carboxamide (compound **17h**): White solid, yield: 33.8%. ^1^H NMR (600 MHz, DMSO-*d*_6_) δ 10.81 (s, 1H), 9.55 (s, 1H), 9.49 (s, 1H), 8.32 (d, *J* = 5.6 Hz, 1H), 7.71–7.67 (m, 2H), 7.60 (t, *J* = 7.7 Hz, 2H), 7.52 (dt, *J* = 8.3, 1.5 Hz, 1H), 7.51–7.47 (m, 2H), 7.45–7.42 (m, 2H), 7.37 (d, *J* = 8.6 Hz, 2H), 7.22–7.18 (m, 2H), 6.37 (d, *J* = 5.6 Hz, 1H), 3.37 (s, 3H), 3.15 (q, *J* = 6.8 Hz, 1H), 2.72 (s, 3H), 2.52–2.50 (m, 2H), 2.40 (d, *J* = 71.7 Hz, 6H), 2.13 (s, 3H), 1.13 (d, J = 6.9 Hz, 3H).^13^C NMR (126 MHz, DMSO-*d*_6_) δ 171.20, 170.01, 163.52, 161.65, 160.31, 160.17, 154.32, 148.03, 136.71, 133.49, 133.13, 129.95, 129.31, 127.53, 122.77, 120.64, 120.16, 119.56, 98.41, 97.65, 63.77, 55.38, 49.39, 46.16, 33.78, 13.29, 11.94. HRMS: *m*/*z* C_36_H_40_N_9_O_4_ [M + H]^+^ 662.3125, Found 662.3207. Purity: >95% (HPLC).

### 2.2. Inhibitory Activity Assessment Against Mer and c-Met

In vitro Mer/c-Met kinase inhibition profiling was outsourced to Shanghai Bioduro Biological Technology Co., Ltd. (Shanghai, China) using the following optimized protocol: Assay buffer (1X, MgCl_2_ 5 mM, DTT 1 mM) served as the base matrix. Test compounds were diluted in DMSO to 100× final concentration and dispensed (100 nL/well) into a 384-well plate via an automated liquid handler, maintaining 1% DMSO% throughout. Enzymes were diluted to a 2X assay working concentration (1.2 nM) in buffer. Then, 5 μL was added to the assay plate manually (final 1 nM) with a multichannel pipette, span down at 1000 rpm and centrifuged for 30 s, and incubated for 15 min at 25 °C temperatures. The substrate solutions were diluted with 1X assay buffer. Then, 5 μL of mix or buffer was added to the assay plate manually (ATP final 3.5 μM and TK-Sub-Biotin final 0.5 μM) with a multichannel pipette, span down at 1000 rpm and centrifuged for 30 s. After 30 °C 60 min, 10 μL of detection solution (TK-antidody-cryptate final 0.25X and streptavidine-XL 665 final 0.3125 μM) was added to each well of the assay. This was mixed briefly with centrifuge and equilibrated for 60 min. The luminescence was recorded on Envision.

### 2.3. Antiproliferation Assay

The HepG2, MDA-MB-231, and HCT116 cell lines were derived from our own laboratory (the laboratory of our collaborator, Dr. Xiaolei Zhou, at College of Food Science and Biology in Hebei University of Science and Technology) [31]. The antiproliferative activities were assessed using the CCK-8 assay. Cell viability was measured according to the manufacturer’s instructions for the Cell Counting Kit-8 (Meilunbio, Dalian, China). In this assay, 2.5 × 10^3^ cells per well were seeded into 96-well plates.

After an initial 12 h incubation, cancer cells were exposed to varying concentrations of compound **17c** for 72 h. Following CCK-8 solution addition and 2 h incubation, the absorbance at 450 nm was measured using a BioTek Synergy HTX microplate reader. Experiments included triplicate wells with three independent replicates. The IC_50_ values for compound **17c** were derived from an analysis of the dose–response curves in GraphPad PRISM 9.5.

### 2.4. hERG Potassium Channel Activity Assessment

hERG channel activity was evaluated in CHO cells (Cell Bank of Chinese Academy of Sciences, SCSP-507) stably expressing hERG transcripts using Sophion’s QPatch automated patch clamp system (Shanghai Institute of Materia Medica, Shanghai, China). Cells cultured in serum-supplemented F12 medium (37 °C/5% CO_2_) to 70–80% confluency were PBS-washed, detached with Detachin (37 °C/2 min), and resuspended in growth medium (2–5 × 10^6^ cells/mL) before QPatch loading. Following centrifugation and washing, recording was performed using extracellular solution (140 NaCl, 5 KCl, 1 CaCl_2_, 1.25 MgCl_2_, 10 HEPES and 10 Glucose, pH 7.4 with NaOH) and intracellular solution (140 KCl, 1 MgCl_2_, 1 CaCl_2_, 10 EGTA and 10 HEPES, pH 7.2 KOH). A voltage protocol measured tail current peaks at −50 mV. After a 5 min baseline recording, compounds were applied for 2.5 min/concentration across six escalating doses (*n* ≥ 3 cells/dose), with terminal cisapride reference. The IC_50_ values were derived from concentration–inhibition curves (four-parameter logistic fit, GraphPad Prism 8) using Assay Software v5.6.4 for acquisition and Excel for supplementary analysis.

### 2.5. Liver Microsome Stability Assay

Each test compound was initially dissolved in DMSO to prepare a 10 mM stock solution. Test compound and positive controls were pre-diluted in acetonitrile to 200 μM. Incubation mixtures (200 μL total volume) comprised 0.1 M PBS (pH 7.4), 2 mM of NADPH, 0.2 mg/mL liver microsomes, and the diluted compound/control. Following a 5 min pre-incubation of all components (excluding NADPH) at 37 °C, reactions were initiated withNADPH addition. The mixture was pipette-mixed thoroughly, and 20 μL was immediately transferred to a “Quenching” plate as a 0 min sample, followed by pipette-mixing. At subsequent time points (5, 15, 30, and 60 min), 20 μL aliquots were transferred from the incubate to the “Quenching” plate after pipette-mixing. To each well of the plate, 200 μL of acetonitrile containing the internal standard was added. After centrifugation (4000 rpm, 10 min), 50 μL of supernatant was diluted 1:1 with deionized water and subjected to LC-MS/MS analysis.

### 2.6. Docking Study

Molecular docking simulations were performed in our lab. The binding interactions of the compounds were investigated with the aid of Discovery Studio 2019. For this analysis, the X-ray crystallographic structures of MerTK (PDB: 4M3Q) and c-Met (PDB: 3LQ8), both resolved at 1.84 Å resolution, were utilized. The protein–ligand interactions were visualized and analyzed through the interaction mode diagram generated by Discovery Studio 2019.

### 2.7. Western Blot Assay

Western blot assay was performed according to established protocols. HCT colon cancer cells were exposed to the specified concentration of compound **17c** for 24 h, after which whole cell lysates were prepared using RAPI cell lysis buffer (Beyotime Institute of Biotechnology, Shanghai, China). Primary antibodies, purchased from Beyotime Institute of Biotechnology, were diluted at a ratio of 1:2000. The protein bands on polyvinylidene fluoride membranes were visualized and analyzed using Glyco Band-Scan Software Version 4.50. The antibody details are as follows:

Name: Phospho-MER/TYRO3 (Tyr753/Tyr685) antibody, Species: Rabit, Sequence: synthesized peptide derived from human MER/TYRO3 around the Phosphorylation site of Tyr753/Tyr685, Dilution: 1:2000, RRID number: AB_2840500, Catalogue number: AF8443, Supplier: Affinity Biosciences (Beijing, China).

Name: Phospho-c-Met (Tyr1234) antibody, Species: Rabit, Sequence: synthesized peptide derived from human c-Met around the Phosphorylation site of Tyr1234, Dilution: 1:2000, RRID number: AB_2834564, Catalogue number: AF3129, Supplier: Affinity Biosciences (Beijing, China).

### 2.8. Apotosis Study

Apoptosis was analyzed in a dose-dependent manner using the One Step TUNEL Apoptosis Assay Kit (Meilunbio, Dalian, China). HCT116 cells (1.5 × 10^4^ cells/well) were seeded onto TC-treated glass coverslips in 24-well plates and incubated for 24 h. Following this, the cells were exposed to varying concentrations of compound **17c** for 48 h. After treatment, the cells were fixed with 4% paraformaldehyde and permeabilized with 0.3% Triton-X-100. TUNEL staining was performed as per the manufacturer’s guidelines, with DAPI used for nuclear counterstaining. Fluorescent images were obtained using an Olympus microscope. The ED_50_ value for compound **17c** was calculated using GraphPad PRISM 9.5. Each assay was performed in triplicate and repeated three times for consistency.

### 2.9. Transwell Assay

Migration assays were conducted using Transwell^®^ inserts (Costar, Cambridge, MA, USA) with an 8 μm pore size polycarbonate membrane. HCT116 cells (1.5 × 10^5^) were seeded into the upper chamber with serum-free medium containing 0.2% BSA, while the lower chamber contained medium supplemented with 15% FBS. Compound **17c** was applied at the specified concentrations to both sides of the membrane. After 24 h of incubation at 37 °C, the cells were fixed in methanol and stained with 0.1% crystal violet. Non-migrated cells on the upper surface of the membrane were removed using a cotton swab. Five random fields per well were imaged under brightfield microscopy, and the number of migrated cells was quantified using ImageJ software (1.53c). Each experiment was performed in triplicate.

### 2.10. Plasma Protein Binding in Plasma

A stock solution (20 mM) of compound **17c** was prepared in DMSO. The stock solution was then diluted into 100 μM with acetonitrile. The HT Dialysis apparatus was assembled according to the manufacturer’s instructions. Then, 150 μL of the plasma spiked with 1 μM final compound concentrations was added to one side of the well and 150 μL of PBS was added to the opposite side of the well (n = 3). The plate was equilibrated for 6 h at 37 °C in a 5% CO_2_ incubator. For T0 samples, 200 μL of spiked plasma was stored at –20 °C. For T6 samples, 200 μL of spiked plasma was incubated at 37 °C in a 5% CO_2_ incubator for 6 h. After incubation, 50 μL of the samples (plasma or PBS) was collected into a vessel containing 50 μL of the opposite matrix. Then, 300 μL of acetonitrile with IS was added into the vessels. The 96-well plate was centrifuged at 4000 rpm for 10 min. Then, 50 μL of supernatant was mixed with 50 μL of ddH_2_O and then injected onto the LC-MS/MS system for analysis.

### 2.11. Pharmacokinetic Properties

Male and female SD rats (SLRC Laboratory Animal Inc., Shanghai, China) were used for the study. In the oral (PO) administration, 3 rats per group were given a single 10 mg/kg dose. Blood samples (100 μL per time point) were collected via jugular vein cannula and taken at 5, 15, and 30 min, and 1, 2, 4, 8, and 24 h post-dose. For the intravenous (IV) administration, 3 rats per group received 2.0 mg/kg, and blood samples were collected at 5, 15, and 30 min, and 1, 2, and 4 h after dosing. Rats were fasted overnight prior to dosing and remained fasted for 6 h post-dose. Plasma was separated by centrifugation and stored at –40 °C until analysis. Compound concentrations were determined by LC/MS/MS (Shimadzu LC-30AD), and pharmacokinetic parameters, including C_max_, T_max_, T_1/2_, and AUC_0-inf_, were calculated.

## 3. Results and Discussion

### 3.1. Chemistry

**Scheme 1:**
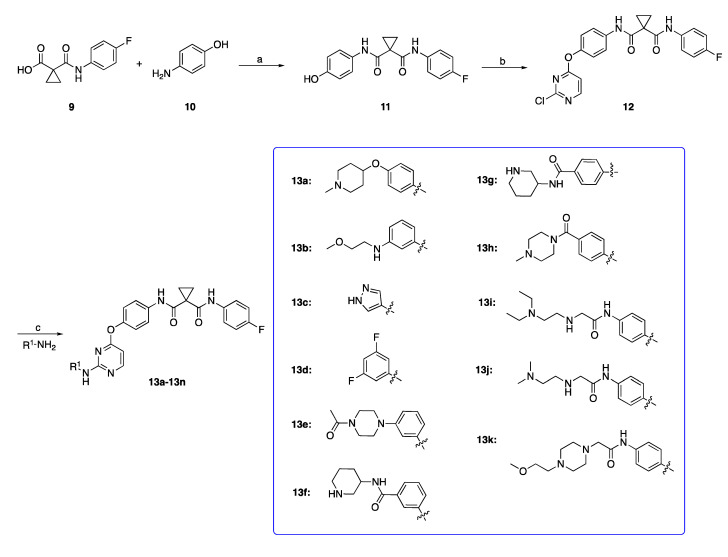
Reagents and conditions: (**a**) EDC·HCl/DMF, r.t., 6 h; (**b**) 2,4-dichloropyrimidine, K_2_CO_3_/DMF, 80 °C, 6 h; (**c**) PTSA/DMF, 90 °C, 4 h.

**Scheme 2:**
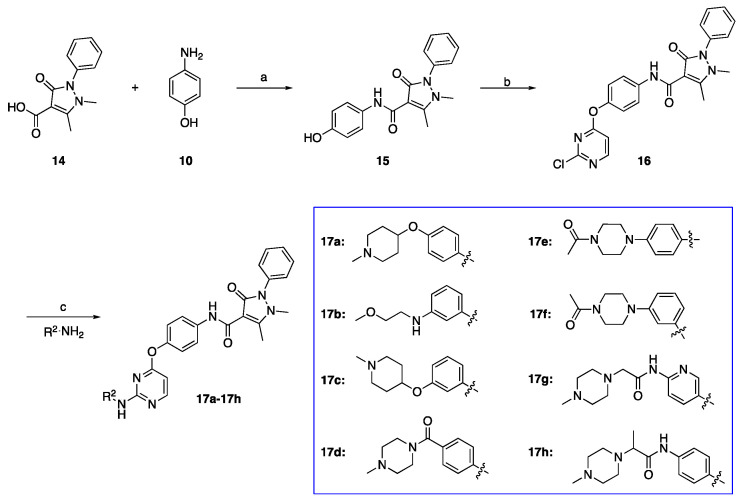
Reagents and conditions: (**a**) HBTU/TEA/DMF, r.t., 8 h; (**b**) 2,4-dichloropyrimidine, K_2_CO_3_/DMF, 80 °C, 4.5 h; (**c**) PTSA/DMF, 90 °C, 4.0 h.

The synthesis of target compounds and the key intermediates are described in Scheme 1 and Scheme 2, respectively. The structures of compounds were confirmed by ^1^H-NMR, ^13^C-NMR and HRMS spectroscopy.

As depicted In Scheme 1, a condensation reaction where 1-((4-fluorophenyl)carbamoyl)cyclopropane-1-carboxylic acid (**9**) was combined with readily accessible 4-aminophenol (**10**) at ambient temperature was employed, yielding the intermediate *N*-(4-fluorophenyl)-*N*-(4-hydroxyphenyl)cyclopropane-1,1-dicarboxamide (**11**) [36]. Subsequently, the intermediate *N*-(4-((2-chloropyrimidin-4-yl)oxy)phenyl)-N-(4-fluorophenyl)cyclopropane-1,1-dicarboxamide (**12**) was produced via an S_N_2 reaction between intermediate **11** with 2,4-dichloropyrimidine [37]. Finally, the desired compounds **13a**–**13k** were obtained by reacting intermediate **12** with various substituted anilines, which were either commercially available or prepared in our prior research [38].

In Scheme 2, the preparation of the crucial intermediate *N*-(4-hydroxyphenyl)-1,5-dimethyl-3-oxo-2-phenyl-2,3-dihydro-1*H*-pyrazole-4-carboxamide (**15**) commenced with a condensation process involving 1,5-dimethyl-3-oxo-2-phenyl-2,3-dihydro-1*H*-pyrazole-4-carboxylic acid (**14**) and 4-aminophenol (**10**) [32]. Intermediate **15** was then reacted with 2,4-dichloropyrimidine, yielding the pivotal intermediate *N*-(4-((2-chloropyrimidin-4-yl)oxy)phenyl)-1,5-dimethyl-3-oxo-2-phenyl-2,3-dihydro-1*H*-pyrazole-4-carboxamide (**16**). The target compounds **17a**–**17h** were subsequently obtained by carrying out a nucleophilic substituted reaction between intermediate **16** and various substituted anilines.

### 3.2. Kinase Inhibitory Activities

All the target compounds (Supplementary Material, S1) were initially screened for their inhibitory activities on Mer and c-Met kinases compared to the lead compound (**18c**), and the results are depicted in Table 1 and Table 2. The corresponding curves are shown in Supplementary S3 and S4.

As summarized in Table 1, the substituents (R^1^) at the terminal pyrimidine amine group significantly modulate inhibitory potency against Mer and c-Met kinases. Compounds **13b**, **13j**, and **13k** exhibited moderate to potent dual inhibitory, with Mer IC_50_ values ranging from 13.1 ± 2.1~37.3 ± 10.6 nM and c-Met IC_50_ values of 25.9 ± 7.7~48.6 ± 11.2 nM, compared to the lead compound **18c** (Mer IC_50_: 18.5 ± 2.3 nM, c-Met IC_50_: 33.6 ± 4.3 nM). Strikingly, compound **13e** demonstrated the highest dual potency, achieving IC_50_ values of 7.5 ± 1.6 nM (Mer) and 7.5 ± 1.1 nM (c-Met), representing a 2.5-fold and 4.5-fold improvement over **18c**, respectively. In contrast, compounds **13c** and **13h** showed reduced Mer inhibitory activity while maintaining c-Met inhibitory activity comparable to **18c**. Conversely, compounds **13d** and **13i** exhibited significantly reduced inhibitory activities against both Mer and c-Met kinase.

A novel series of pyrimidine derivatives were designed to optimize dual Mer/c-Met inhibition. As shown in Table 2, the Mer kinase inhibitory activities varied significantly across the series. Compounds **17d**, **17e**, and **17g** exhibited reduced inhibitory potency, with Mer IC_50_ values of 84.1 ± 13.9, 69.8 ± 12.5, and >1000 nM, respectively. In contrast, compounds **17a**, **17b**, and **17h** retained activity comparable to the lead compound, featuring IC_50_ values of 26.2 ± 7.1, 12.1 ± 1.3, and 11.9 ± 1.7 nM. Notably, compounds **17c** and **17f** showcased superior inhibition, achieving Mer IC_50_ values of 6.4 ± 1.8 nM and 5.5 ± 0.8 nM.

For c-Met kinase inhibition, compounds **17c**, **17f**, and **17h**, exhibited comparable IC_50_ values ranging from 25.0 ± 3.5 to 45.0 ± 6.8 nM. However, the potency of **17a**, **17b**, **17d**, **17e**, and **17g** decreased (IC_50_ values from 100.7 ± 18.5 nM to 235.6 ± 25.6 nM), indicating that specific structural optimization at the pyrimidine scaffold detrimentally impacts c-Met binding.

**Figure 6:**
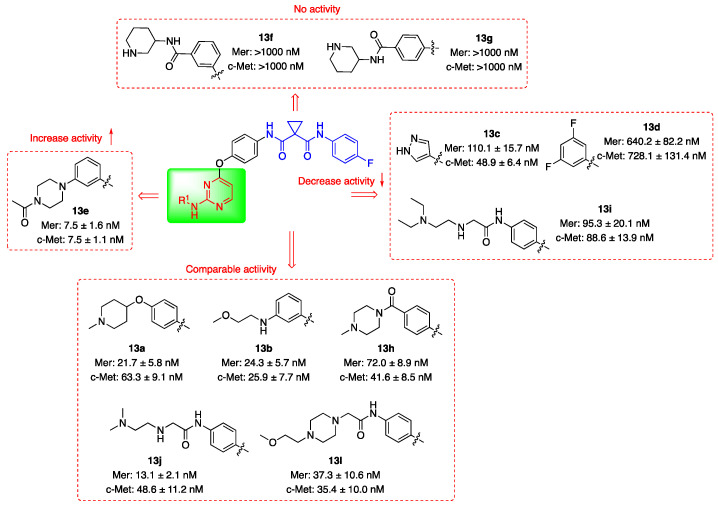
SAR study of the designed compounds with side chain A.

**Figure 7:**
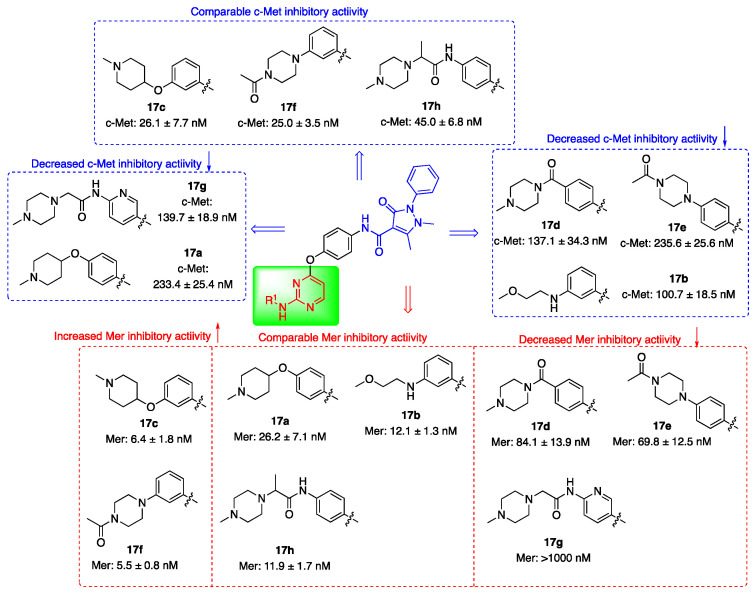
SAR study of the designed compounds with side chain B.

The findings converge to demonstrate that the designed compounds exhibit robust inhibitory effects on both Mer and c-Met targets. The steric bulk, electronic properties, and hydrophobicity of the R^1^ substituents have a decisive impact on dual activity. The R^1^ structure of **13e** likely optimizes interactions with the kinase ATP pocket (e.g., hydrogen bonding, hydrophobic packing), whereas the R^1^ groups in **13d** and **13i** may introduce steric hindrance or disrupt critical binding interactions. Modification of the pyridine ammine scaffold can selectively enhance Mer inhibition (e.g., **17c/f**), while most modifications (e.g., 17a/b/d/e/g) weaken c-Met binding, suggesting that c-Met is more sensitive to structural alterations. There may be local differences in the ATP-binding pockets of Mer and c-Met (e.g., flexibility or key amino acids), which could result in differing structural optimization on the two targets. A general structure–activity relationship can be visualized in Figure 6 and Figure 7. While the designed compounds demonstrate potent Mer kinase inhibition, their inhibitory activity still falls short of the positive control, indicating the need for further optimization to improve potency.

### 3.3. In Vitro Stability in Liver Microsomes

Metabolic stability is a critical determinant of drug-like properties. To prioritize compounds for further development, we evaluate the human liver microsomal stability of selected candidates demonstrating potent dual Mer/c-Met dual inhibition in vitro. As shown in Table 3, compounds **13b**, **13e**, **13j**, **13k**, **17c**, and **17f** were subjected to microsome assays to determine their half-life and intrinsic clearance. Compound **17c** demonstrated superior metabolic stability, with an extended t_1/2_ of 147.0 min and low CLint of 0.026 mL/min/mg, whereas **17f** exhibited a shorter t_1/2_ of 51.0 min and higher CLint of 0.068 mL/min/mg. Notably, all other compounds showed rapid hepatic clearance (t_1/2_ < 40 min, CL_int_ > 0.068 mL/min/mg), suggesting limited metabolic resistance. The results highlight **17c** as the most metabolically stable candidate within this series.

### 3.4. Antiproliferation Assay In Vitro

Compound **17c** was further evaluated for its antiproliferative activity against three cancer cell lines with high Mer and c-Met expression (HCT116 colon cancer, CAKI-1 renal cancer, and PC-3 prostate cancer) using the CCK-8 assay [5,39,40,41,42]. As summarized in Table 4, **17c** demonstrated superior antiproliferative activity compared to cabozantinib across all tested cell lines. Notably, **17c** exhibited the greatest potency with HCT116 cells, with an IC_50_ value of 0.46 ± 0.06 μmol/L—a 17.1-fold improvement over cabozantinib. In CAKI-1 cells, **17c** showed an IC_50_ of 2.31 ± 0.67 μmol/L, representing a 2.0-fold enhancement compared to cabozantinib. Similarly, against PC-3 cells, **17c** achieved an IC_50_ of 3.79 ± 1.09 μmol/L, which is 4.1 times more effective than cabozantinib. These results collectively establish compound **17c** as a potent dual Mer/c-Met inhibitor with significant therapeutic potential. The dose–response curves are provided in Supplementary S5.

### 3.5. Molecular Docking Study of Compound **17c**

**Figure 8:**
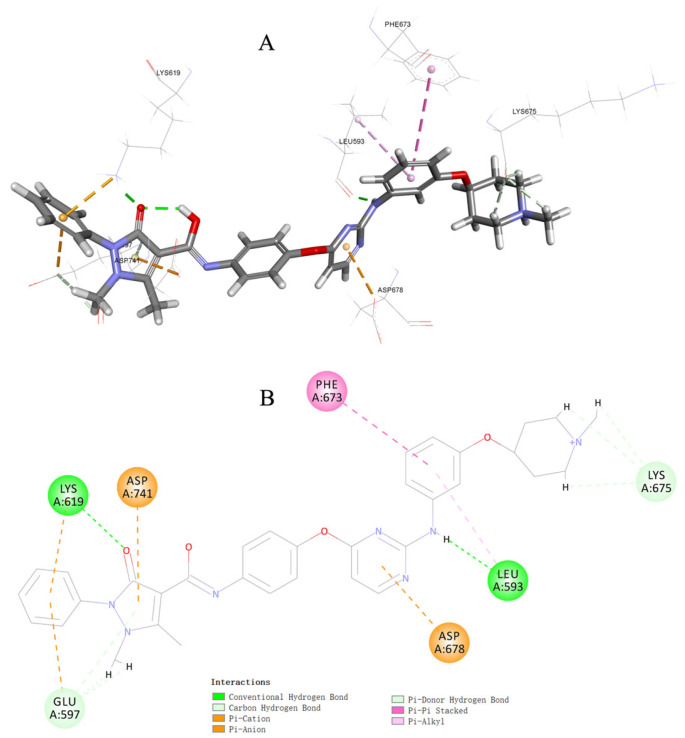
**17c** binding mode in Mer kinase (PDB: 3LQ8), (**A**) 3D ligand–protein interactions; (**B**) 2D ligand interaction diagram.

**Figure 9:**
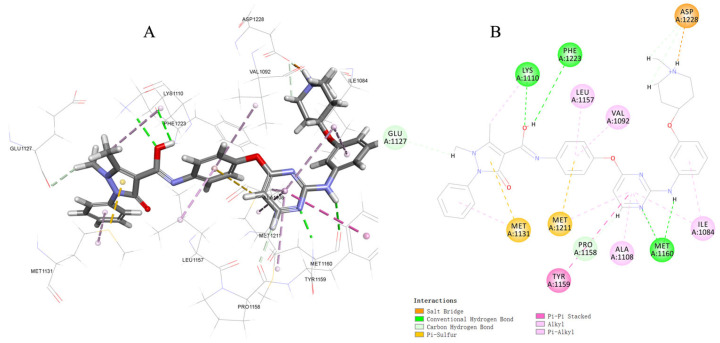
**17c** binding mode in c-Met kinase (PDB: 3LQ8), (**A**) 3D ligand–protein interactions; (**B**) 2D ligand interaction diagram.

Molecular docking was conducted to validate the binding mode of compound **17c** with Mer kinase (PDB: 4M3Q) and c-Met (PDB: 3LQ8) kinases. As illustrated in Figure 8, the docking pose of **17c** in Mer kinase revealed three critical interactions: (1) the aminopyrimidine moiety acts as hydrogen bond acceptor with Leu593; (2) the amide side chain forms a hydrogen bond with Lys619; and (3) the benzene ring of the side chain engages in a π-π stacking interaction. The docking interaction energy was −68.7952 KJ/mol. For c-Met kinase (Figure 9), **17c** exhibited a similar binding mode: (1) the aminopyrimidine core forms a hydrogen bond with Met1160; (2) the amide group forms hydrogen bonds with Lys1110 and Phe1223; and (3) the pyrimidine group participates in π-π interaction. The docking interaction energy was −91.7674 KJ/mol. These results confirm that the 2-substitutedaniline pyrimidine group effectively occupies the ATP-binding pockets of both kinases through conserved hydrogen bonding and hydrophobic interactions. The structural coherence between the predicted binding modes and enzymes inhibitory data strongly supports the rational design strategy, validating the scaffold’s potential for dual Mer/c-Met inhibition.

We also systematically compared our compound’s docking results with that of cabozantinib and foretinib (XL880). For c-Met kinase, the docking results of cabozantinib with c-Met kinase showed that the quinoline core could form a hydrogen bond with Met1160, and that the amide group participates in hydrogen bonding with Lys1110 and Asp1222. The docking results of foretinib with c-Met showed that the quinoline core could form a hydrogen bond with Met1160, and that the amide group participates in hydrogen bonding with Lys1110 and Asp1222. The docking results confirm that compound **17c** shares a conserved binding mode with both the reference drug cabozantinib and the co-crystallized c-Met inhibitor foretinib (PDB: 3LQ8).

For Mer kinase, the binding mode of cabozantinib with Mer kinase remains unreported, and co-crystallized Mer ligands possess distinct chemotypes. We conducted the docking of cabozantinib and **17c** with Mer kinase, and the results showed that cabozantinib’s amide group forms hydrogen bonds with the hinge region (Pro672 and Met674), while the quinoline moiety occupies a hydrophobic cavity without direct hydrogen bonding. Compound **17c**’s aminopyrimidine moiety acts as a hydrogen bond acceptor with Leu593 and the amide side chain forms a hydrogen bond with Lys619. The docking results showed that compound **17c** possesses better affinity than that of the reference drug cabozantinib.

### 3.6. Western Blot Assay

**Figure 10:**
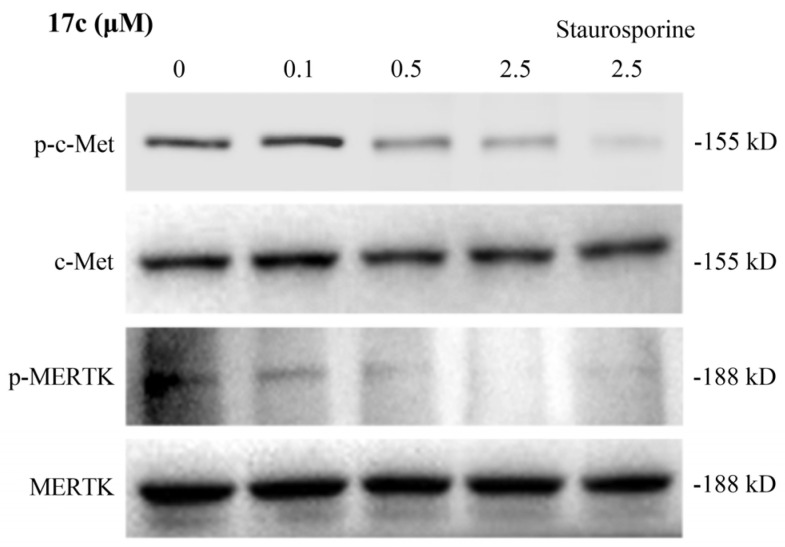
Western blot analysis was applied to analyze the expression and phosphorylation of c-Met and MERTK in HCT116 cells with staurosporine or increasing doses of compound **17c**. All experiments were performed at least three times. Original Western blot images can be found in Figure S85.

The inhibitory effects of compound **17c** on Mer and c-Met kinase phosphorylation were assessed using Western blot analysis. As shown in Figure 10, increasing concentrations of **17c** resulted in a reduction in the phosphorylation levels of both Mer and c-Met kinases. At a concentration of 2.5 μM, **17c** significantly inhibited the phosphorylation of these kinases compared to the positive control, demonstrating its potential to effectively block Mer and c-Met kinase activation.

### 3.7. The hERG Test

To evaluate the potential cardiotoxicity risk associated with compound **17c**, we assessed its inhibitory activity against the hERG potassium channel, a key determinant of cardiac safety [33]. As summarized in Table 5, **17c** demonstrated negligible hERG inhibition (IC_50_ > 40 μM), indicating that a low risk of hERG activity was observed despite its potent dual inhibition of Mer and c-Met kinases.

### 3.8. Apoptosis Assay

**Figure 11:**
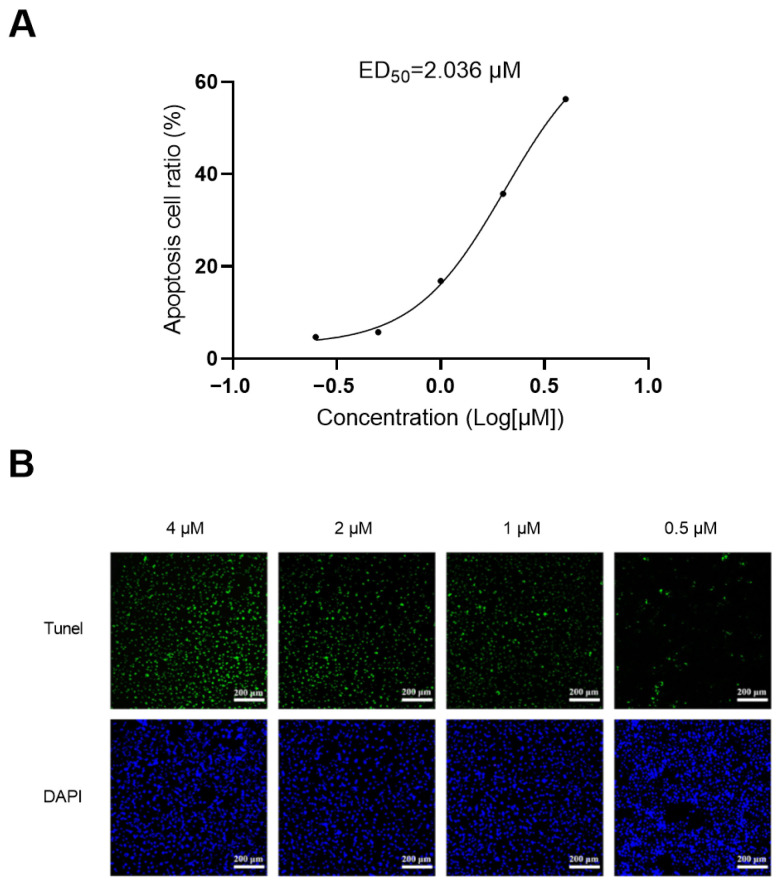
Compound **17c** induced cell apoptosis in HCT116 cancer cells. (**A**) Quantitative analysis of TUNEL staining. (**B**) Representative TUNEL and DAPI-stained images. Magnification, x100. HCT116 cells grown on cover slip, treated with various indicated doses of **17c** for 48 h, and stained for TUNEL (green). The number of TUNEL-positive cells was counted from five non-overlap random fields per group. DAPI (blue). Data are representative of three independent experiments.

To evaluate the apoptosis-inducing capability of compound **17c**, the HCT116 colon cancer cell line was treated with varying concentrations of the compound. Apoptosis was evaluated using the TUNEL assay. DAPI staining (blue) served as the positive control, while apoptotic cells were identified by TUNEL staining (green) after 48 h of incubation with compound **17c**, as shown in Figure 11. The quantity of apoptotic cells was measured at different concentrations of **17c**, resulting in an ED_50_ value of 2.036 μM. These results demonstrate that compound **17c** efficiently triggers apoptosis in HCT116 cells, thereby emphasizing its potential as a highly promising candidate for additional research and development.

### 3.9. Transwell Assay

**Figure 12:**
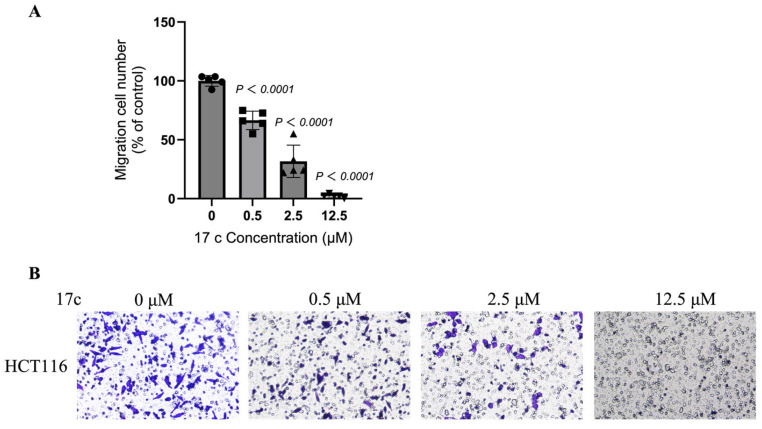
Compound **17c** inhibited cell migration in HCT116 cancer cells. (**A**) Quantification of transwell assays. (**B**) Representative images of transwell assay of HCT116 cells treated with compound **17c** for 24 h.

The Transwell migration assay shown in Figure 12 was performed to evaluate the effect of compound **17c** on the migration of HCT116 cells. A decrease in the number of cells migrating through the membrane was observed with an increasing concentration of **17c**. These results suggest that compound **17c** effectively inhibits the migration capacity of HCT116 colon cancer cells in a concentration-dependent manner.

### 3.10. Plasma Protein-Binding Affinity

The plasma protein-binding (PPB) affinity significantly influences the compound’s free fraction, bioavailability, tissue distribution, and elimination kinetics, thereby impacting the pharmacokinetic properties. The PPB profile of compound **17c** was assessed via equilibrium dialysis, with warfarin serving as a positive control. As shown in Table 6, **17c** exhibited high species-independent protein binding, with unbound fractions of 1.58% in human plasma and 1.78% in mouse plasma, corresponding to PPB values of 98.4% and 98.2%, respectively. Combined with its extended half-life and low clearance, the high PPB of **17c** may contribute to sustained target engagement.

### 3.11. Pharmacokinetic Properties

The Pharmacokinetic (PK) profiles of compound **17c** were evaluated in Sprague Dawley (SD) rats following single-dose administration via the oral (PO) and intravenous (iv) route, and the Animal Care and Use Committee (ACUC) approval documentation was IACUC-ALM-02. As summarized in Table 7, the oral administration of **17c** achieved a peak plasma concentration (Cmax) of 740 ng/mL, at Tmax = 4.0 h, with a half-time (T_1/2_) of 3.46 h and a mean residence time (MRT_inf) of 6.52 h. Compared to lead compound **18c**, compound **17c** demonstrated significantly improved PK properties, including a prolonged oral half-time, reduced clearance, an extended mean residence time, and enhanced oral bioavailability (45.3%). These data indicate that **17c** exhibits favorable PK properties, with sustained drug exposure and optimized absorption characteristics.

## 4. Conclusions

We report a novel series of dual Mer/c-Met inhibitors, with **17c** emerging as the lead candidate. Molecular docking revealed that **17c** forms hydrogen bonds with Mer (Leu593/Lys619) and c-Met (Met1160/Lys1110/Phe1223), complemented by π-π interaction. **17c** exhibited potent enzymatic inhibition and superior antiproliferative activity over cabozantinib against three cancer cell lines. Pharmacokinetically, **17c** showed 45.3% oral bioavailability (16-fold higher than **18c**), an extended half-life (3.46 h), and low hepatic clearance (0.026 mL/min/mg). Safety profiling indicated minimal hERG inhibition. Mechanistically, **17c** induced apoptosis and suppressed HCT116 migration. These data position **17c** as a promising dual Mer/c-Met inhibitor warranting further development.

## Figures and Tables

**Table 1:** Inhibitory activities of compound **13a**–**13k** against Mer and c-Met kinase.

Compd.	R^1^	IC_50_ (nM) ^1^	
		Mer	c-Met
**13a**	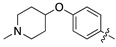	21.7 ± 5.8	63.3 ± 9.1
**13b**	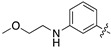	24.3 ± 5.7	25.9 ± 7.7
**13c**	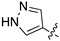	110.1 ± 15.7	48.9 ± 6.4
**13d**	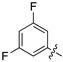	640.2 ± 82.2	728.1 ± 131.4
**13e**	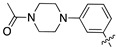	7.5 ± 1.6	7.5 ± 1.1
**13f**	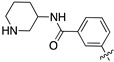	>1000	>1000
**13g**	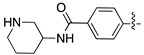	>1000	>1000
**13h**	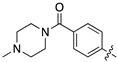	72.0 ± 8.9	41.6 ± 8.5
**13i**	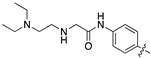	95.3 ± 20.1	88.6 ± 13.9
**13j**	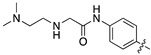	13.1 ± 2.1	48.6 ± 11.2
**13k**	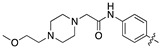	37.3 ± 10.6	35.4 ± 10.0
**18c**	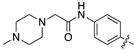	18.5 ± 2.3	33.6 ± 4.3
cabozantinib		0.6 ± 0.1	1.4 ± 0.2

^1^ Data are means from three independent experiments.

**Table 2:** Inhibitory activities of compound **17a**–**17h** against Mer and c-Met kinase.

Compd.	R^2^	IC_50_ (nM) ^1^	
		Mer	c-Met
**17a**	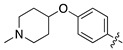	26.2 ± 7.1	233.4 ± 25.4
**17b**	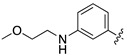	12.1 ± 1.3	100.7 ± 18.5
**17c**	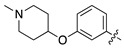	6.4 ± 1.8	26.1 ± 7.7
**17d**	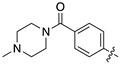	84.1 ± 13.9	137.1 ± 34.3
**17e**	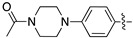	69.8 ± 12.5	235.6 ± 25.6
**17f**	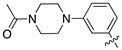	5.5 ± 0.8	25 ± 3.5
**17g**	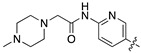	>1000	139.7 ± 18.9
**17h**	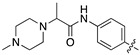	11.9 ± 1.7	45.0 ± 6.8
**18c**	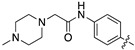	18.5 ± 2.3	33.6 ± 4.3
cabozantinib		0.6 ± 0.1	1.4 ± 0.2

^1^ Data are means from three independent experiments.

**Table 3:** Microsome stability study of compound **13b**, **13e**, **13j**, **13k**, **13m**, **13n**, **17c**, and **17f**.

Cpd.	HUMAN	
	T1/2 (min)	CL (mL/min/mg)
**13b**	38.3	0.0905
**13e**	30.4	0.1142
**13j**	27.9	0.1521
**13k**	38.4	0.1243
**17c**	147.0	0.0236
**17f**	51.0	0.0680
Testosterone	32.1	0.11

**Table 4:** Cytotoxic activities of compound **17c** on cancer cell lines in vitro.

Cpds	IC50 (μM) ^1^ of 3 Cell Lines		
	HepG2	MDA-MB-231	HCT116
**17c**	0.46 ± 0.06	2.31 ± 0.67	3.79 ± 1.09
Cabozantinib	7.87 ± 2.11	4.62 ± 0.54	15.57 ± 4.39

^1^ Data are means from three independent experiments.

**Table 5:** Activity on hERG potassium currents of compound **17c**.

Cpd.	IC50 (μM)
**17c**	>40
Cisapride	0.04

**Table 6:** Stability recovery of compound **17c** and positive control in plasma.

Cpd.	Con. (μM)	Species	Mean Fu	Mean Fb	Stability (%)
**17c**	1	Human	0.0158	98.4%	99.3%
**17c**	1	Rat	0.0178	98.2%	102.2%
Warfarin	1	Human	0.00877	99.1%	99.8%
Warfarin	1	Rat	0.0102	99.0%	98.2%

Fu (Free drug fraction): refers to the portion of the drug in the plasma that is not bound to proteins. Fu = Area ratio of compound in buffer/Area ratio of compound in plasma. Fb (Bound drug fraction): Refers to the portion of the drug that is bound to plasma proteins. Fb = 100 × (1 − Fu).

**Table 7:** Pharmacokinetic parameters of compound **17c**.

Adm.	Dose (mg/kg)	AUC_last_ (h*ng/mL)	T_1/2_ (h)	T_max_ (h)	C_max_ (ng/mL)	CL__obs_ (mL/min/kg)	MRTINF__obs_ (h)	F (%)
p.o.	10	4626	3.46	4.00	740	–	6.52	45.3
i.v.	2	2154	3.33	–	–	16.4	3.23	

## Data Availability

The original contributions presented in this study are included in the article/Supplementary Material. Further inquiries can be directed to the corresponding authors.

## Supplementary Materials

The following supporting information can be downloaded at: https://www.mdpi.com/article/10.3390/biom15081180/s1, S1: Supporting Tables; S2: Raw materials of biology; S3: Mer inhibitory results; S4: c-Met inhibitory results; S5: Antiproliferative activities against cancer cell lines; S6–S81: ^1^H-NMR, ^13^C-NMR, HRMS, and HPLC of target compounds; S82: A quantification and a fold change analysis of western blot analysis; S83: Re-docking files; S84: Alignment of 17c and foretinib; S85: Original Western blot images.
