# Supplementary material for: Discovery of Novel 2-Substituted Aniline Pyrimidine Based Derivatives as Potent Mer/c-Met Dual Inhibitors with Improvement Bioavailability
Source: Biomolecules. 2025 Aug 18;15(8):1180. doi: 10.3390/biom15081180 (PMC12385147; doi:10.3390/biom15081180)

Discovery of Novel 2-Substituted Aniline Pyrimidine Based Derivatives as Potent Mer/c-Met dual inhibitors with Improvement Bioavailability

Jixia Yang,[1] Daowei Huang,[2]* Ruojin Wang,[1] Pengxin Fan,[1] Rourou Li,[1] Donglai Ma[1]*

[1]*School of Pharmacy, Hebei University of Chinese Medicine, Shijiazhuang, 050200, China*

[2]*School of Chemical and Pharmaceutical Engineering, Hebei University of Science and Technology, Shijiazhuang, 050018, China*

*Corresponding author: E-mail: huangdaowei321@163.com, mdl_hebei@aliyun.com

Contents

1. Supporting Tables …..……..… ..… ..… ..… ..… ..… S1
2. Raw materials of biology…..……..… ..… ..… ..… … S2
3. Mer inhibitory results…..……..… ..… …. ..… S3
4. c-Met inhibitory results…..……..… ..… ..… ..… ..… S4
5. Antiproliferative activities against cancer cell lines….…….S5
6. ^{1}H-NMR, ^{13}C-NMR, HRMS, and HPLC of target compounds

^{1}H-NMR, ^{13}C-NMR, HRMS, and HPLC of compound **13a** S6-S9
^{1}H-NMR, ^{13}C-NMR, HRMS, and HPLC of compound **13b** S10-S13
^{1}H-NMR, ^{13}C-NMR, HRMS, and HPLC of compound **13c** S14-S17
^{1}H-NMR, ^{13}C-NMR, HRMS, and HPLC of compound **13d** S18-S21
^{1}H-NMR, ^{13}C-NMR, HRMS, and HPLC of compound **13e** S22-S25
^{1}H-NMR, ^{13}C-NMR, HRMS, and HPLC of compound **13f** S26-S29
^{1}H-NMR, ^{13}C-NMR, HRMS, and HPLC of compound **13g** S30-S33
^{1}H-NMR, ^{13}C-NMR, HRMS, and HPLC of compound **13h** S34-S37
^{1}H-NMR, ^{13}C-NMR, HRMS, and HPLC of compound **13i** S38-S41
^{1}H-NMR, ^{13}C-NMR, HRMS, and HPLC of compound **13j** S42-S45
^{1}H-NMR, ^{13}C-NMR, HRMS, and HPLC of compound **13k** S46-S49
^{1}H-NMR, ^{13}C-NMR, HRMS, and HPLC of compound **17a** S50-S53

^{1}H-NMR, ^{13}C-NMR, HRMS, and HPLC of compound **17b** S54-S57
^{1}H-NMR, ^{13}C-NMR, HRMS, and HPLC of compound **17c** S58-S61
^{1}H-NMR, ^{13}C-NMR, HRMS, and HPLC of compound **17d** S62-S65
^{1}H-NMR, ^{13}C-NMR, HRMS, and HPLC of compound **17e** S66-S69
^{1}H-NMR, ^{13}C-NMR, HRMS, and HPLC of compound **17f** S70-S73
^{1}H-NMR, ^{13}C-NMR, HRMS, and HPLC of compound **17g** S74-S77
^{1}H-NMR, ^{13}C-NMR, HRMS, and HPLC of compound **17h** S78-S81

7. A quantification and a fold change analysis of western blot analysis S82
8. Re-docking files S83
9. Alignment of 17c and foretinib S84
10. Original Western blot images S85

S1: Chemical structures of all target compounds

Cpd.	Structure	Cpd.	Structure
**13a**	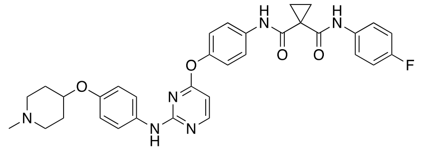	**13k**	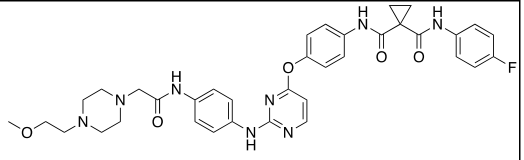
**13b**	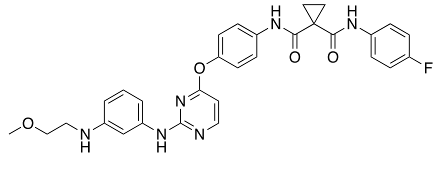	**17a**	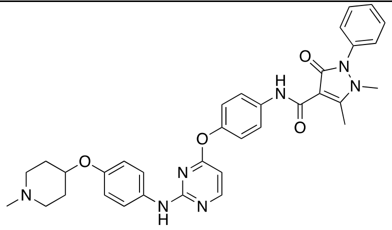
**13c**	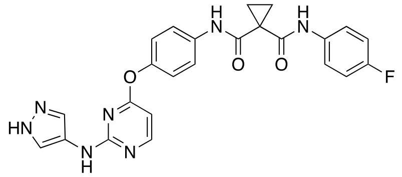	**17b**	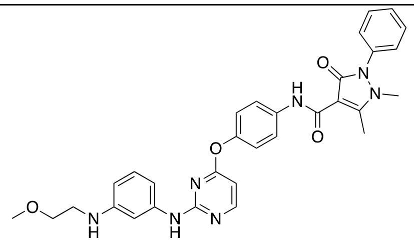
**13d**	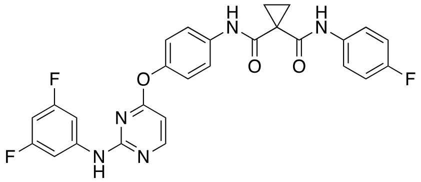	**17c**	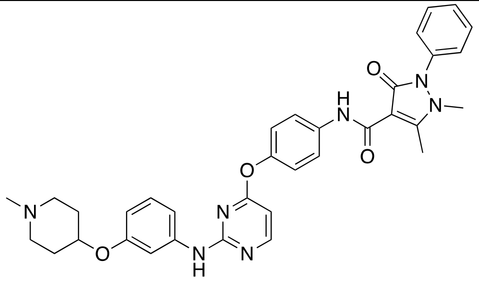
**13e**	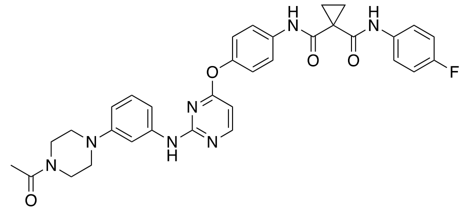	**17d**	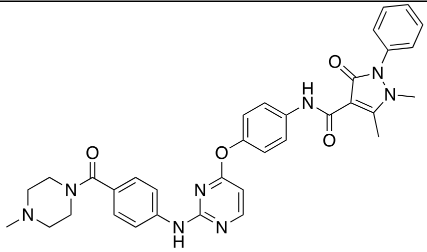
**13f**	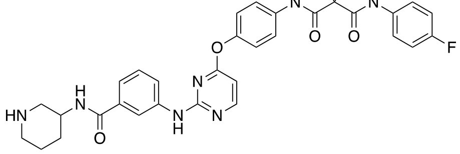	**17e**	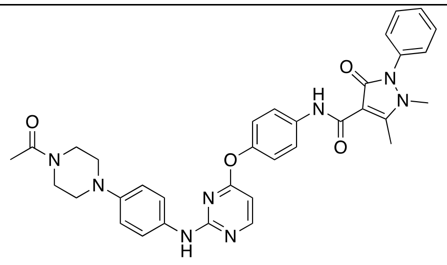

**13g**	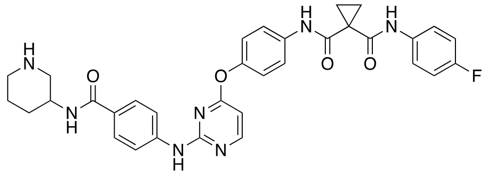	**17f**	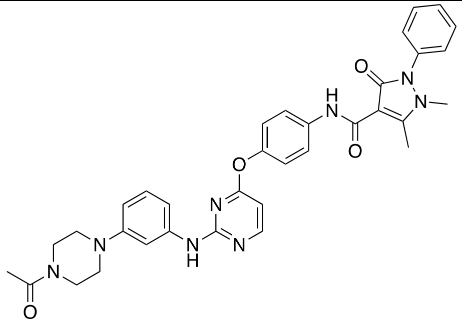
**13h**	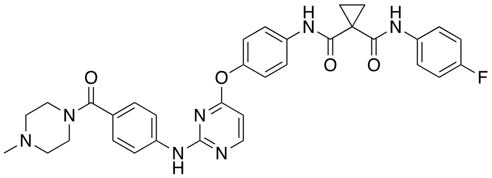	**17g**	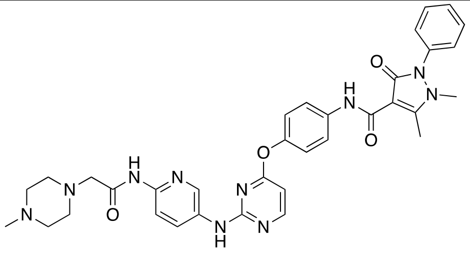
**13i**	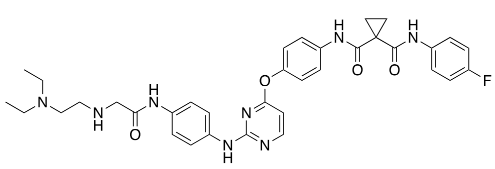	**17h**	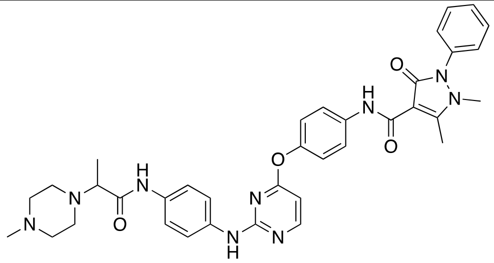
**13j**	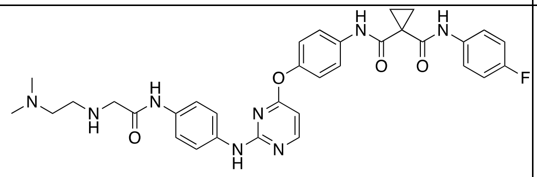		

S2: Raw materials of biology

Mer and c-Met kinase kit were purchased from Invitrogen (Shanghai, China). ATP was obtained from Promega Co., Ltd (Shanghai, China). Phosphate buffer saline (PBS), DMEM-high glucose medium, and fetal bovine serum (FBS) were purchased from Sigma (Louis, MO, USA). $MgCl_2$, penicillin/streptomycin were purchased from Dalian Meilun Biotechnology Co., Ltd (Dalian, China). Nicotinamide adenine dinucleotide phosphate (NADPH) was obtained from Shanghai yuanye Bio-Technology Co., Ltd (Shanghai, China). Liver microsomes, Hank's balanced salt solution (HBSS), and HEPES was obtained from Corning Inc. (New York, NY, USA). None-essential amino acids were from Gibco (Waltham, MA, USA). CCK8 kit was obtained from Bergolin Biotechnology Co., Ltd (Dalian, China). One Step TUNEL Apoptosis Detection Kit (FITC) was obtained from Meilun Bergolin Biotechnology Co., Ltd (Dalian, China). Rat plasma as blank matrix was purchased from Shanghai Chengze Biotechnology (Shanghai, China).

Phosphate buffer saline (PBS), F12 medium, antibiotics and fetal bovine serum (FBS) were purchased from Invitrogen Trading (Shanghai) Co.,Ltd. All other reagents including reference compound cisapride were purchased from Sigma-Aldrich China Inc.

Name: Phospho-MER/TYRO3 (Tyr753/Tyr685) antibody, Species: Rabit, Sequence: synthesized peptide derived from human MER/TYRO3 around the Phosphorylation site of Tyr753/Tyr685, Dilution: 1:2000, RRID number: AB_2840500, Catalogue number: AF8443, Supplier: Affinity Biosciences.

Name: Phospho-c-Met (Tyr1234) antibody, Species: Rabit, Sequence: synthesized peptide derived from human c-Met around the Phosphorylation site of Tyr1234, Dilution: 1:2000, RRID number: AB_2834564, Catalogue number: AF3129, Supplier: Affinity Biosciences.

S3: Mer inhibitory results

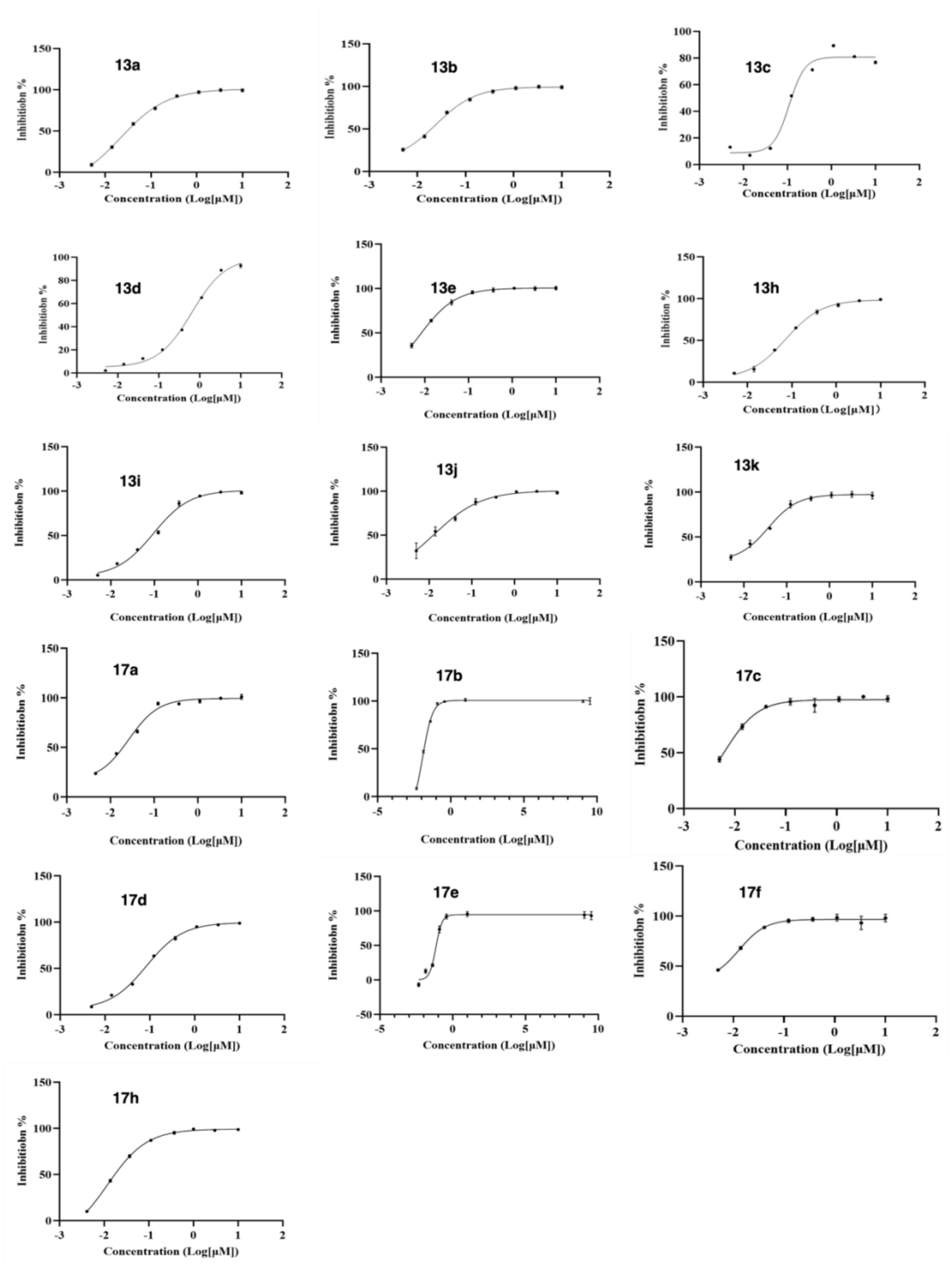

S4: c-Met inhibitory results

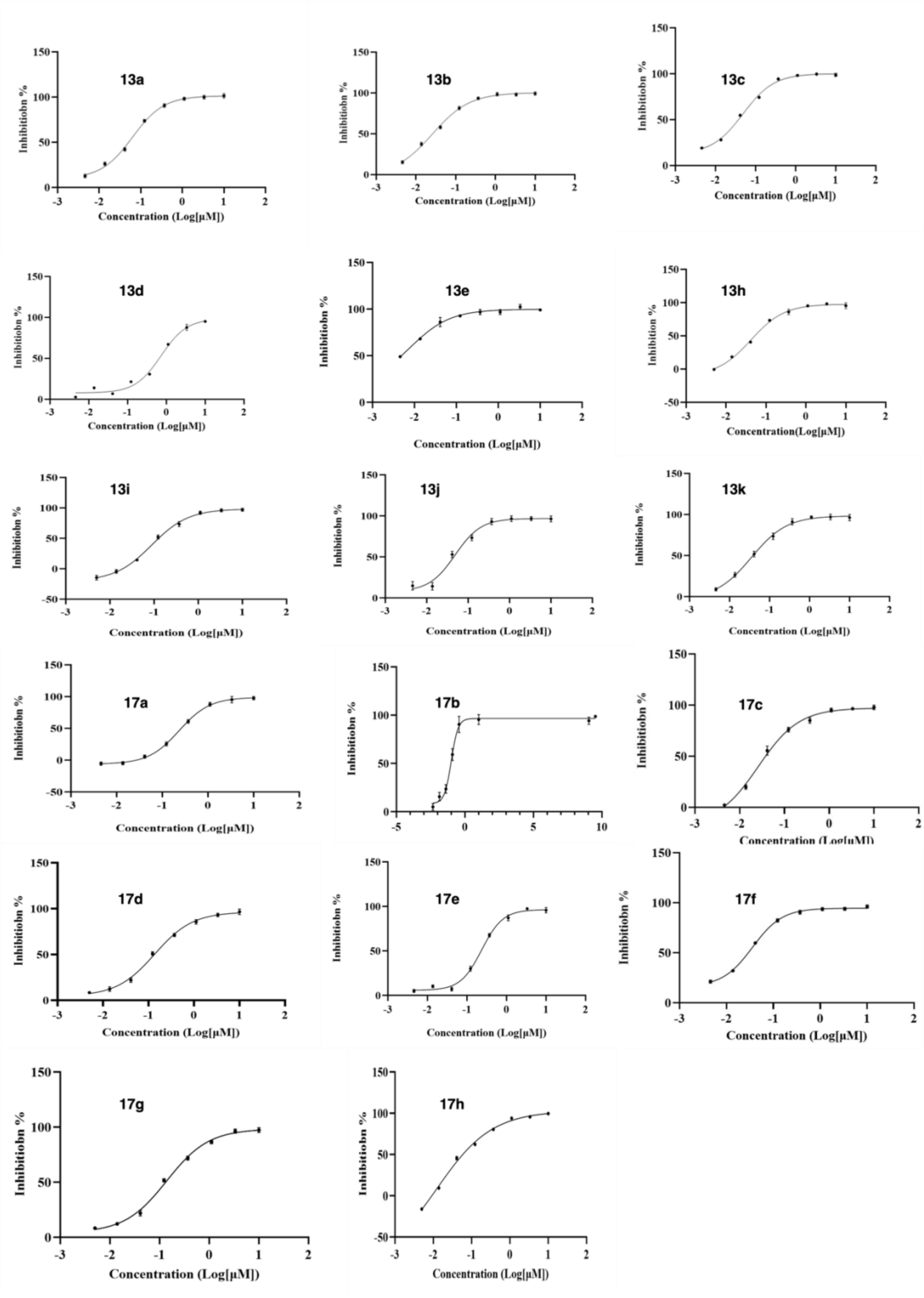

S5: Antiproliferative activities against cancer cell lines

HCT116

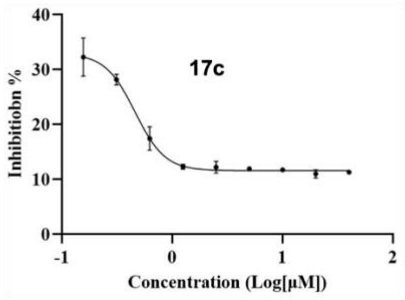

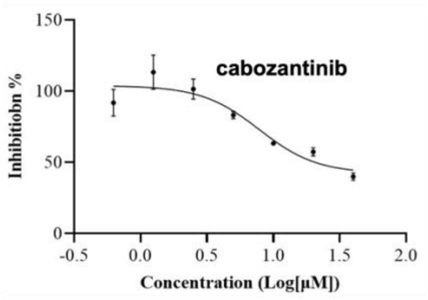

CAKI-1

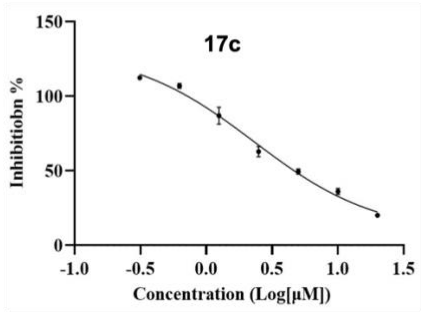

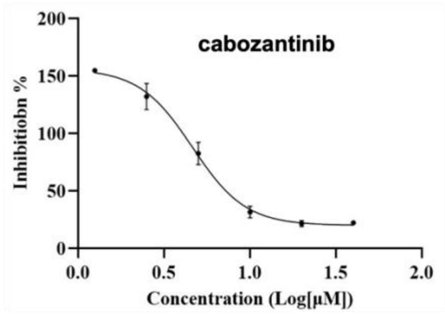

PC-3

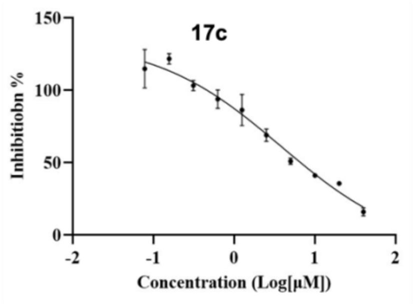

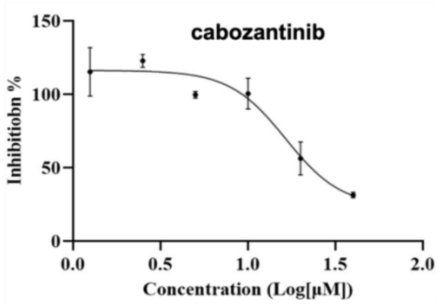

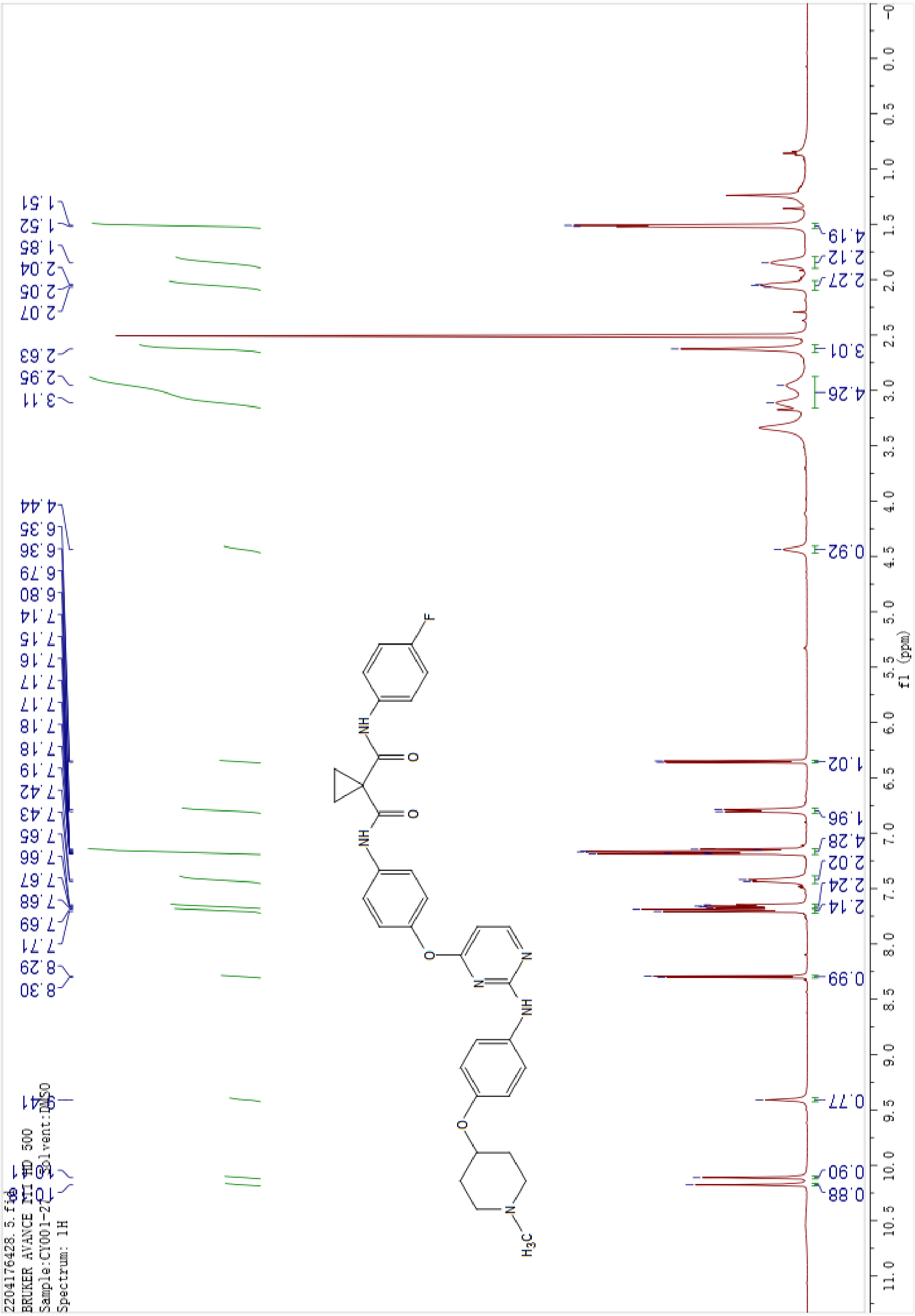

S6: ^{1}H NMR of **13a**

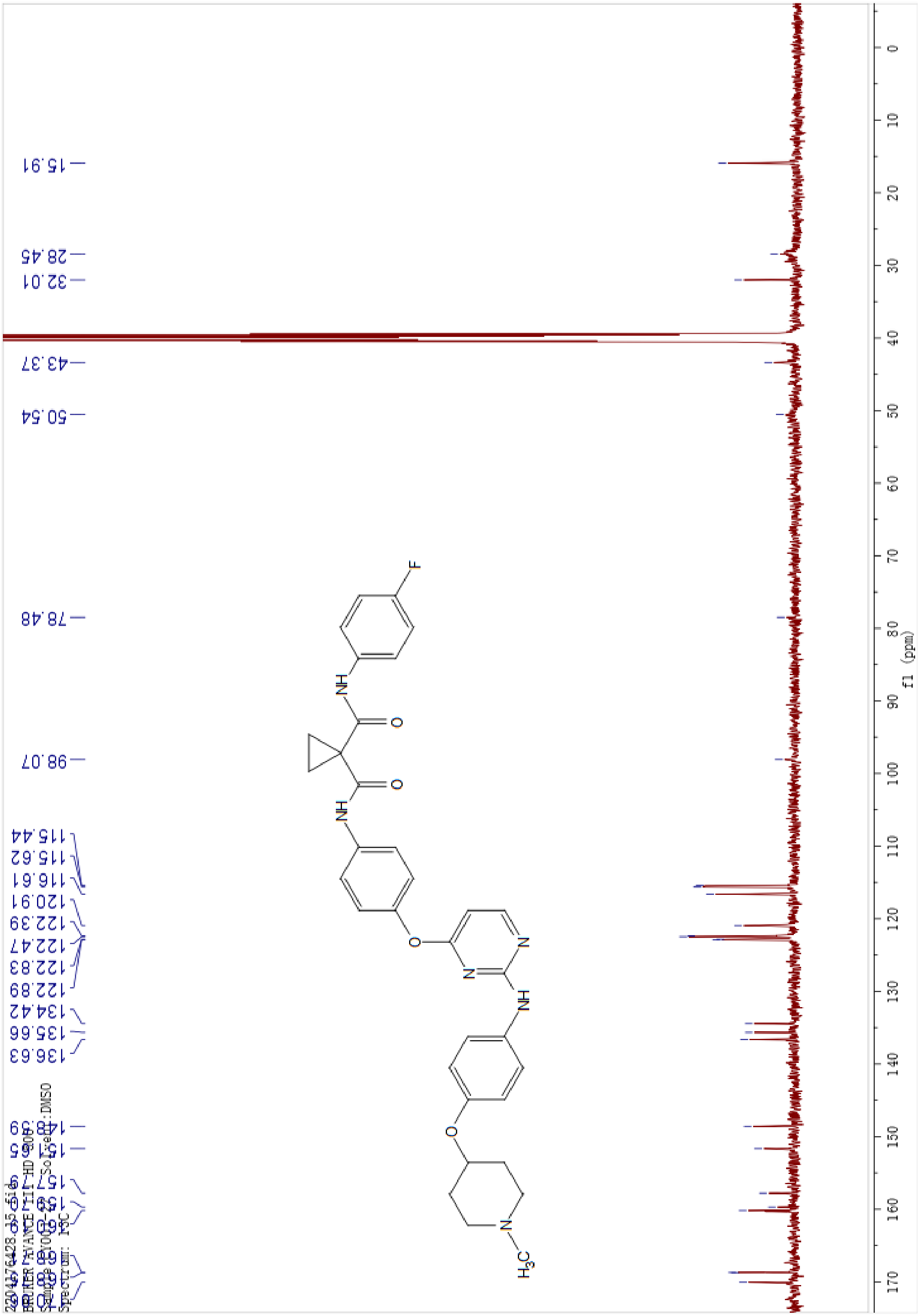

S7: ^{13}C-NMR of **13a**

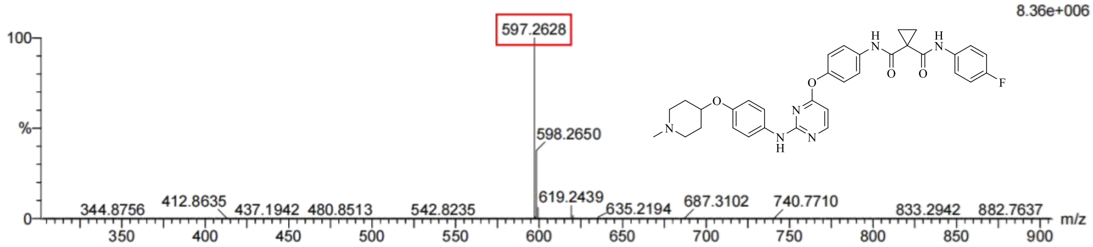

S8: HRMS of **13a**

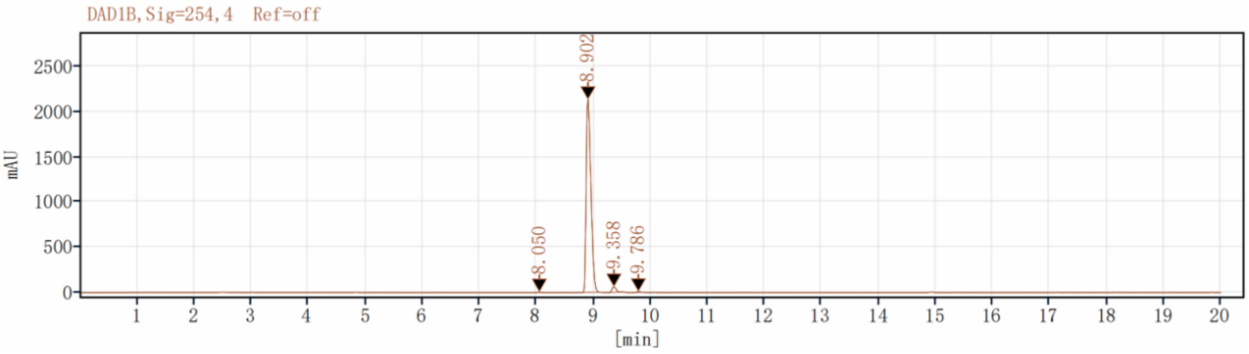

	Retention time(Min)	Peak width(Min)	Area (μV*s)	Height (μV)	% Area
1	8.050	0.11	18.79	5.68	0.15
2	8.902	0.31	12420.70	2140.94	97.24
3	9.358	0.21	297.67	60.75	2.33
4	9.786	0.13	36.07	8.82	0.28

S9: HPLC of **13a**

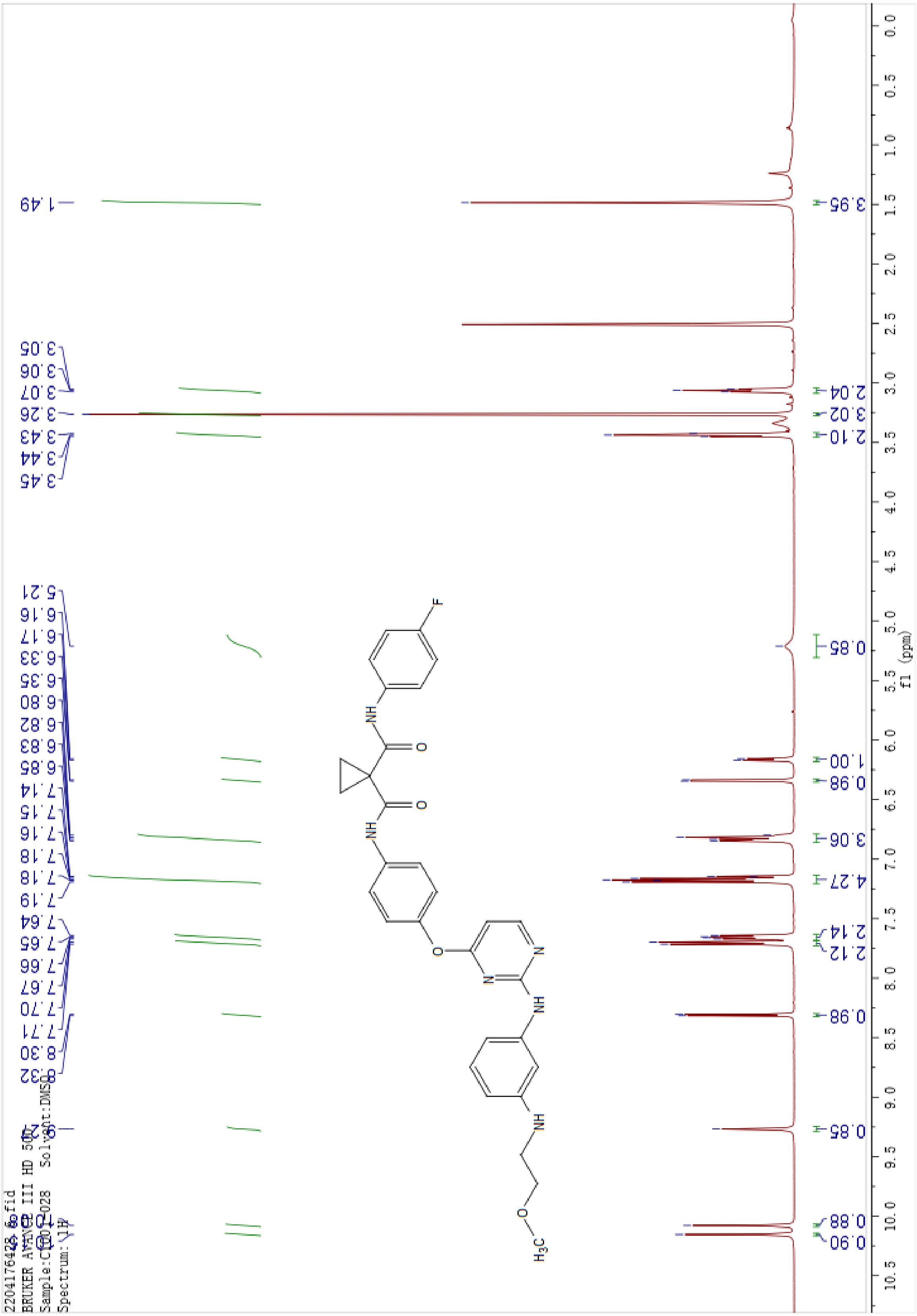

S$_{10}$: ^{1}H NMR of **13b**

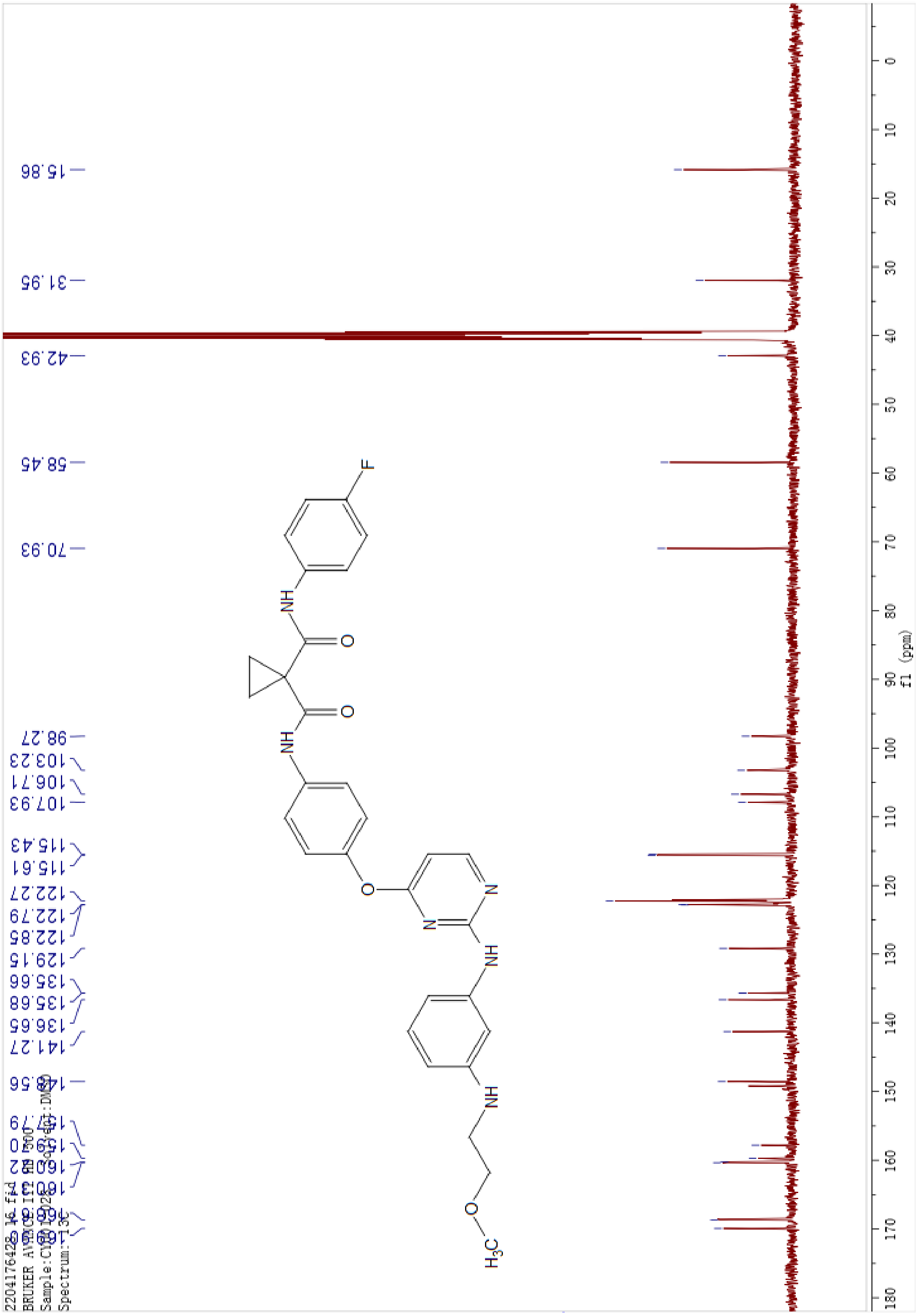

S11: ^{13}C-NMR of **13b**

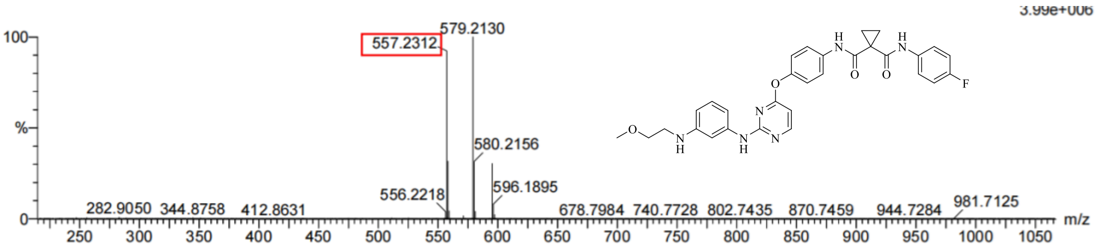

S12: HRMS of **13b**

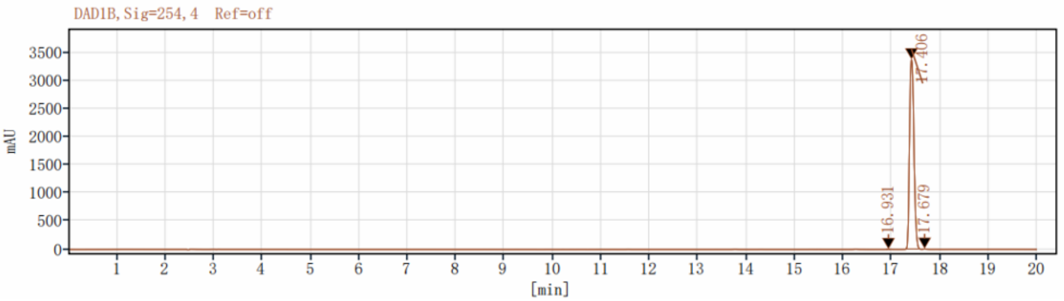

	Retention time (Min)	Peak width (Min)	Area (μV*s)	Height (μV)	% Area
1	16.931	0.08	5.69	1.92	0.03
2	17.406	0.28	21426.40	3382.75	99.90
3	17.679	0.09	15.14	4.92	0.07

S13: HPLC of **13b**

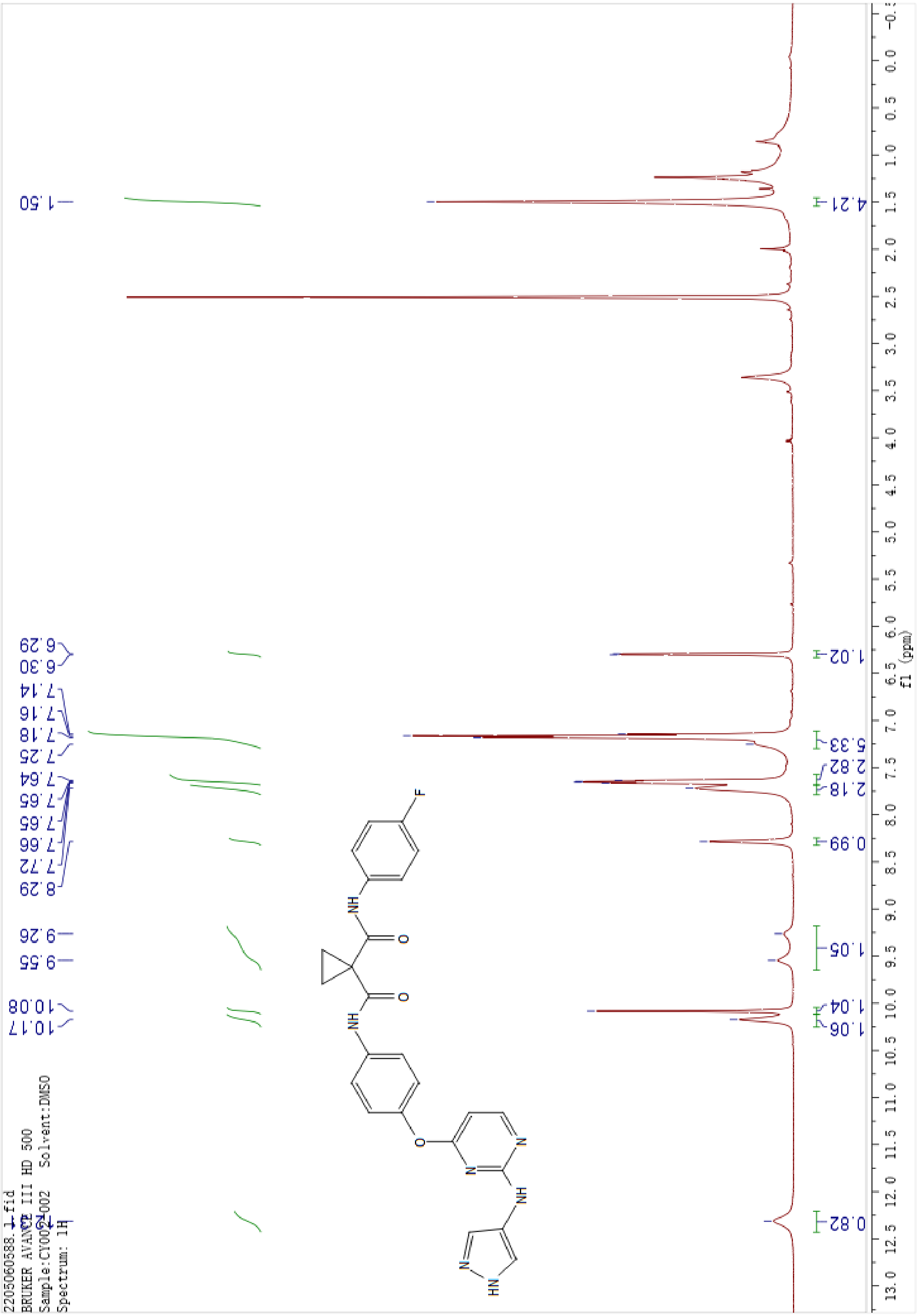

S14: ^{1}H NMR of **13c**

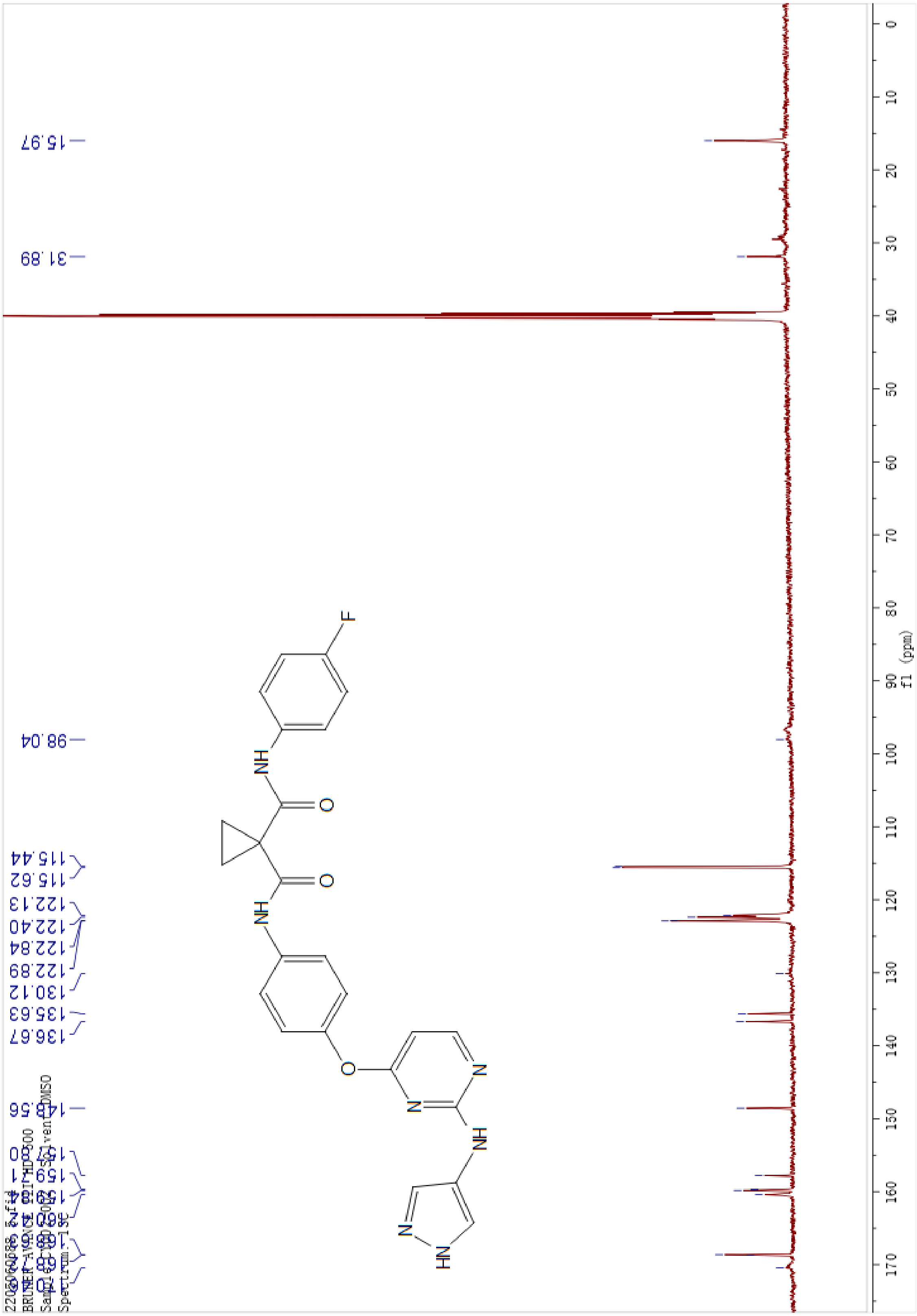

S15: ^{13}C-NMR of **13c**

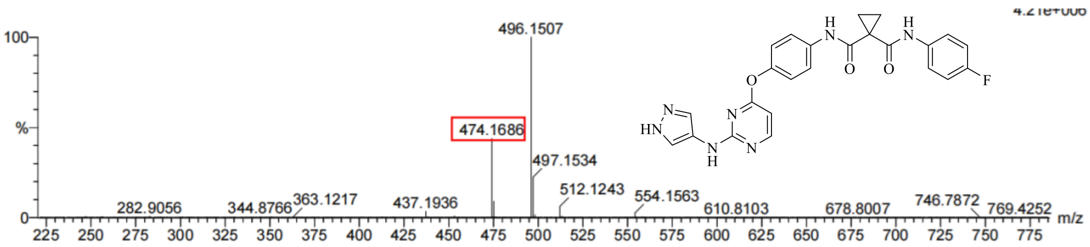

S16: HRMS of **13c**

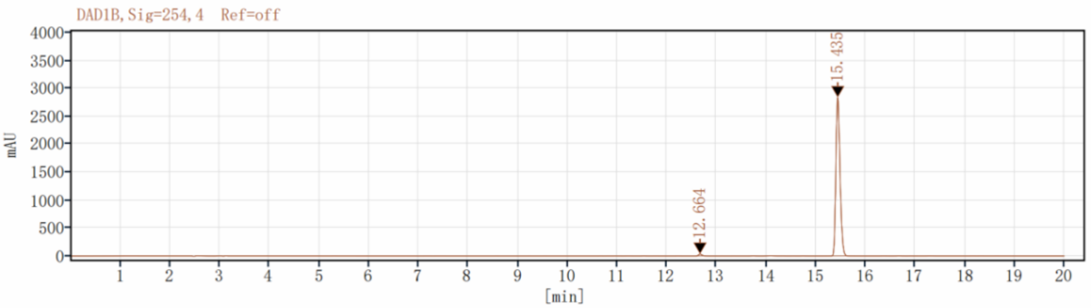

	Retention time (Min)	Peak width (Min)	Area (μV*s)	Height (μV)	% Area
1	12.664	0.17	154.69	33.20	0.93
2	15.435	0.31	16466.54	2843.04	99.07

S17: HPLC of **13c**

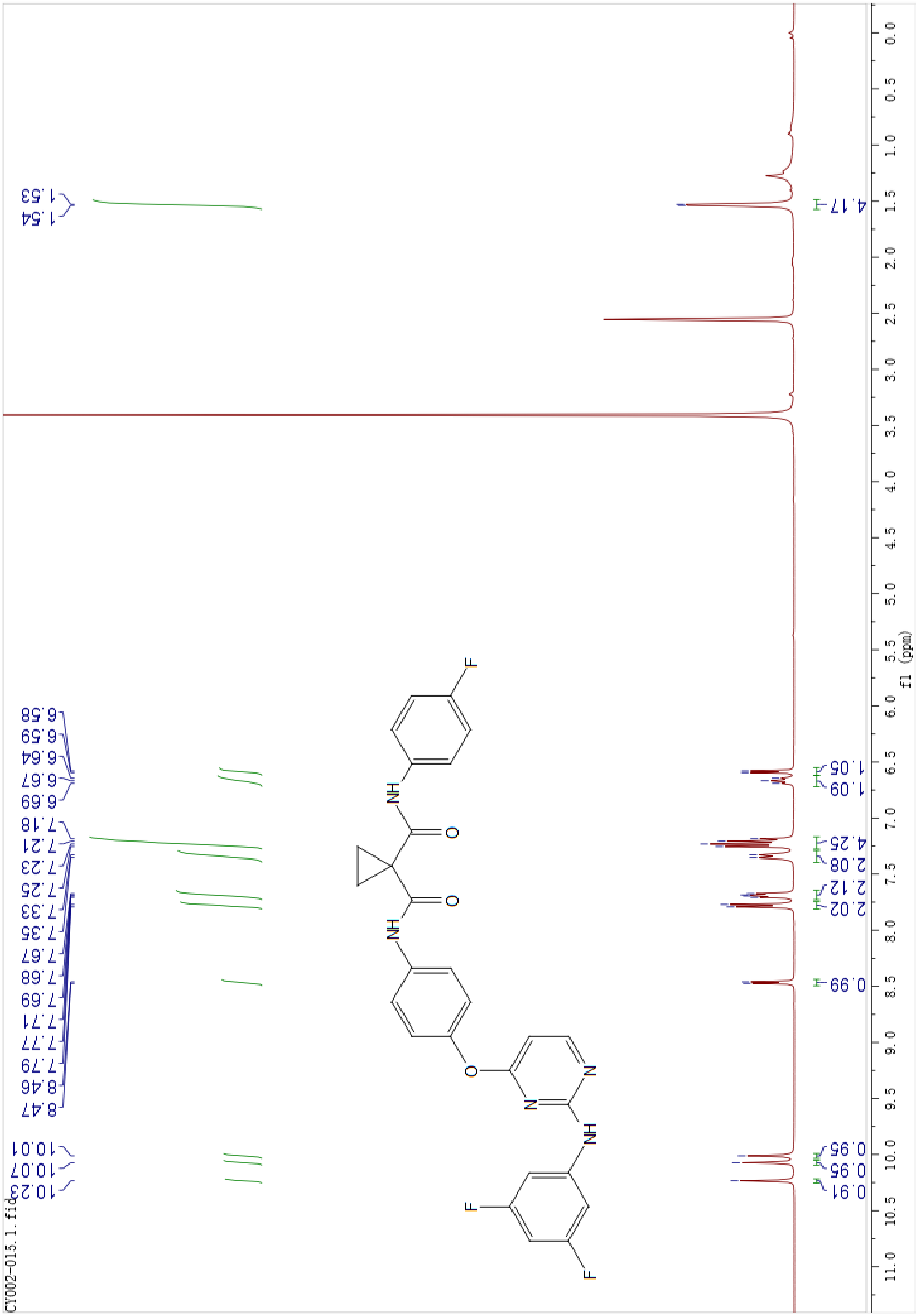

S18: ^{1}H-NMR of **13d**

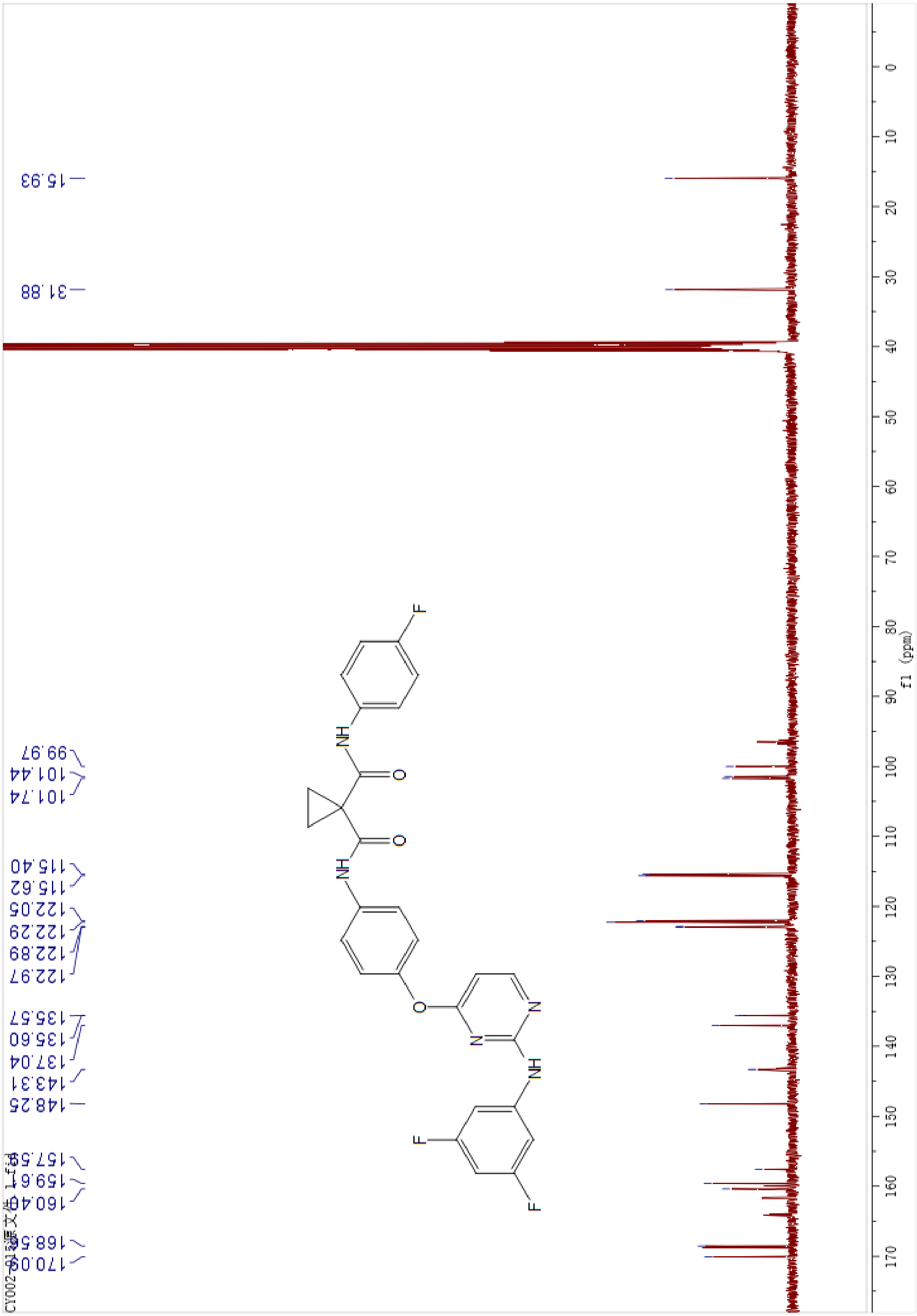

S19: ^{13}C-NMR of **13d**

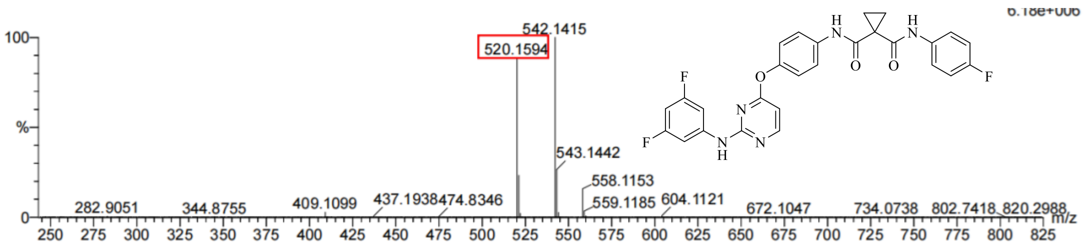

S20: HRMS of **13d**

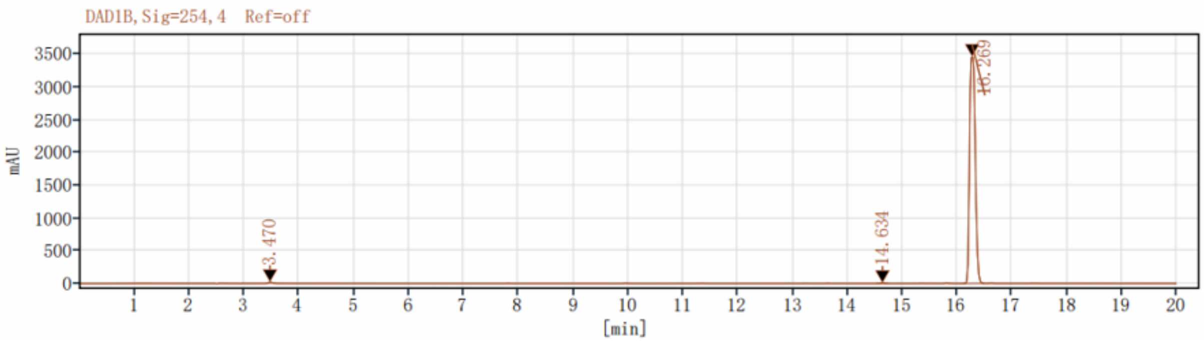

	Retention time (Min)	Peak width (Min)	Area (μV*s)	Height (μV)	% Area
1	3.470	0.14	72.93	15.93	0.29
2	14.634	0.22	34.95	7.27	0.14
3	16.269	0.39	24915.38	3470.60	99.57

S21: HPLC of **13d**

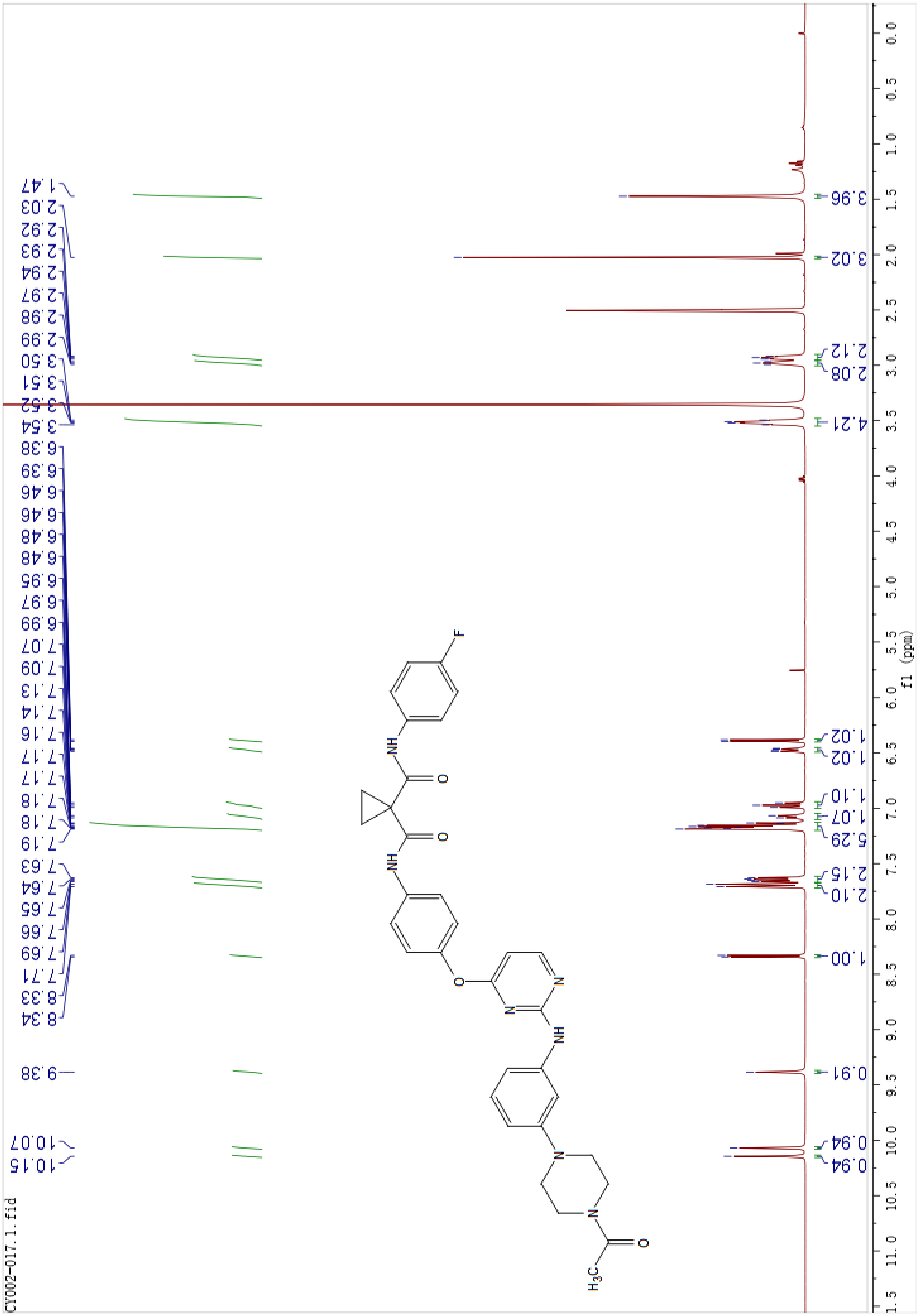

S22: ^{1}H-NMR of **13e**

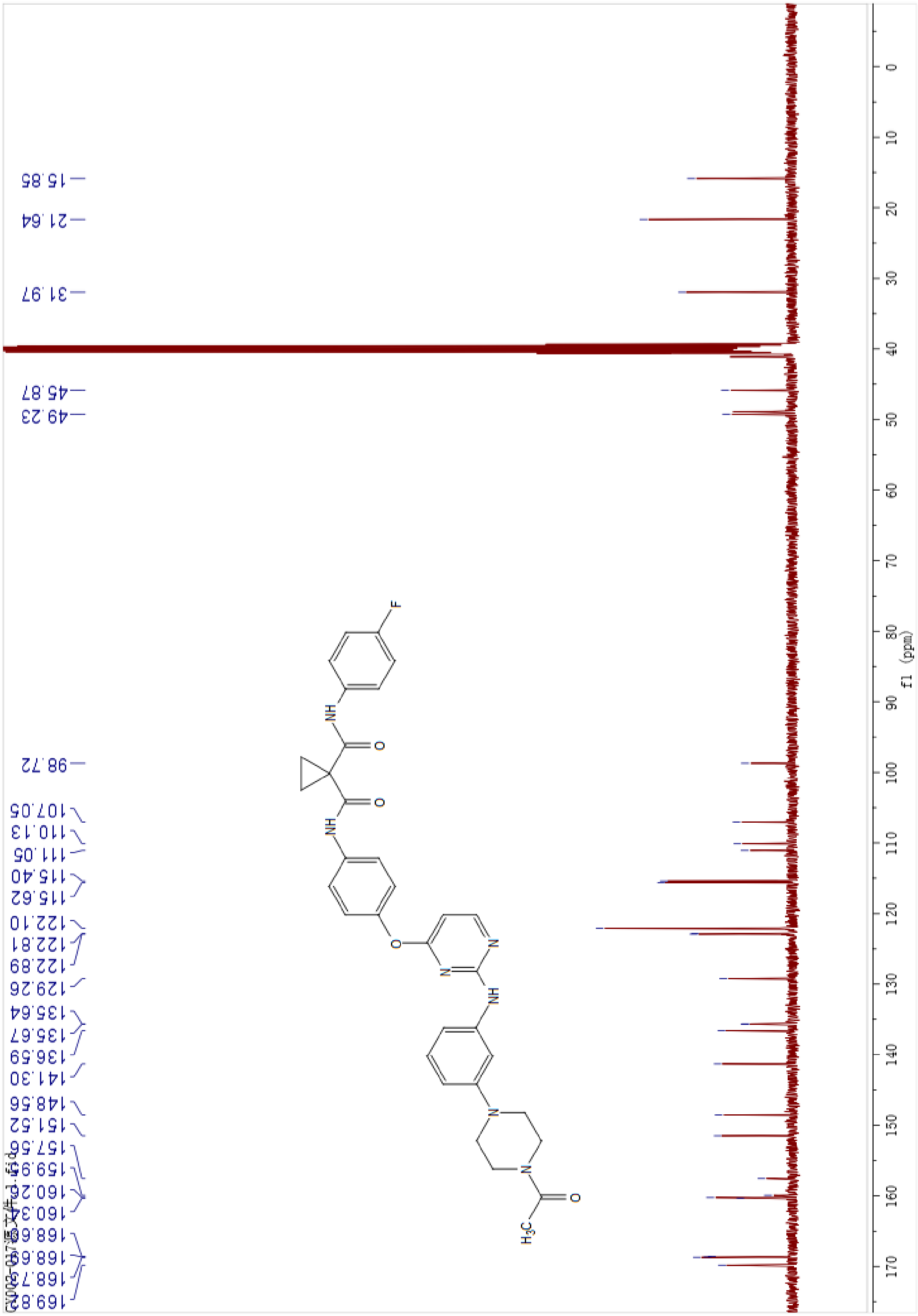

S23: ^{13}C-NMR of **13e**

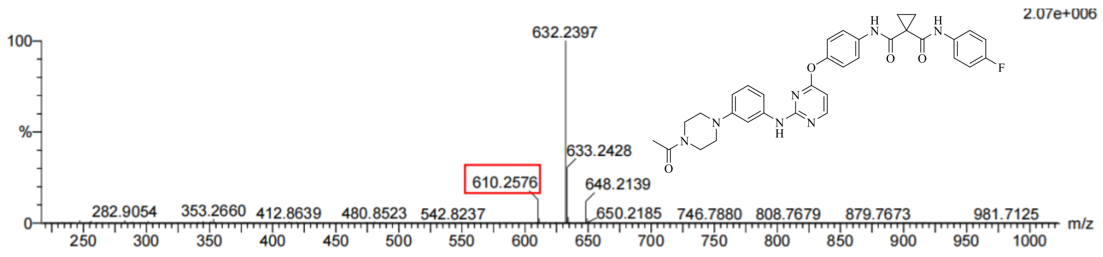

S24: HRMS of **13e**

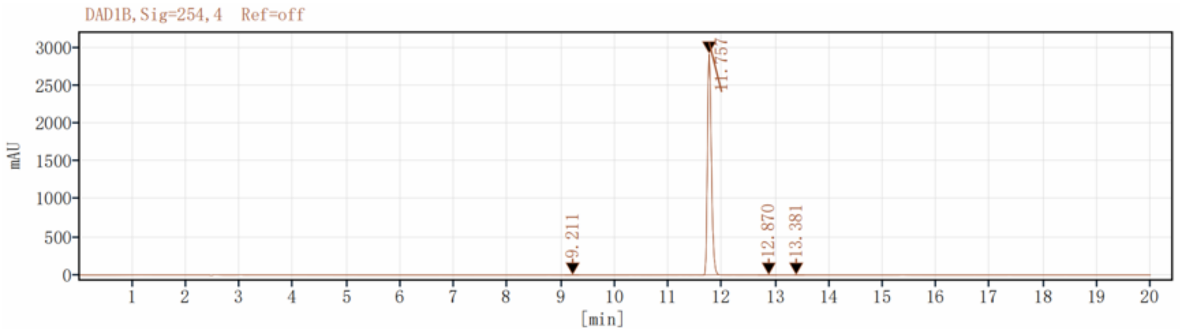

	Retention time (Min)	Peak width (Min)	Area (μV*s)	Height (μV)	% Area
1	9.211	0.27	6.62	1.26	0.04
2	11.757	0.31	15033.38	2925.70	99.92
3	12.870	0.36	3.94	0.64	0.03
4	13.381	0.20	1.16	0.29	0.01

S25: HPLC of **13e**

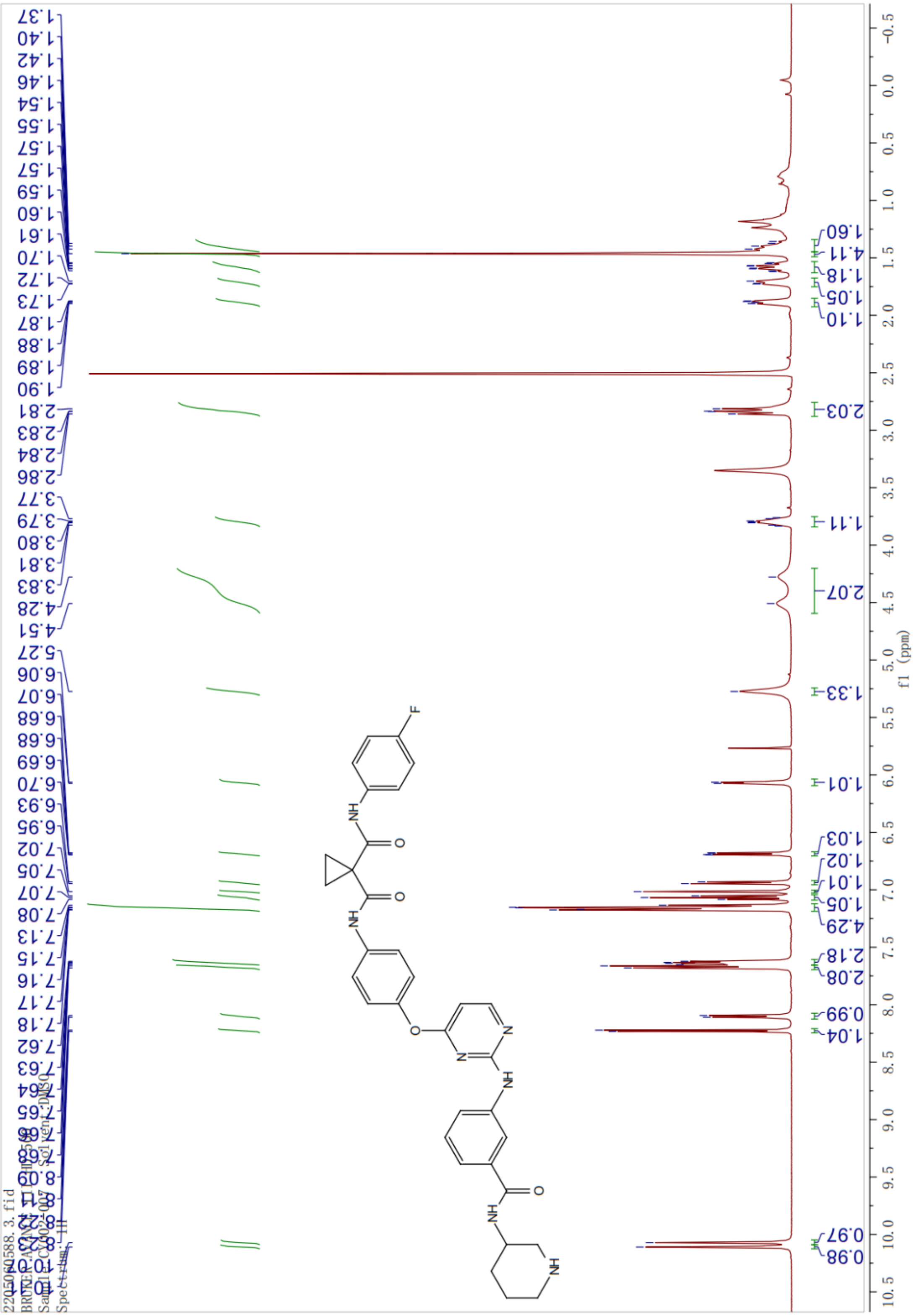

S26: ^{1}H-NMR of **13f**

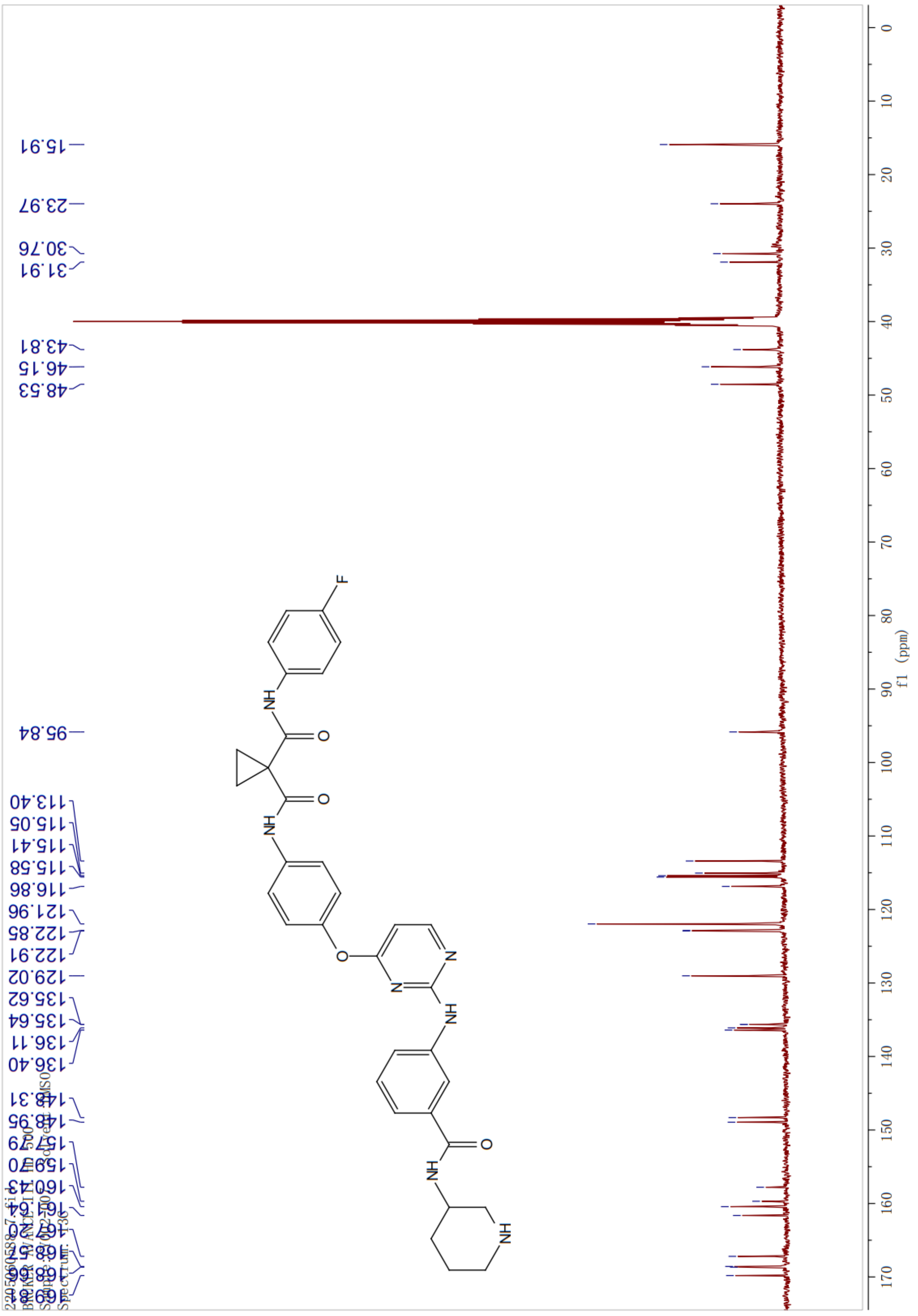

S27: ^{13}C-NMR of **13f**

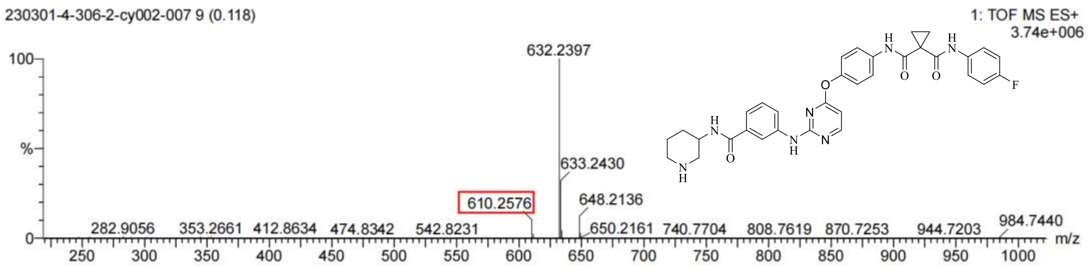

S28: HRMS of **13f**

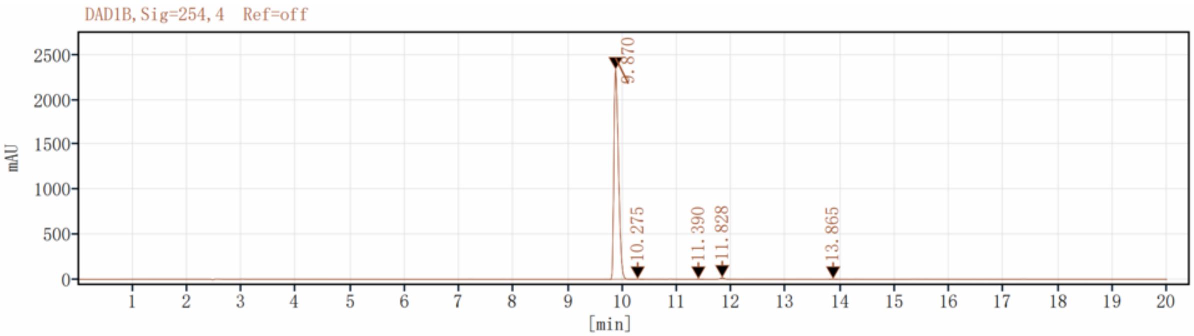

	Retention time(Min)	Peak width(Min)	Area (μV*s)	Height (μV)	% Area
1	9.870	0.36	13629.48	2341.39	99.38
2	10.275	0.27	8.18	2.46	0.06
3	11.390	0.14	6.49	1.92	0.05
4	11.828	0.17	58.56	14.50	0.43
5	13.865	0.17	12.13	2.69	0.09

S29: HPLC of **13f**

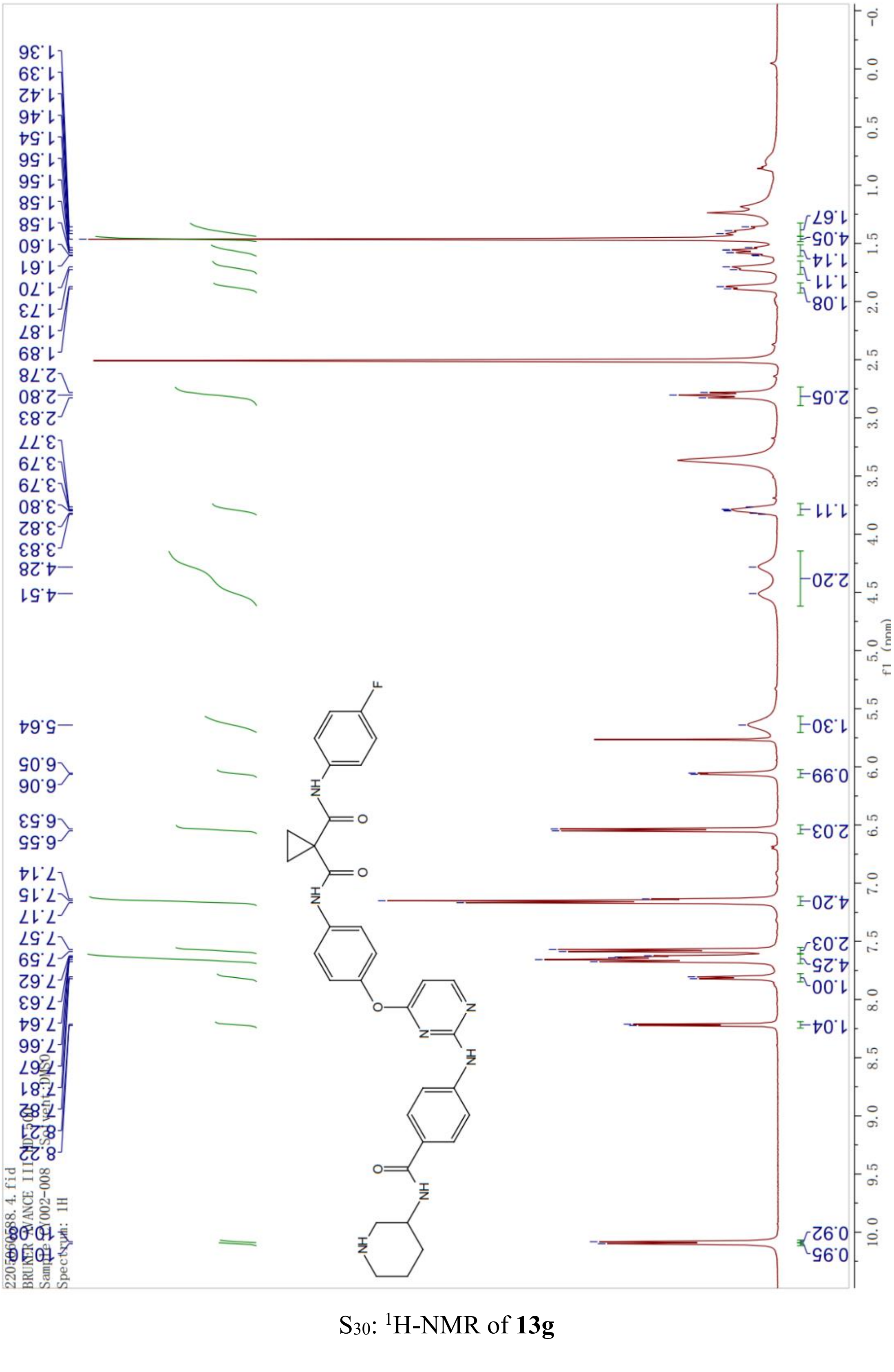

S30: ^{1}H-NMR of **13g**

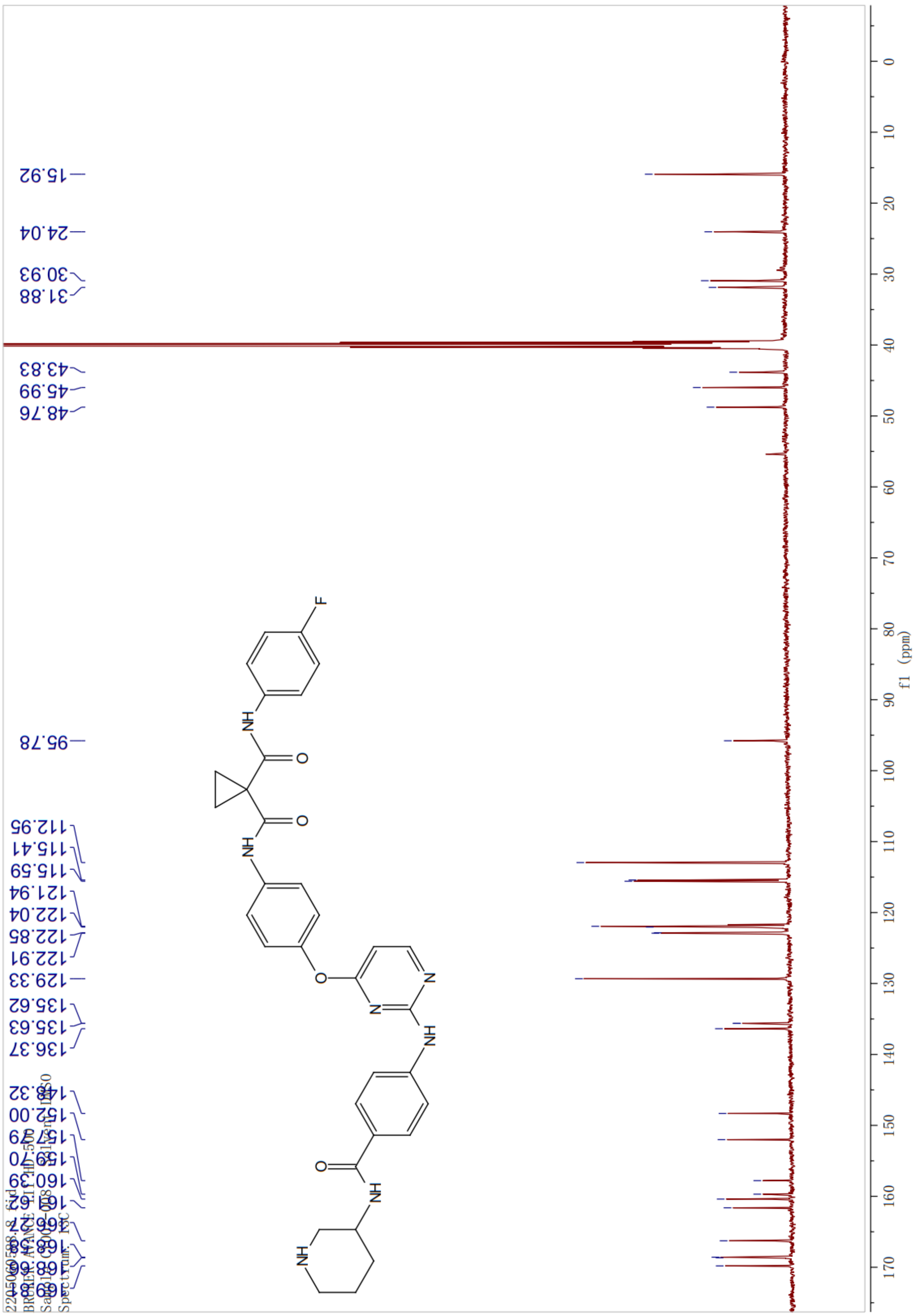

S$_{31}$: ^{13}C-NMR of **13g**

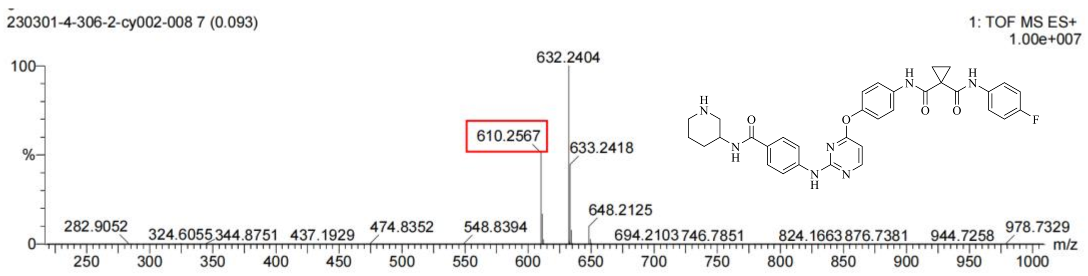

S32: MS of **13g**

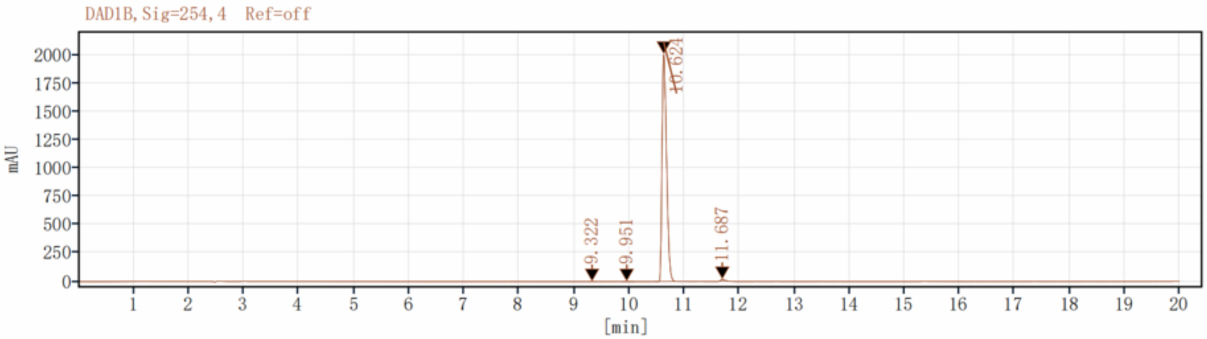

	Retention time (Min)	Peak width (Min)	Area (μV*s)	Height (μV)	% Area
1	9.322	0.11	3.53	0.95	0.03
2	9.951	0.21	6.34	1.11	0.05
3	10.624	0.40	11470.14	2008.80	99.33
4	11.687	0.14	67.38	15.83	0.58

S33: HPLC of **13g**

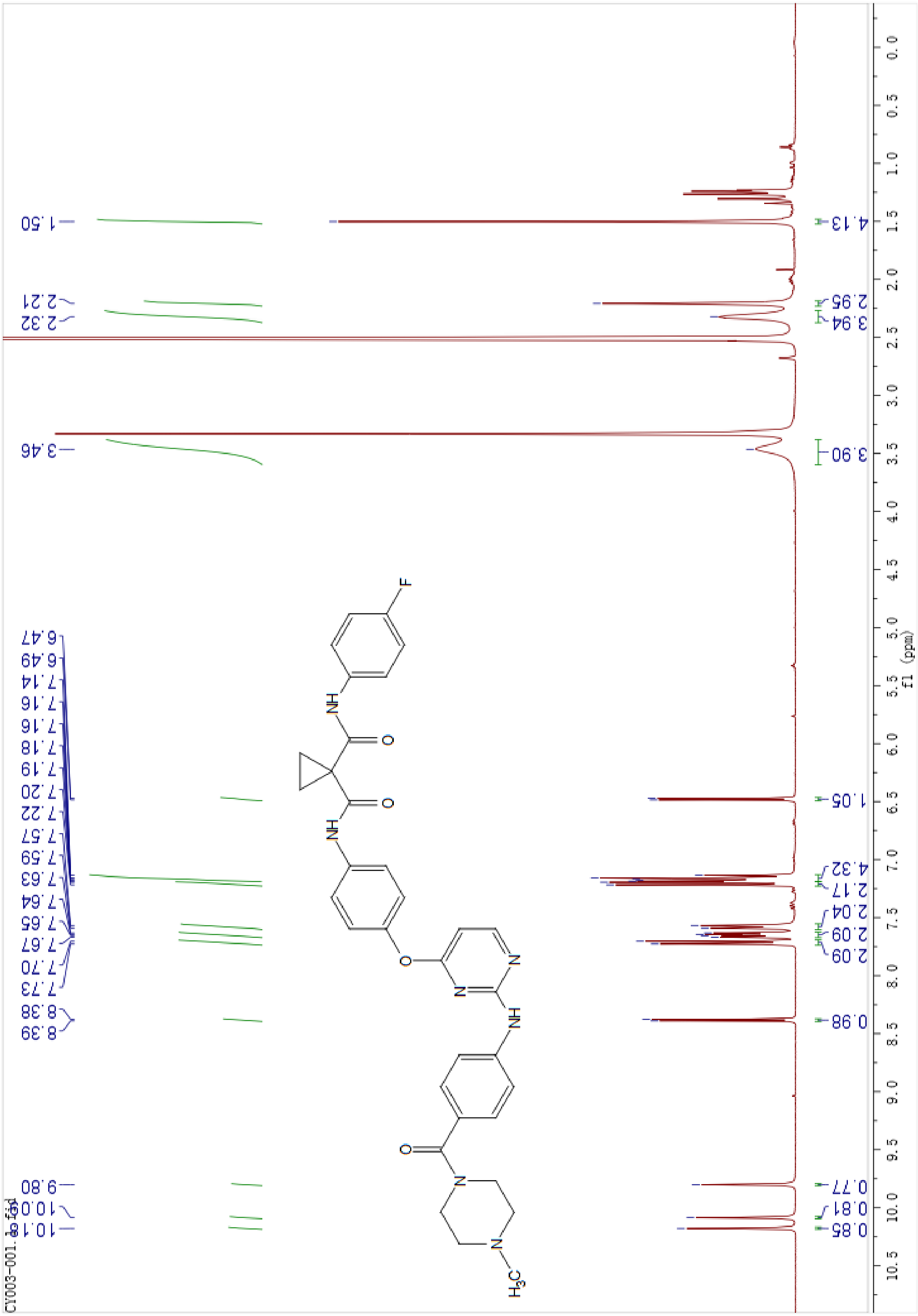

S34: ^{1}H NMR of **13h**

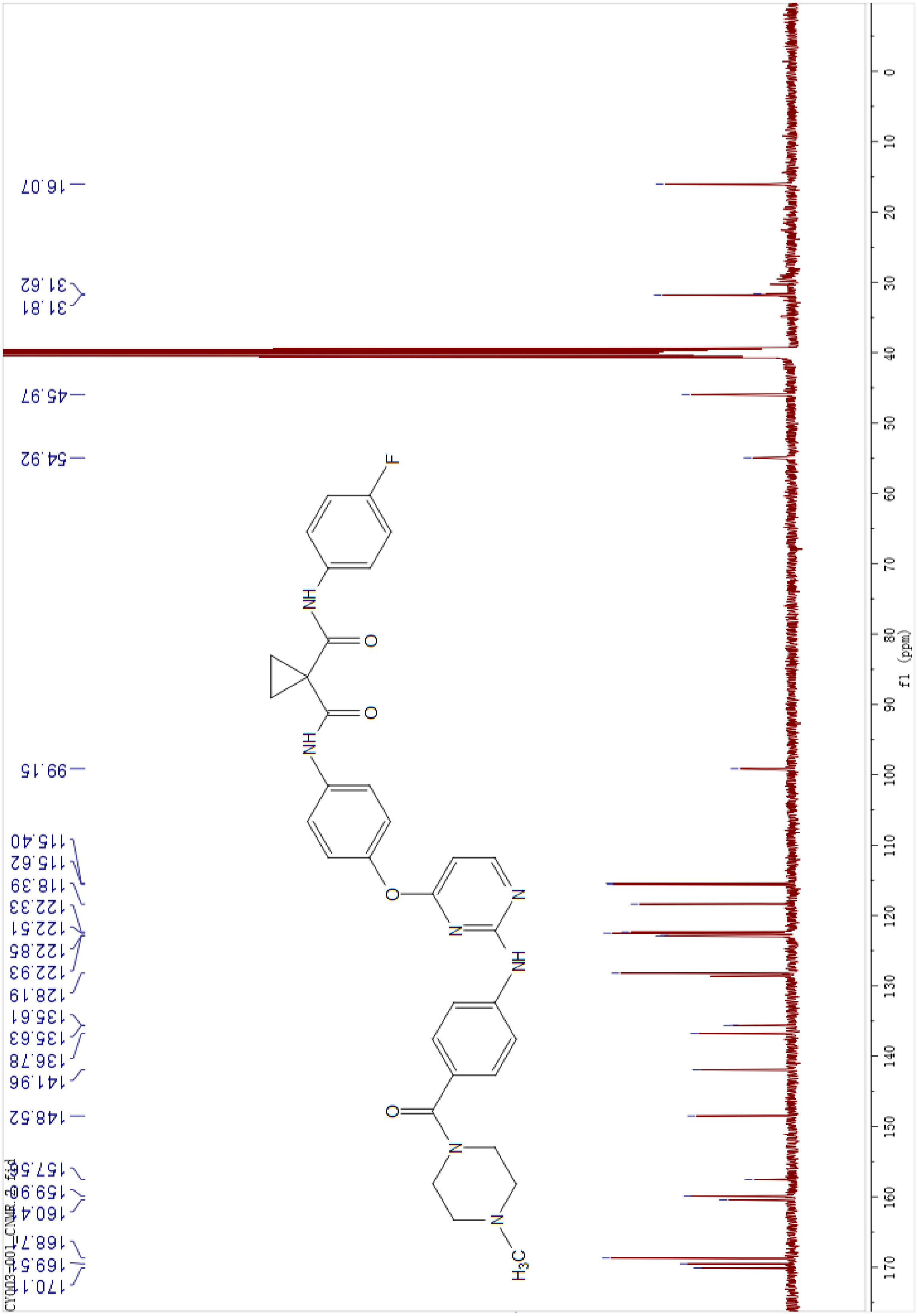

S35: ^{13}C-NMR of **13h**

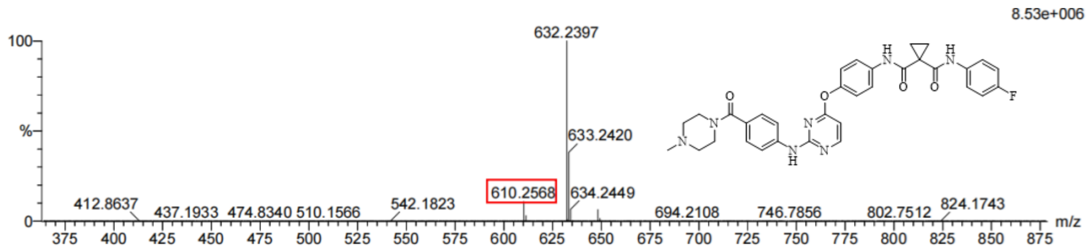

S36: HRMS of **13h**

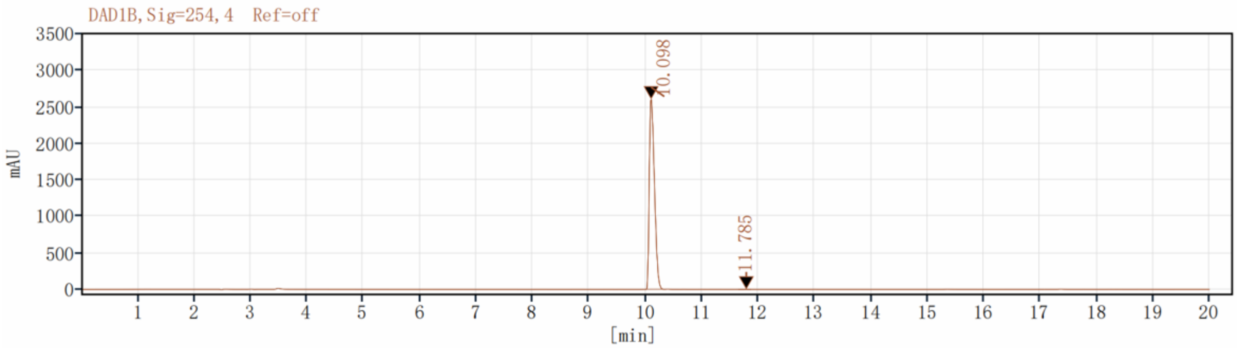

	Retention time (Min)	Peak width (Min)	Area (μV*s)	Height (μV)	% Area
1	10.098	0.31	16694.23	2613.95	99.97
2	11.785	0.22	5.62	1.16	0.03

S37: HPLC of **13h**

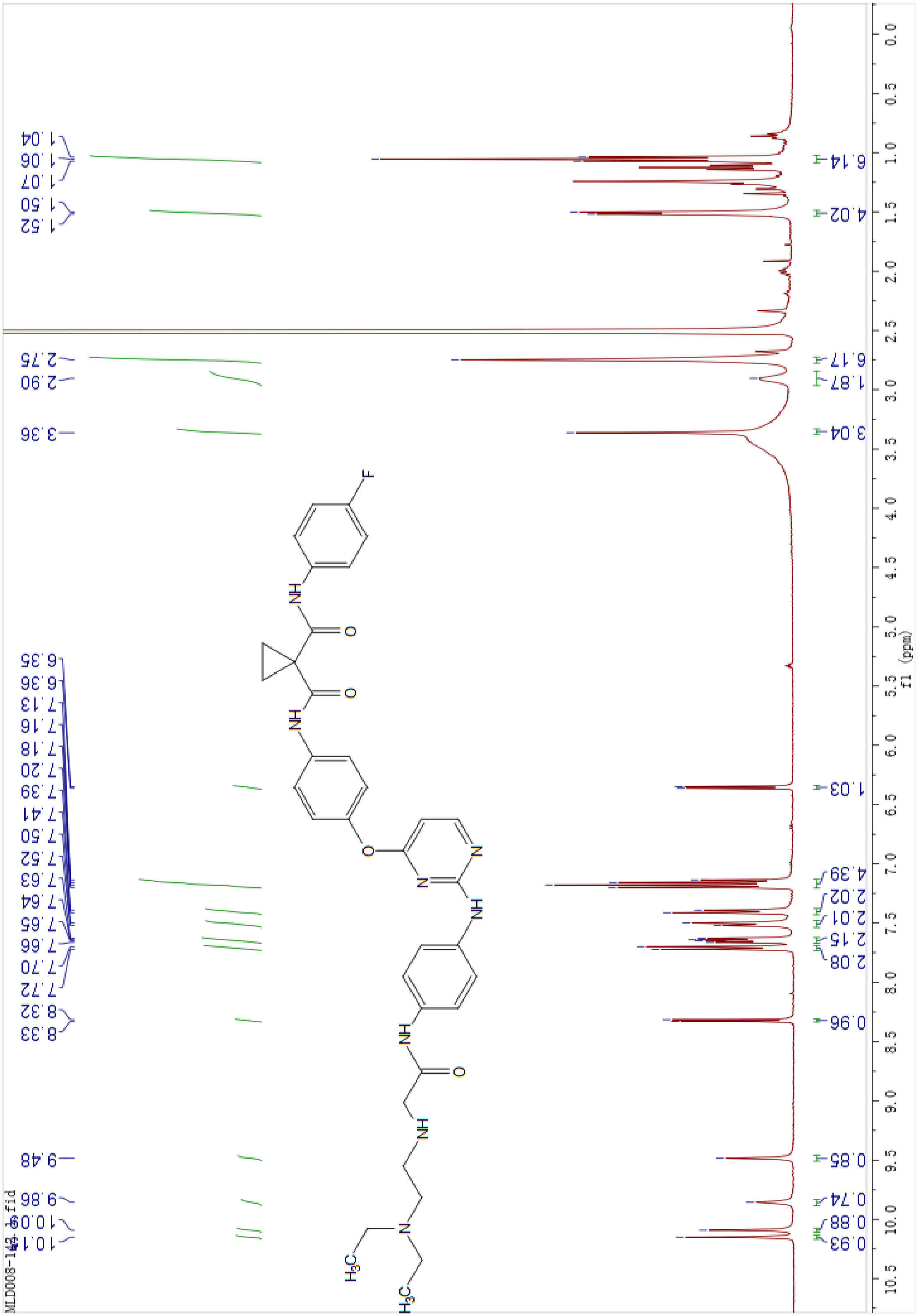

S38: ^{1}H-NMR of **13i**

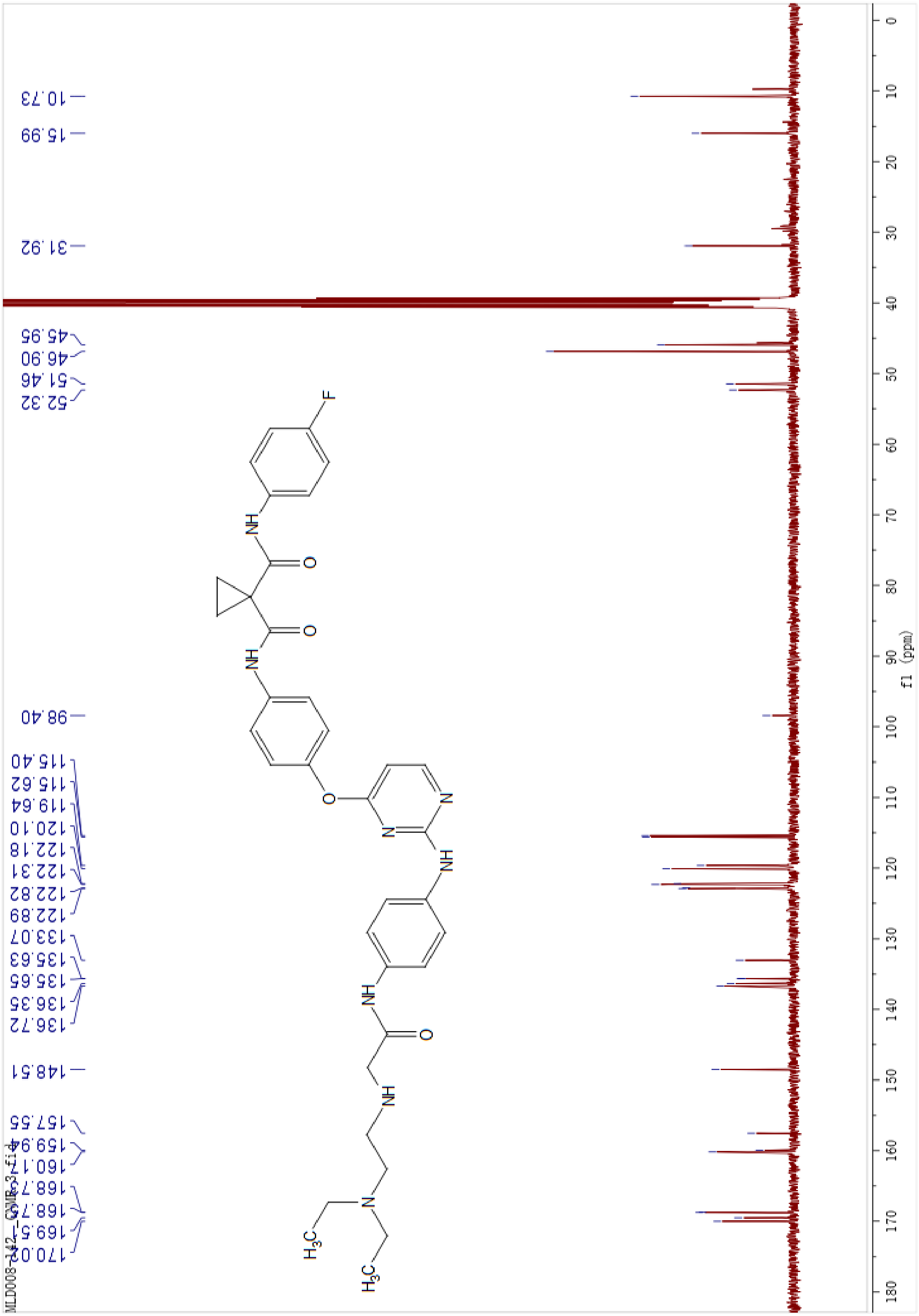

S39: ^{13}C-NMR of **13i**

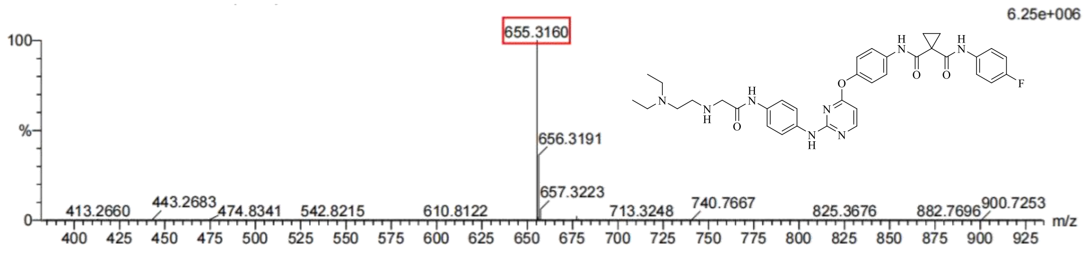

S40: HRMS of **13i**

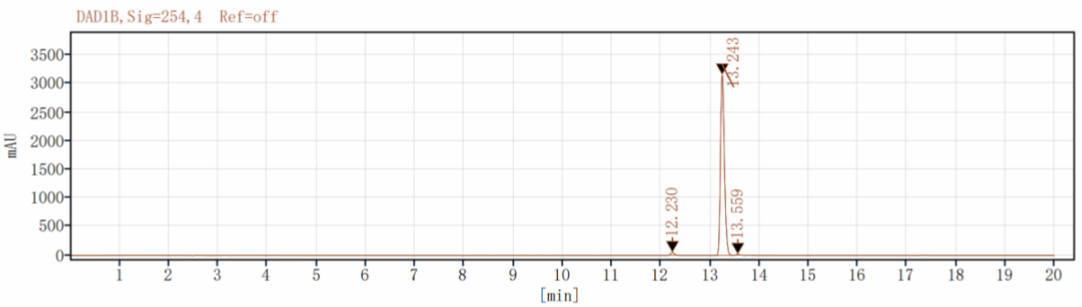

	Retention time (Min)	Peak width (Min)	Area (μV*s)	Height (μV)	% Area
1	12.230	0.18	233.61	49.27	1.31
2	13.243	0.32	17524.41	3158.34	98.35
3	13.559	0.12	61.23	15.36	0.34

S41: HPLC of **13i**

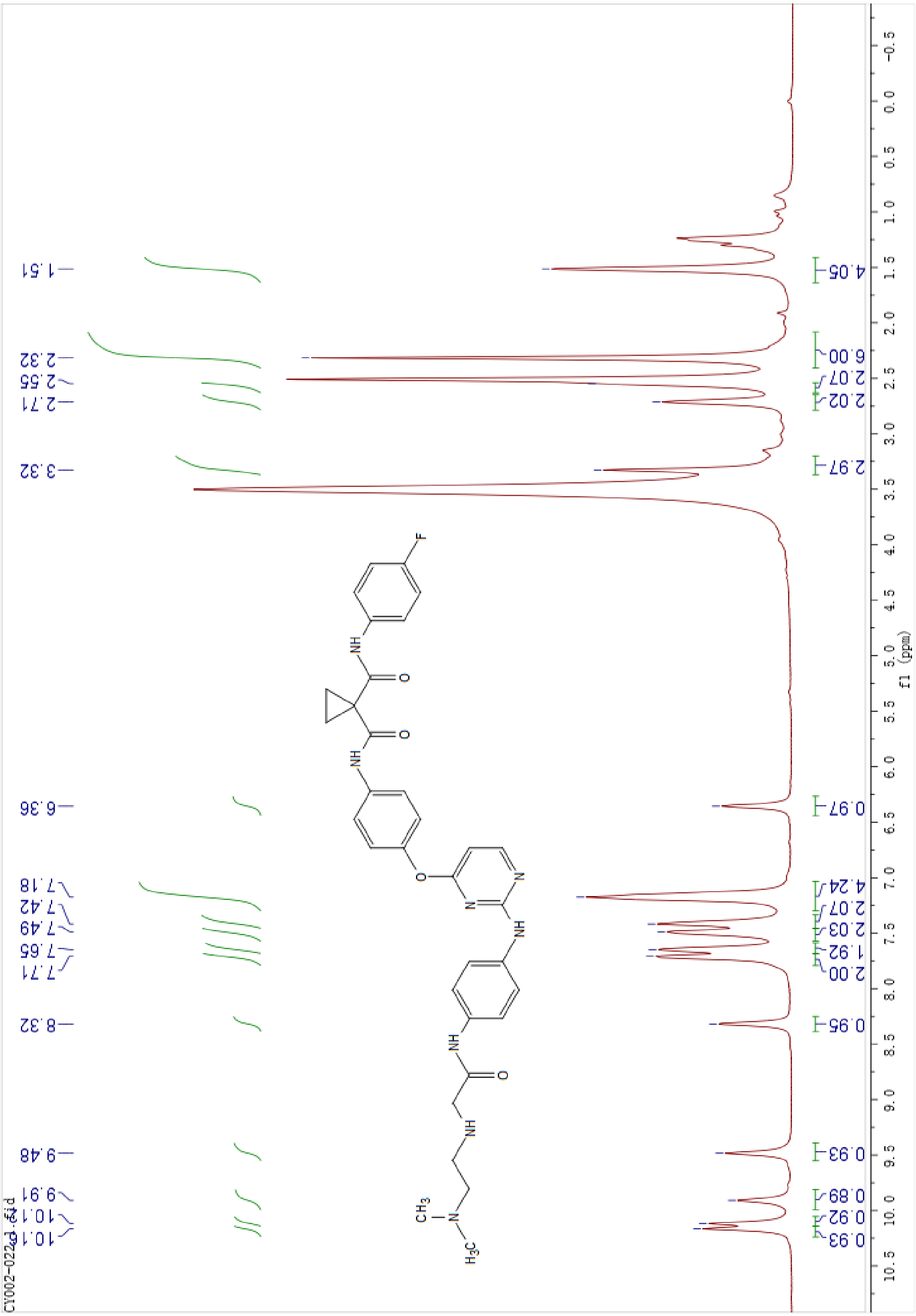

S42: ^{1}H NMR of **13j**

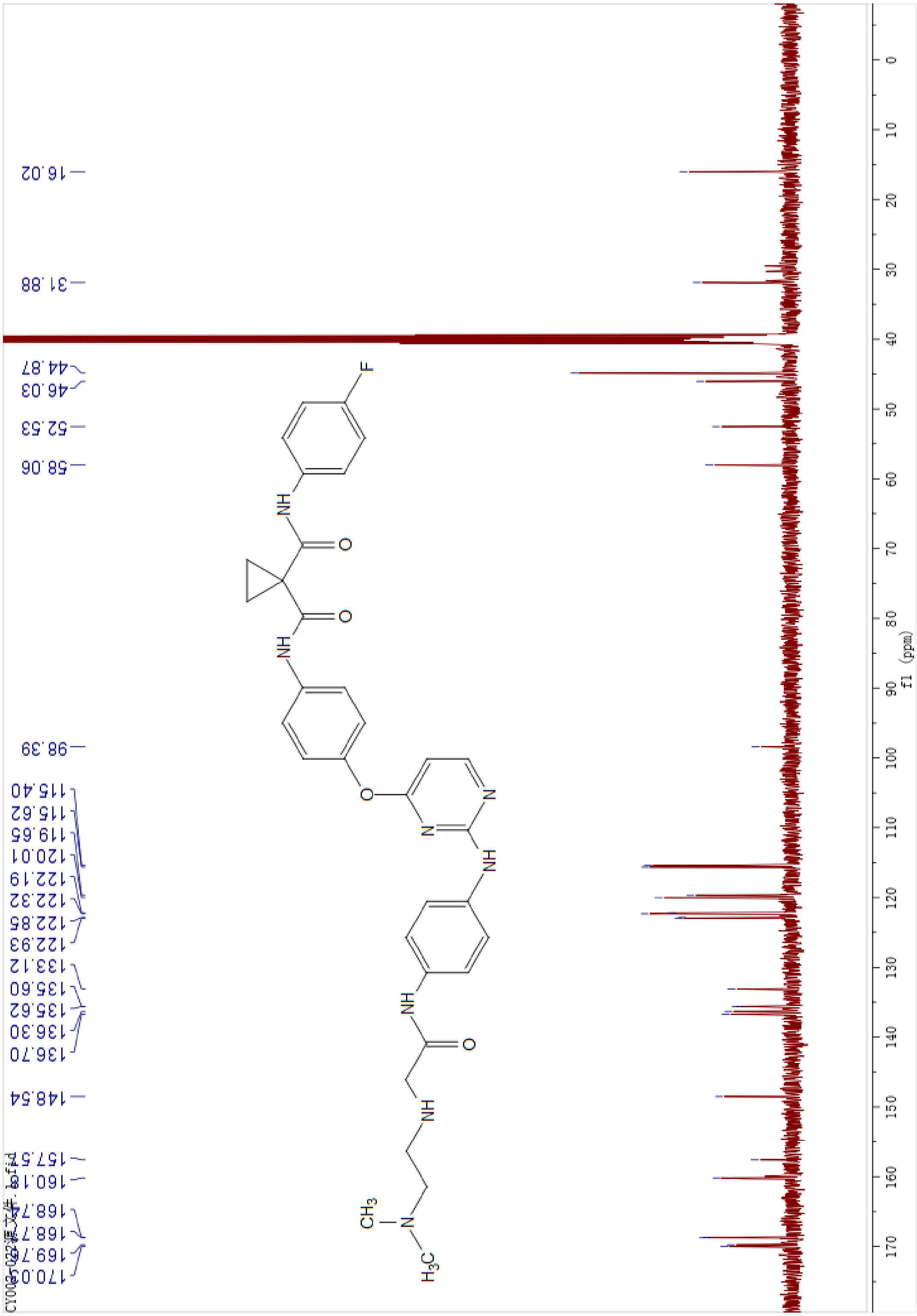

S43: ^{13}C-NMR of **13j**

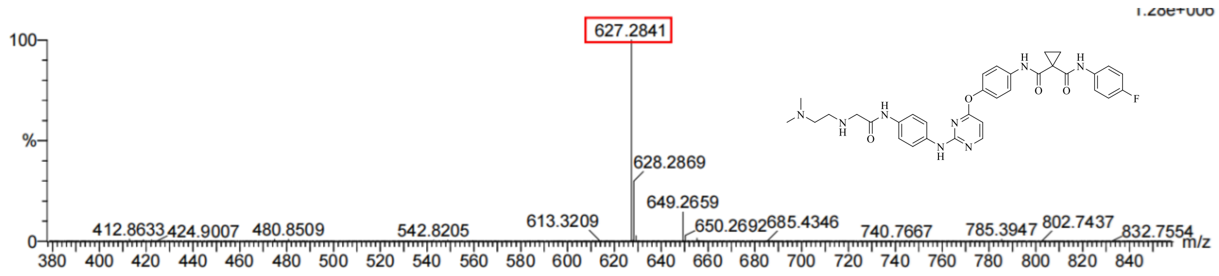

S44: HRMS of **13j**

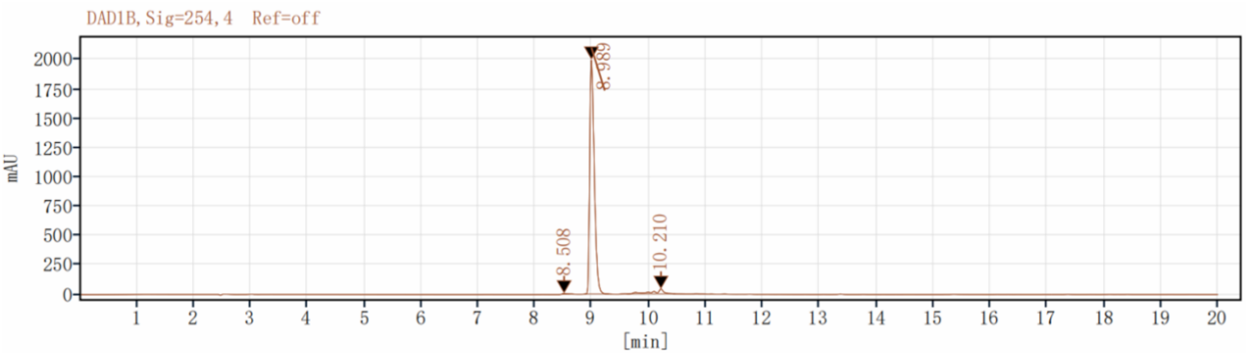

	Retention time (Min)	Peak width (Min)	Area (μV*s)	Height (μV)	% Area
1	8.508	0.16	28.35	6.36	0.24
2	8.989	0.41	11274.23	1999.87	95.10
3	10.210	0.86	552.18	41.67	4.66

S45: HPLC of **13j**

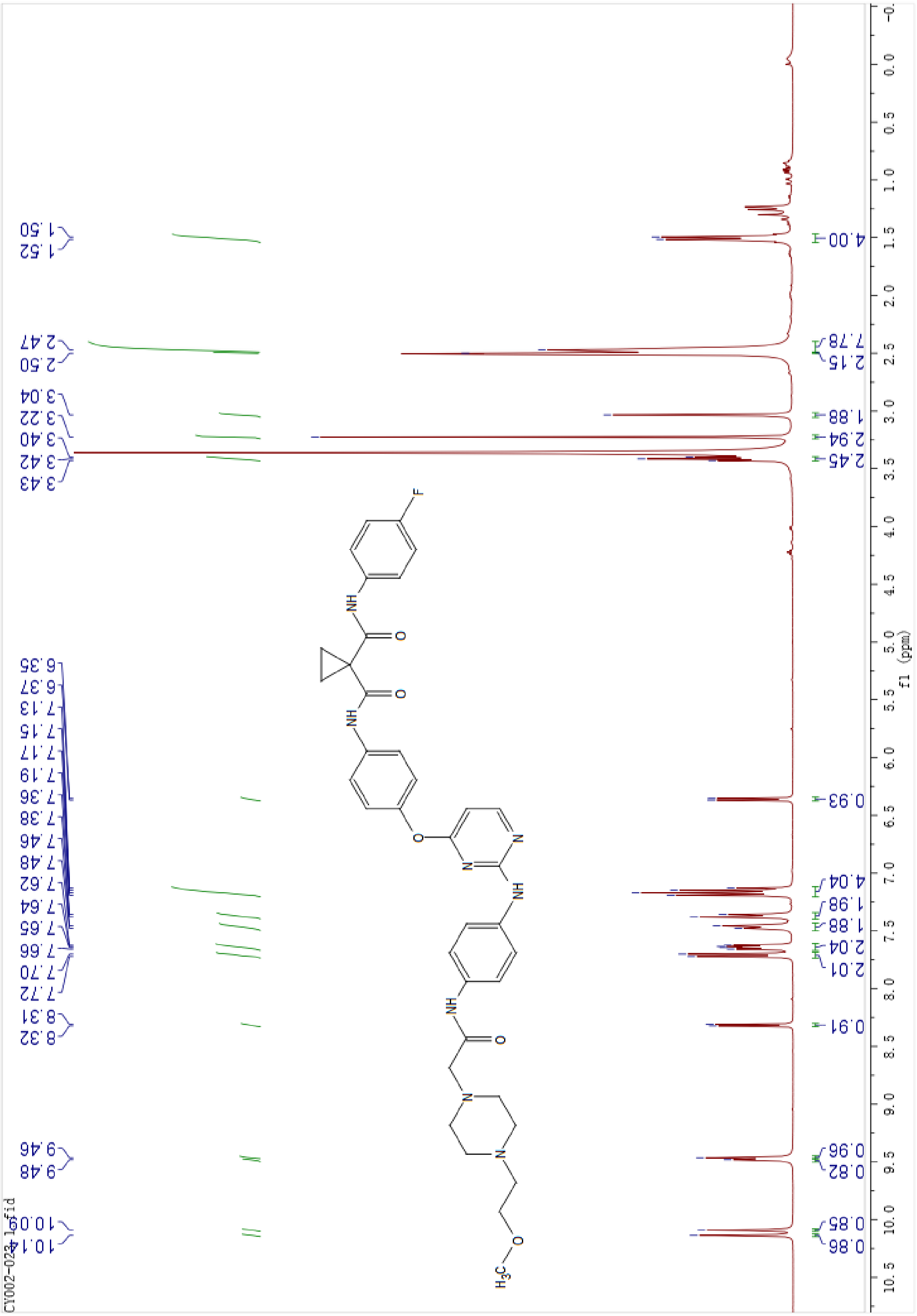

S46: ^{1}H-NMR of **13k**

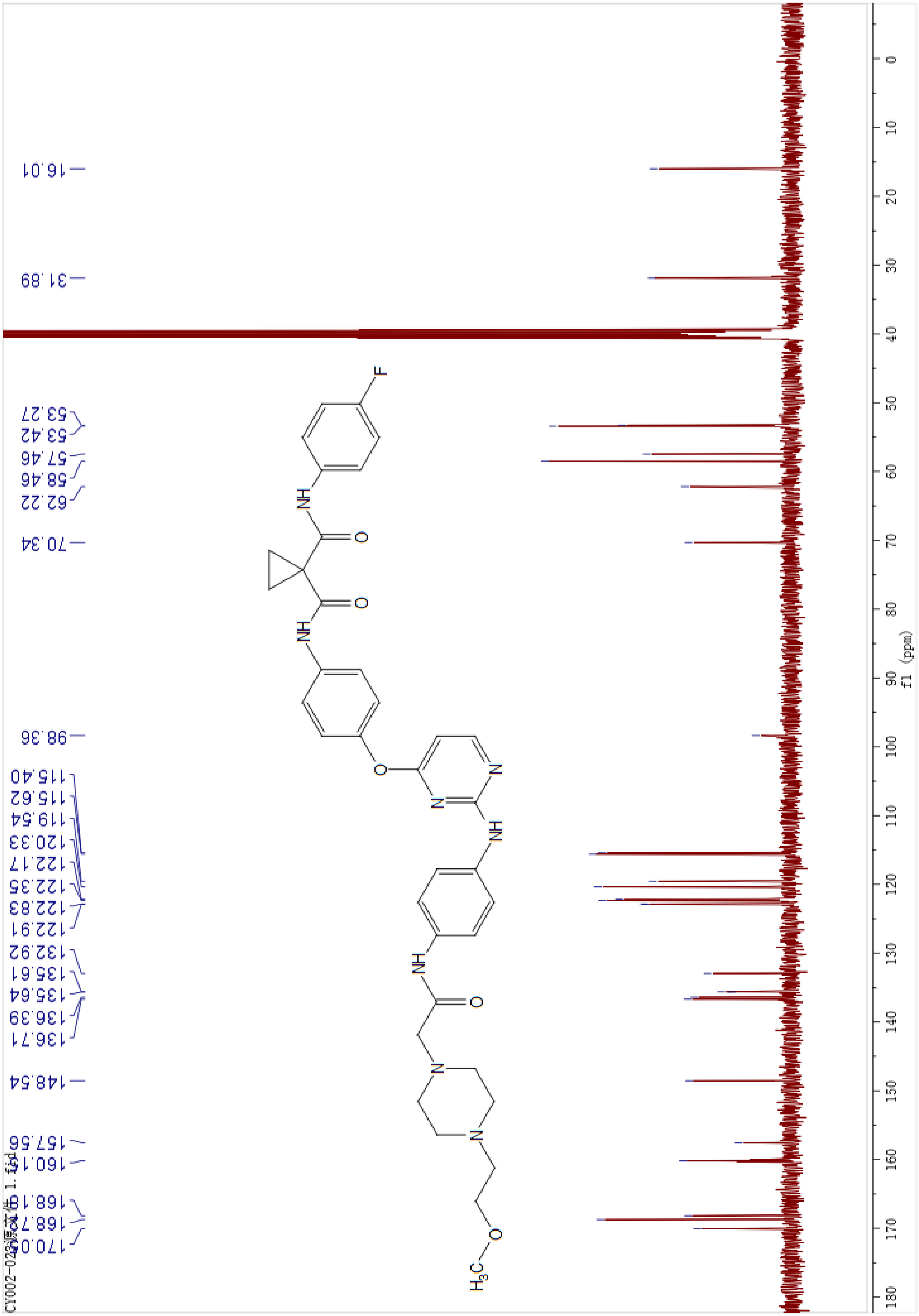

S47: ^{13}C-NMR of **13k**

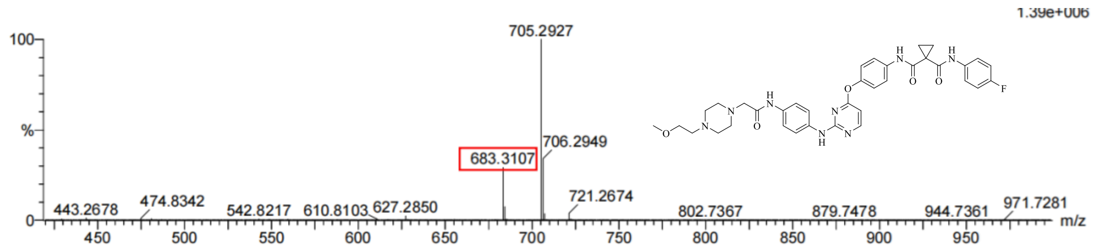

S48: HRMS of **13k**

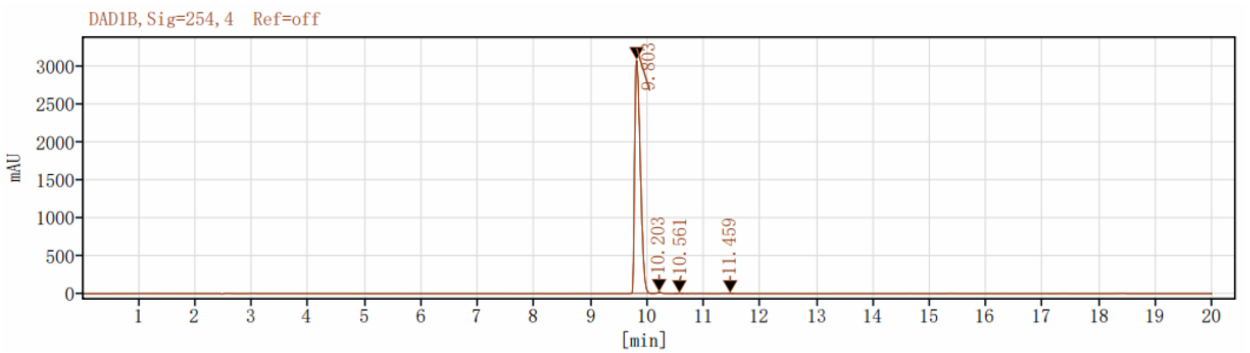

	Retention time (Min)	Peak width (Min)	Area (μV*s)	Height (μV)	% Area
1	9.803	0.36	21453.8	3083.36	99.69
2	10.203	0.19	34.54	13.35	0.16
3	10.561	0.16	19.00	4.19	0.09
4	11.459	0.11	13.14	3.67	0.06

S49: HPLC of **13k**

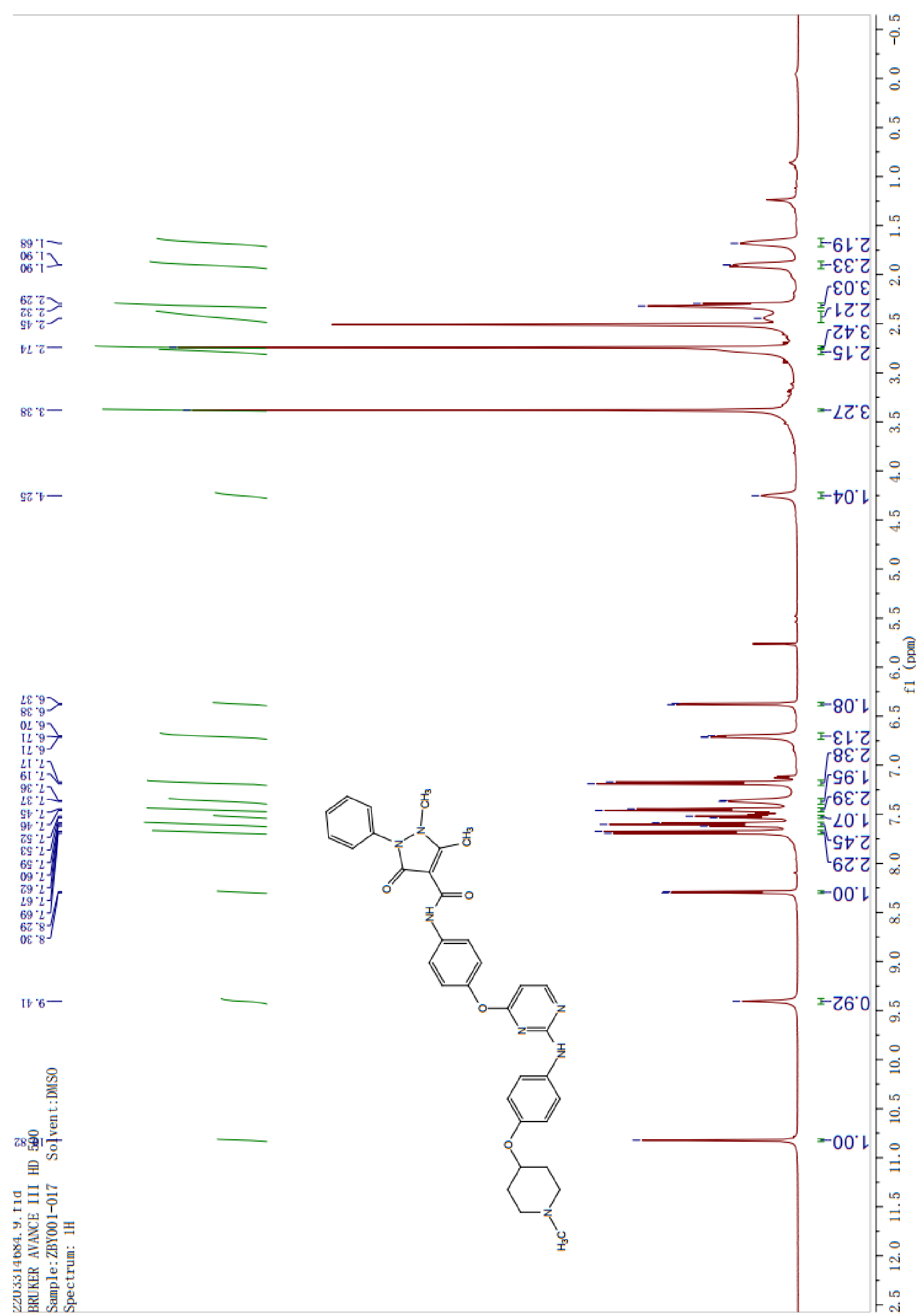

S50: ^{1}H NMR of **17a**

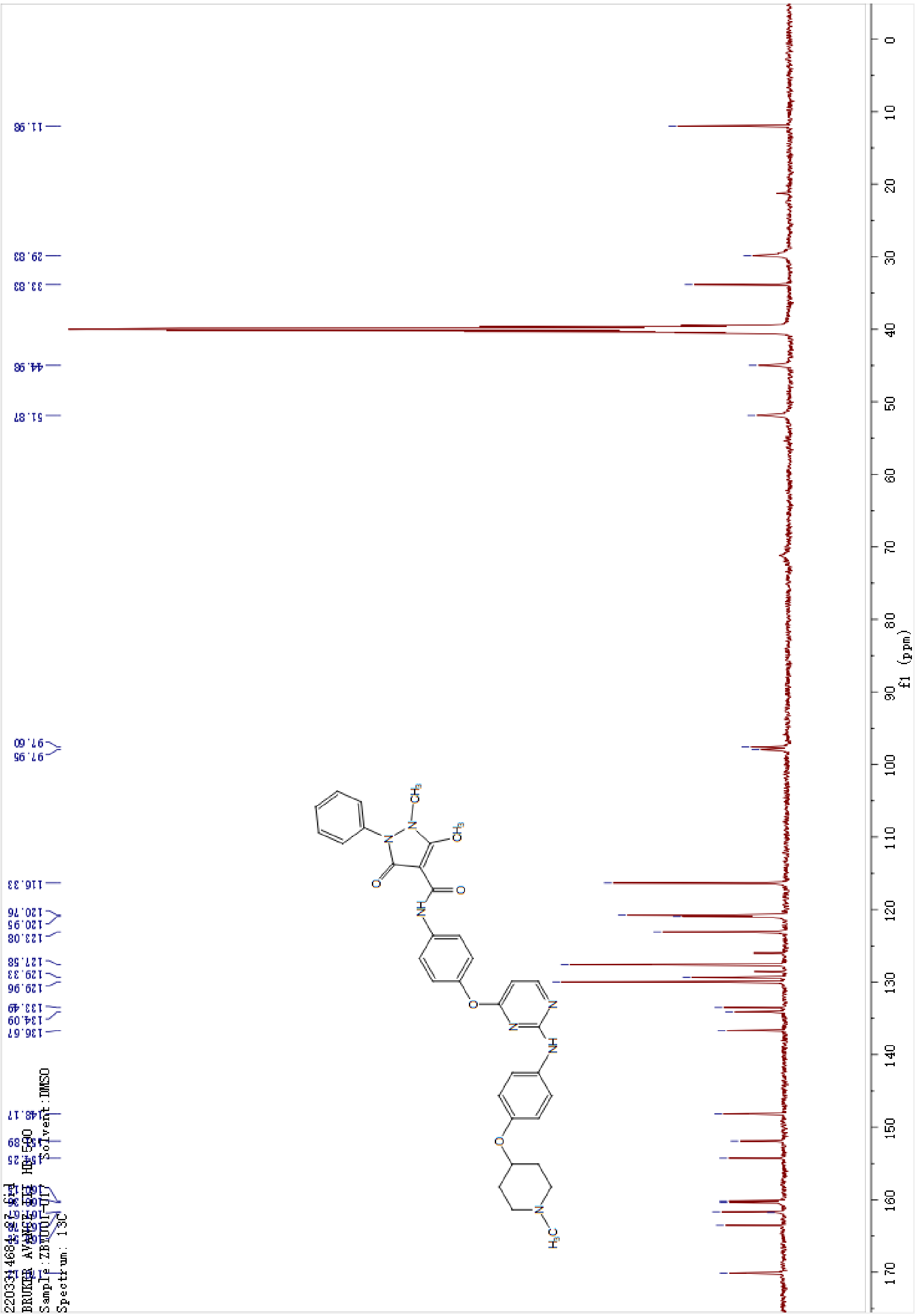

S51: ^{13}C-NMR of **17a**

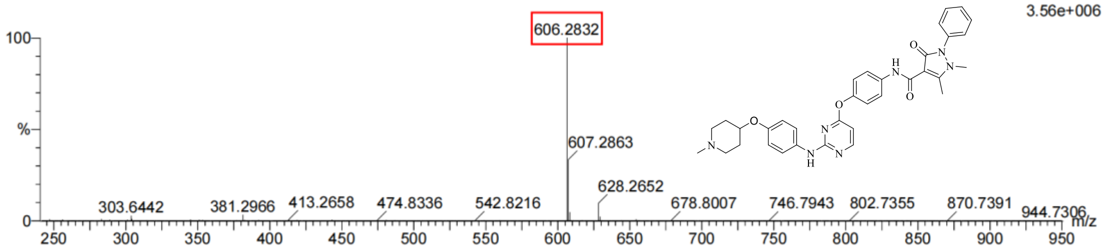

S52: HMRS of **17a**

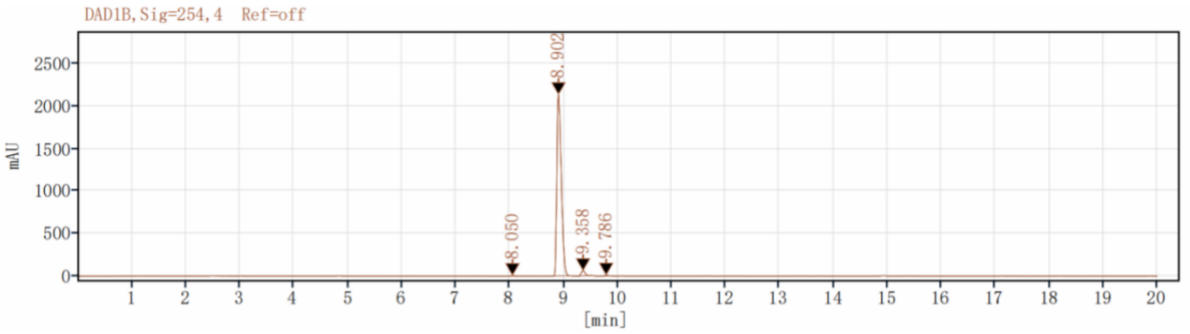

	Retention time(Min)	Peak width(Min)	Area (μV*s)	Height (μV)	% Area
1	8.050	0.11	18.79	5.68	0.15
2	8.902	0.31	12420.70	2140.94	97.24
3	9.358	0.21	297.67	60.75	2.33
4	9.786	0.13	36.07	8.82	0.28

S53: HPLC of **17a**

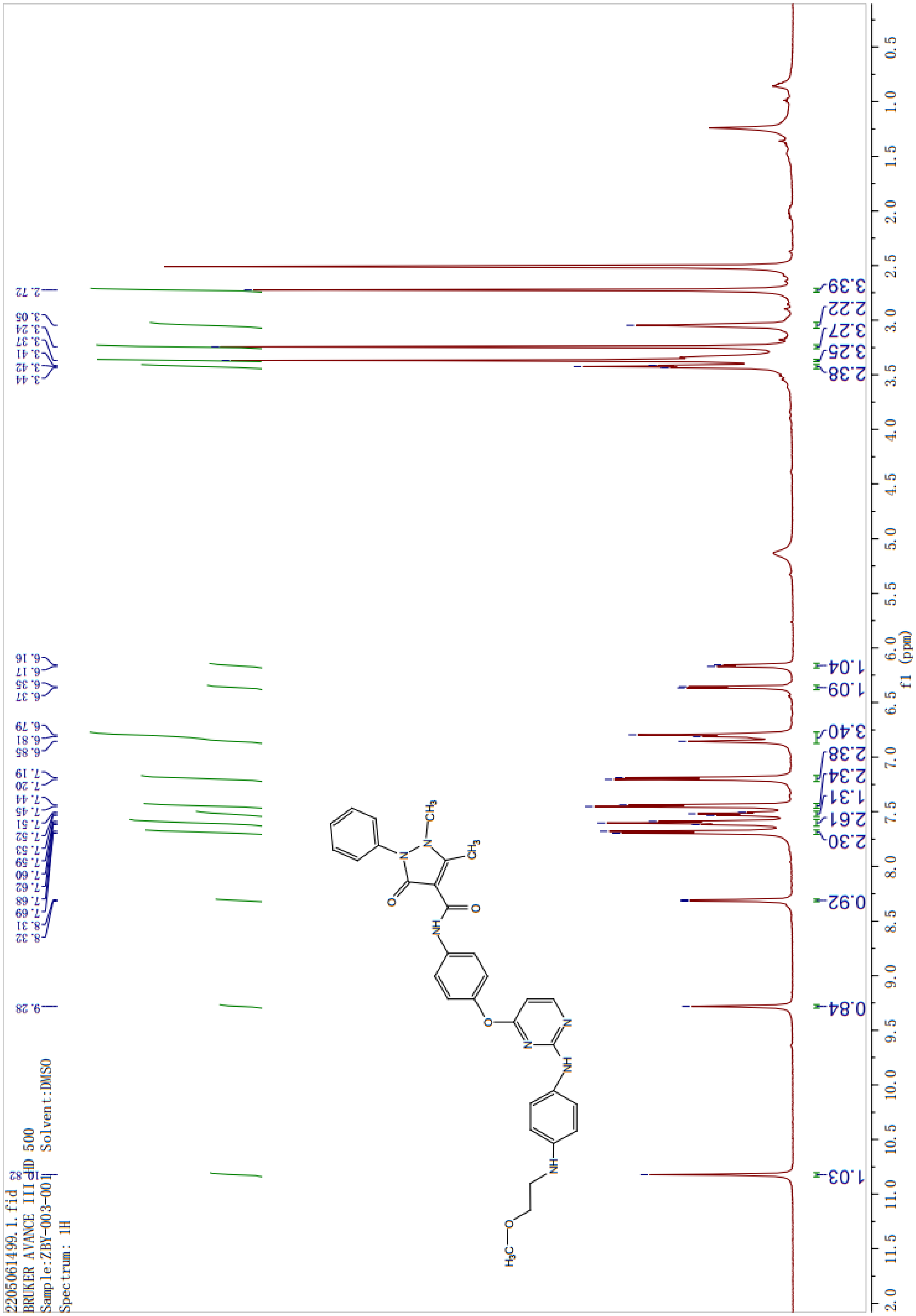

S54: ^{1}H NMR of **17b**

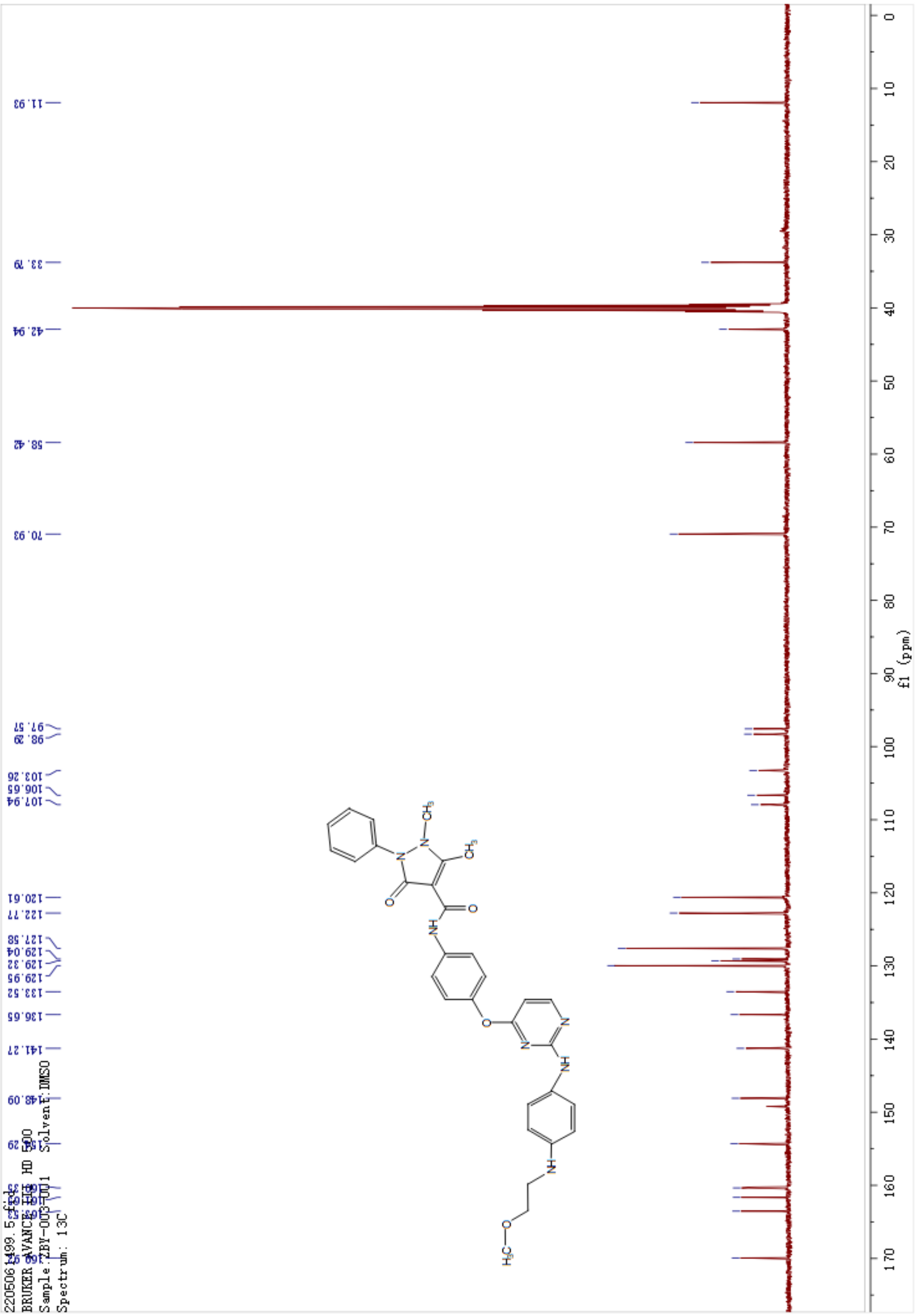

S55: ^{13}C-NMR of **17b**

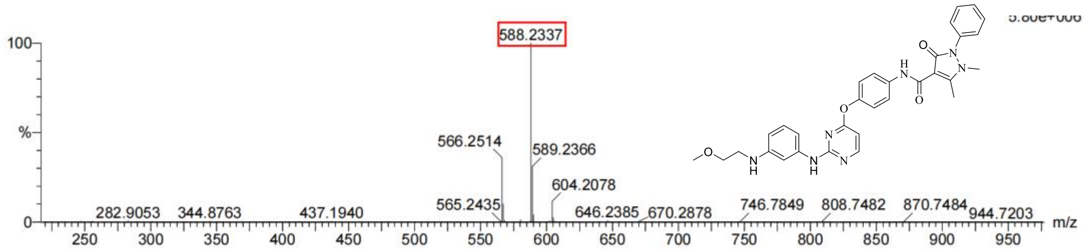

S56: HMRS of **17b**

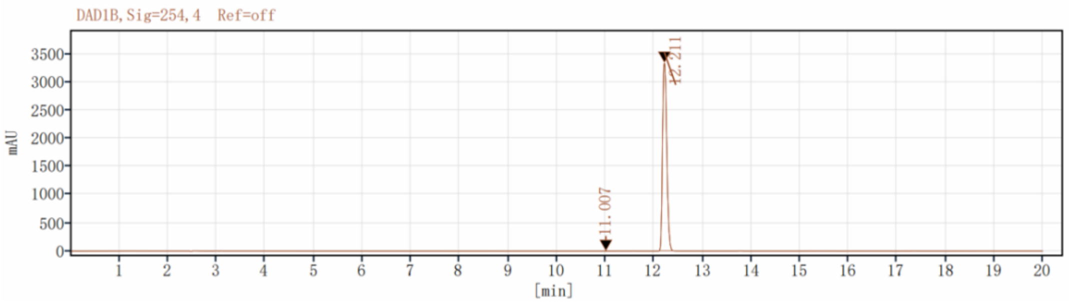

	Retention time (Min)	Peak width (Min)	Area (μV*s)	Height (μV)	% Area
1	11.007	0.06	1.59	0.73	0.01
2	12.211	0.29	20277.27	3343.48	99.99

S57: HPLC of **17b**

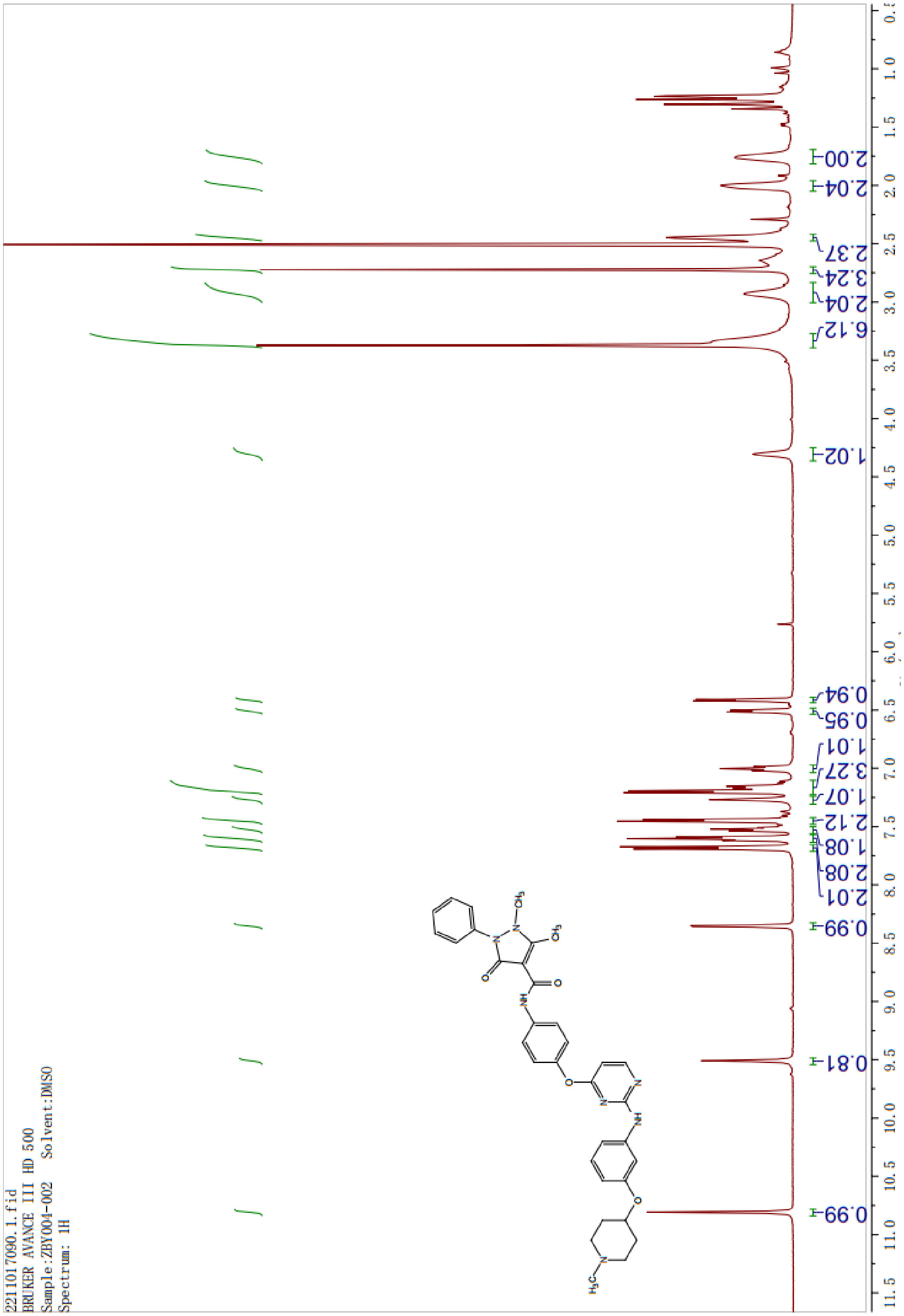

S58: ^{1}H NMR of **17c**

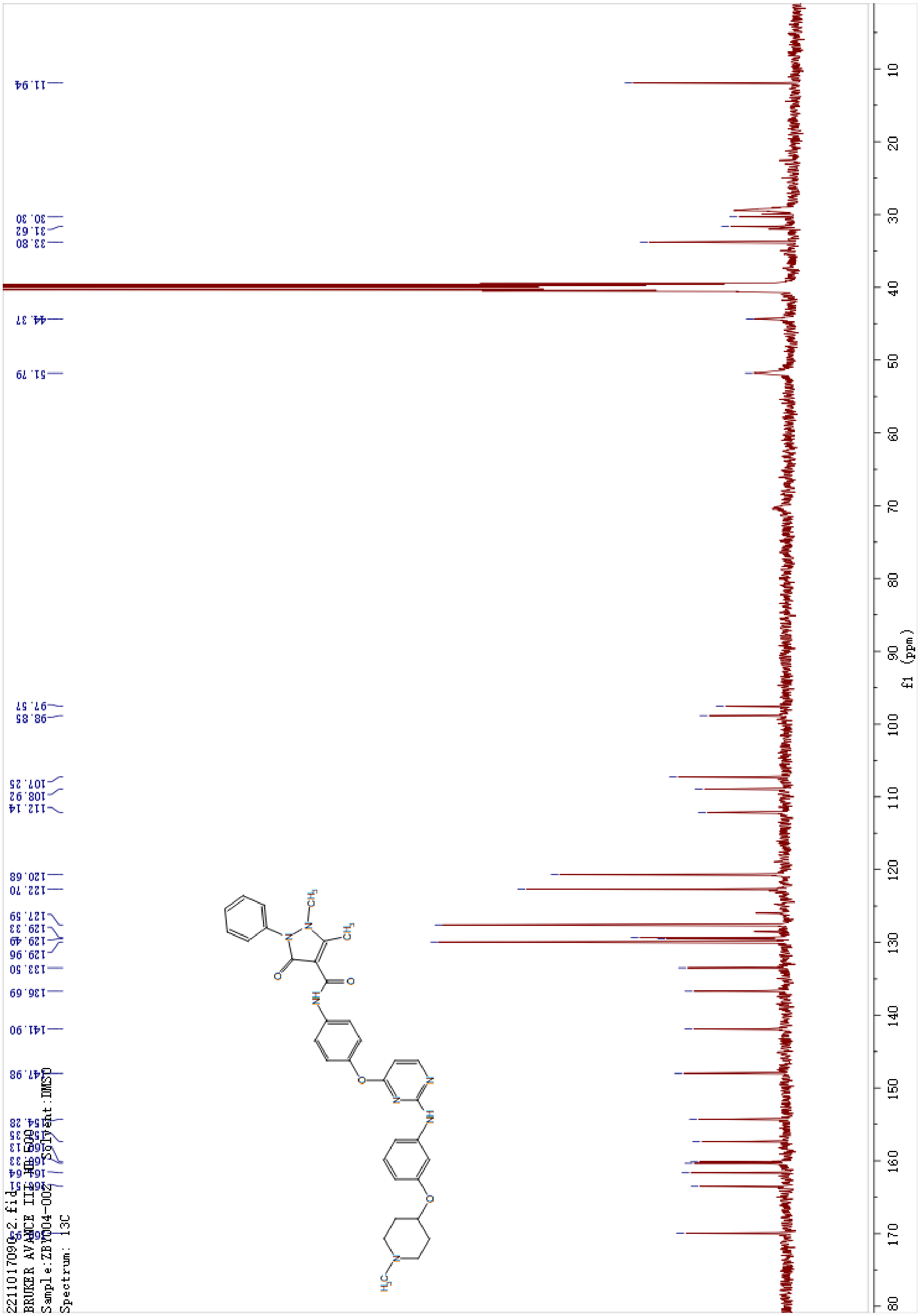

S59: ^{13}C-NMR of **17c**

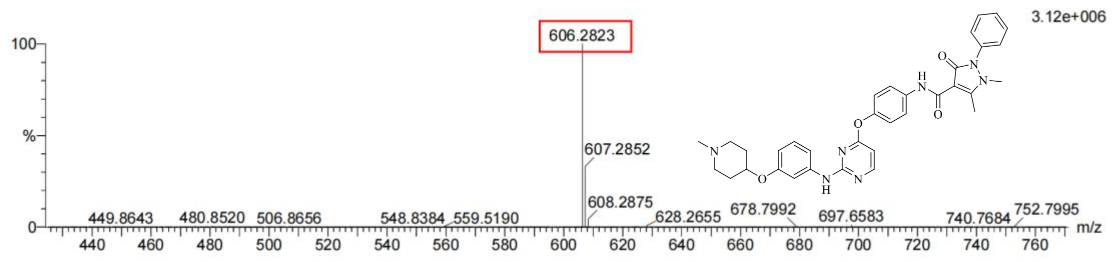

S60: HMRS of **17c**

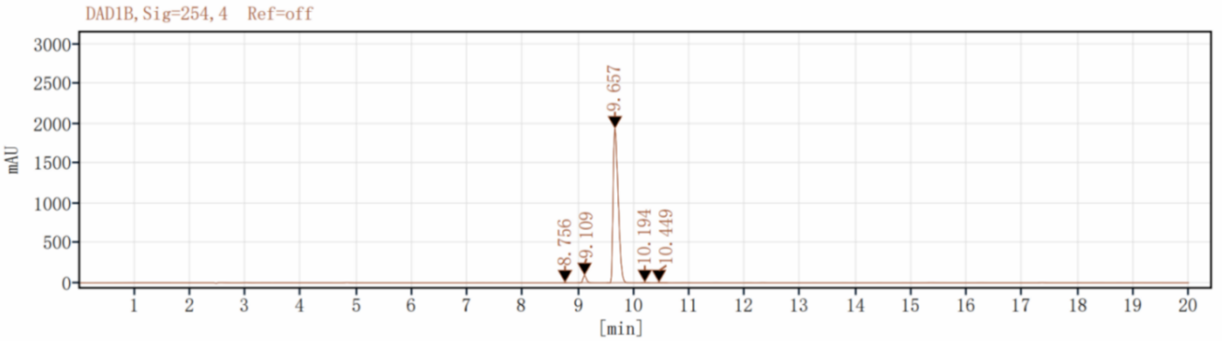

	Retention time(Min)	Peak width(Min)	Area (μV*s)	Height (μV)	% Area
1	8.756	0.07	1.62	0.63	0.01
2	9.109	0.23	382.57	90.27	3.02
3	9.657	0.37	12277.44	1934.03	96.78
4	10.194	0.14	16.24	3.48	0.13
5	10.449	0.19	8.31	1.70	0.07

S61: HPLC of **17c**

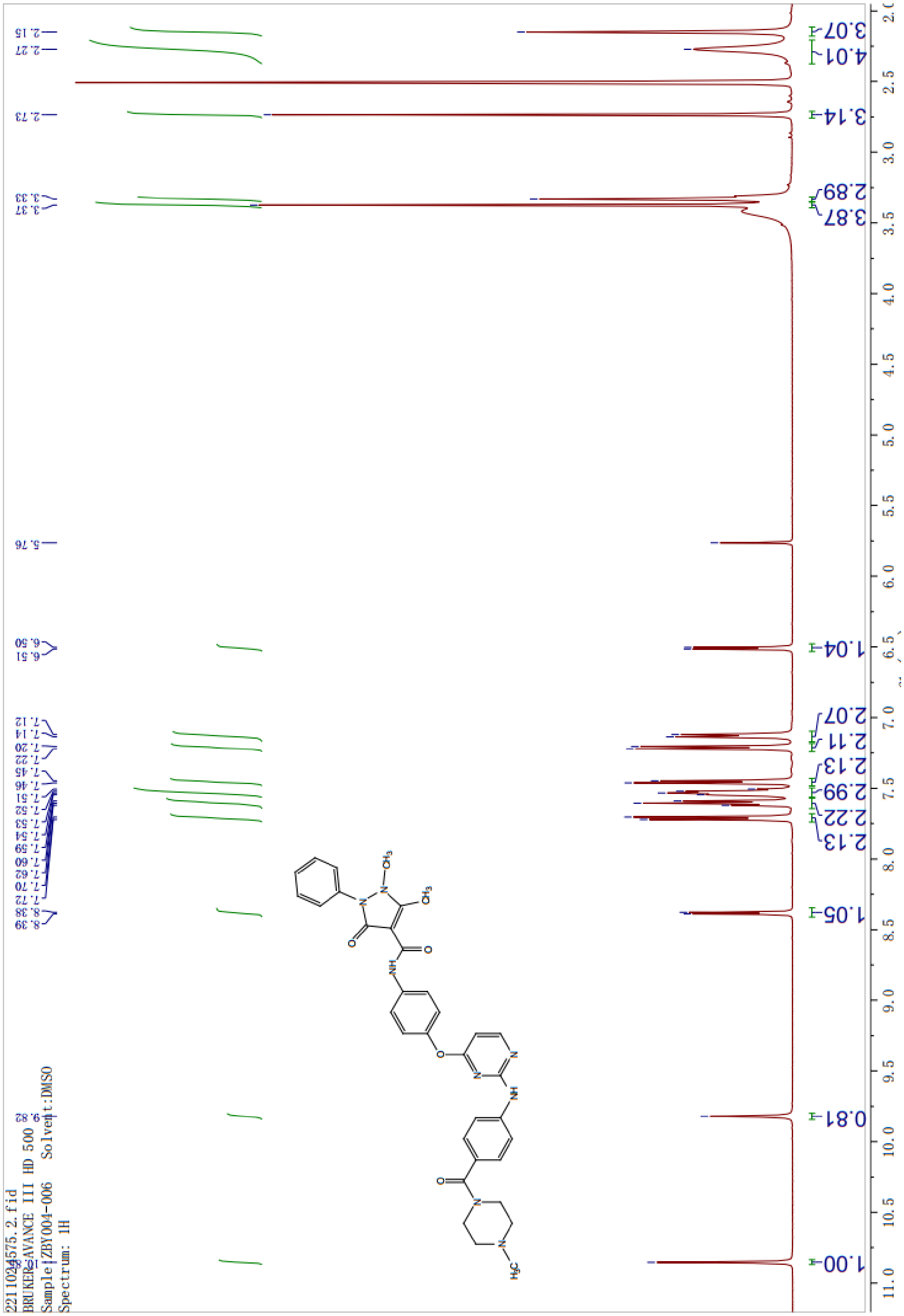

S62: ^{1}H NMR of **17d**

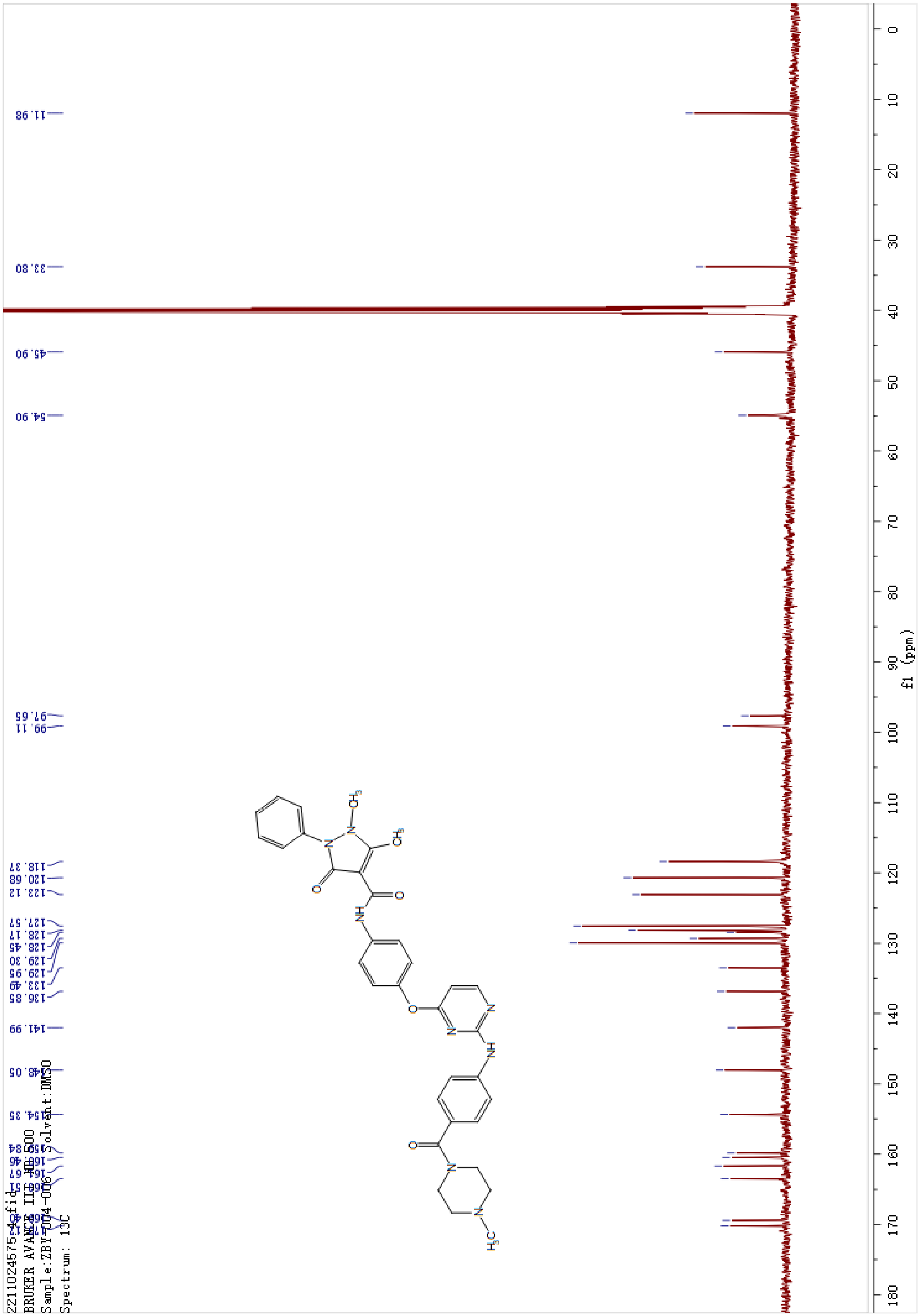

S63: ^{13}C-NMR of **17d**

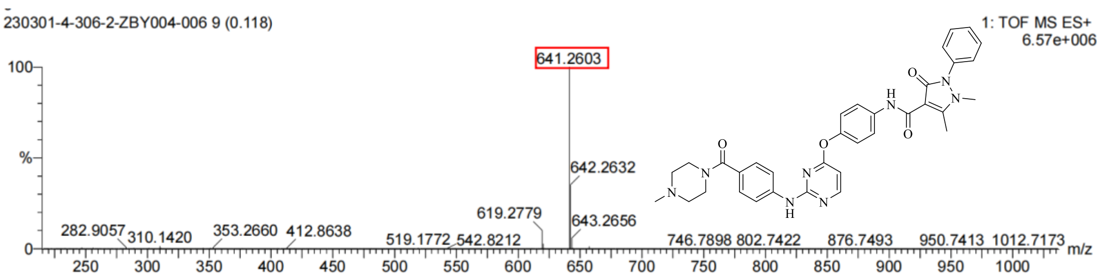

S64: HMRS of **17d**

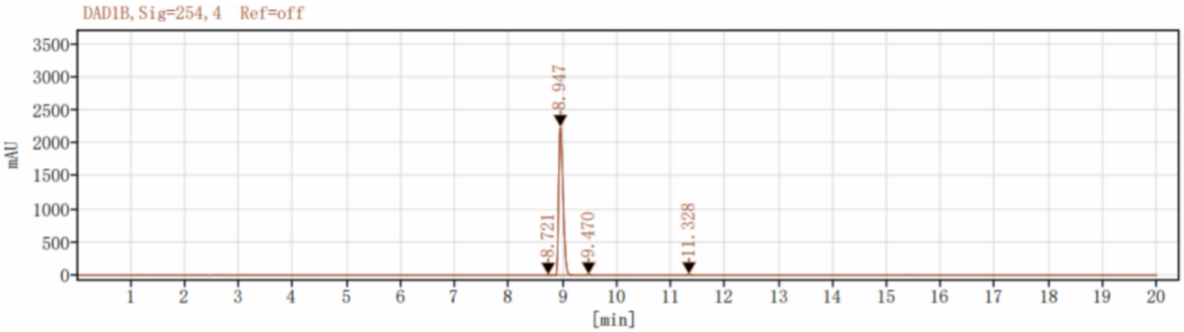

	Retention time (Min)	Peak width (Min)	Area (μV*s)	Height (μV)	% Area
1	8.721	0.17	4.21	2.66	0.03
2	8.947	0.33	12660.70	2251.34	99.37
3	9.470	0.11	29.06	8.21	0.23
4	11.328	0.16	46.62	11.05	0.37

S65: HPLC of **17d**

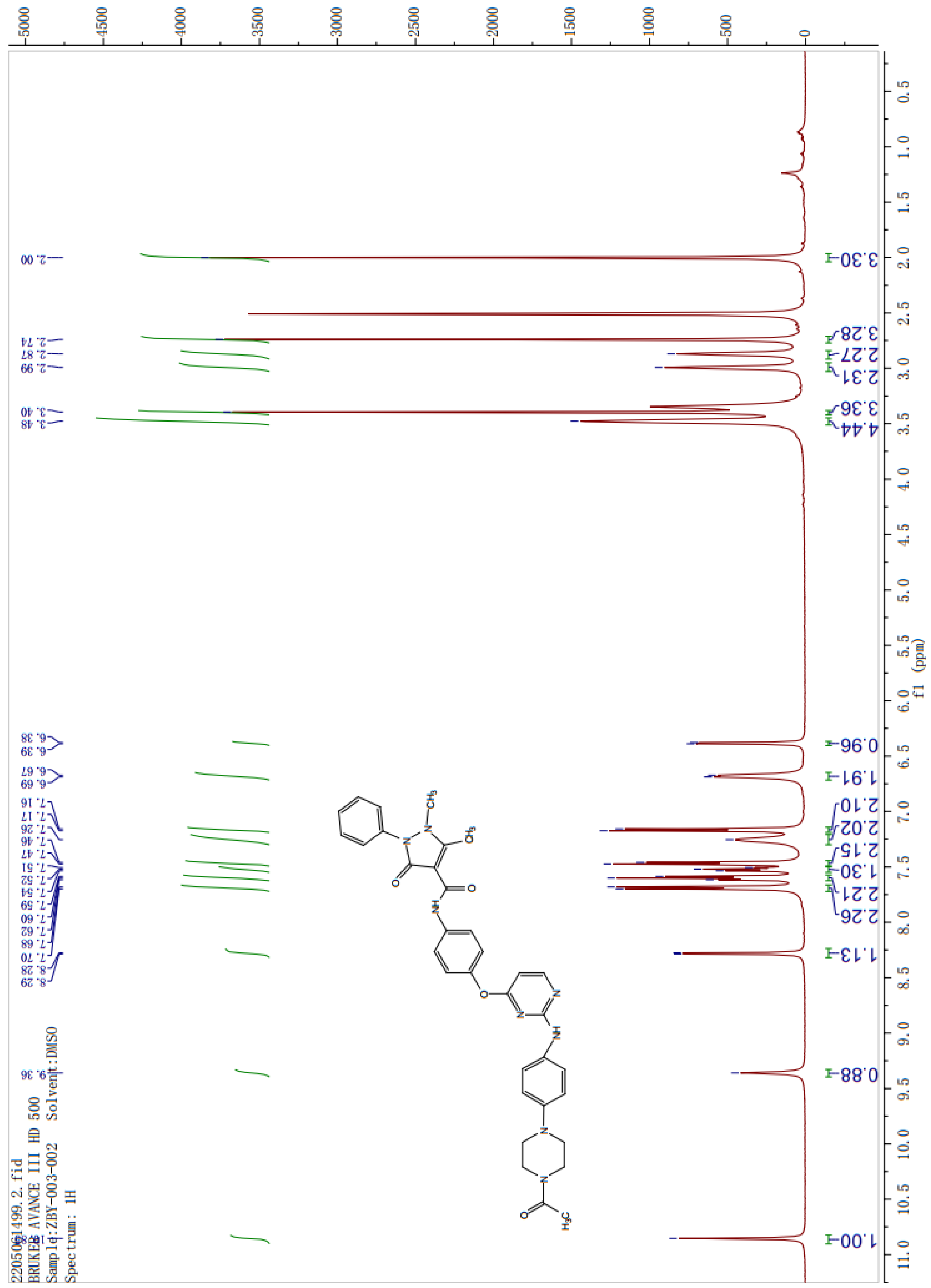

S66: ^{1}H NMR of **17e**

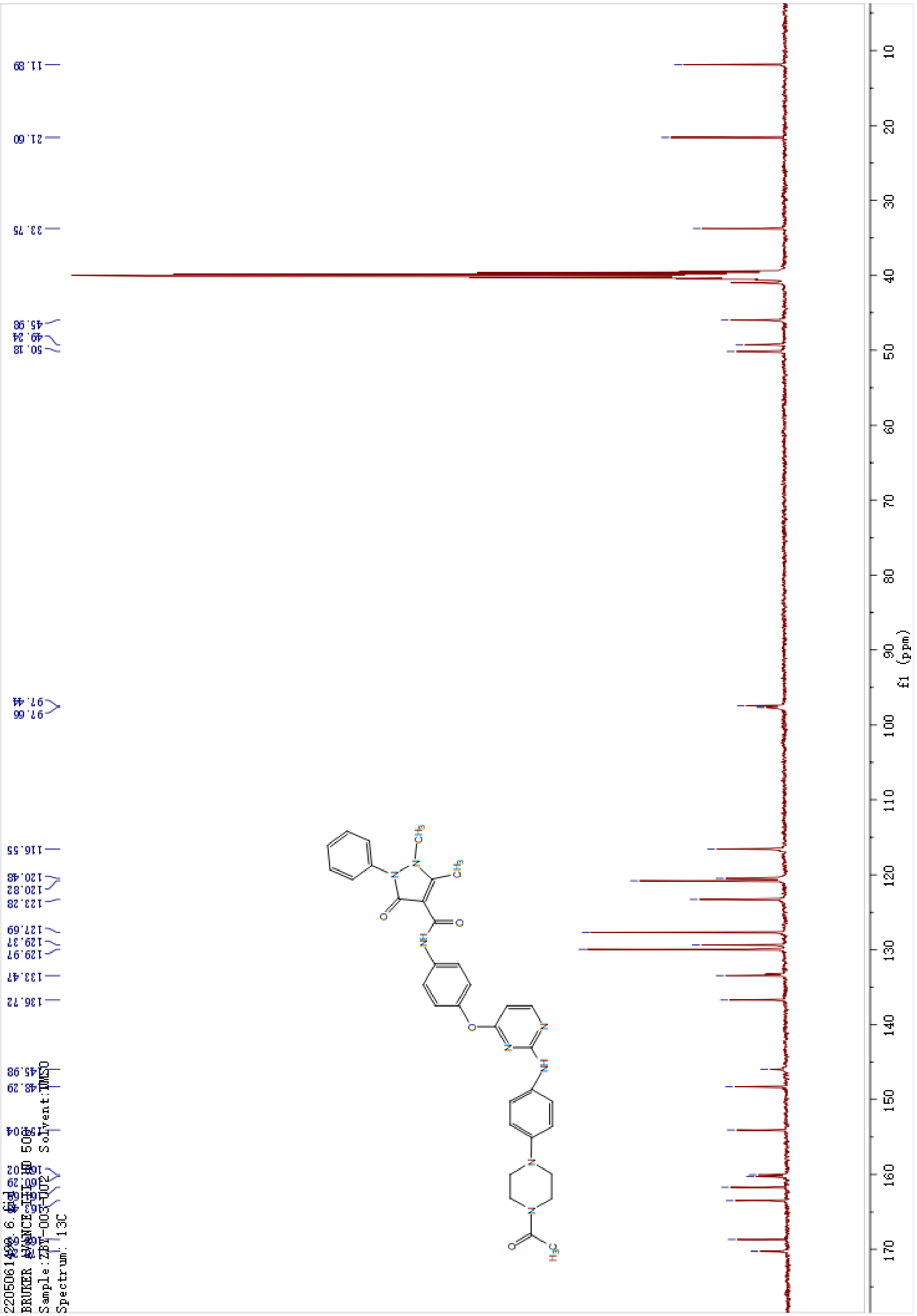

S67: ^{13}C-NMR of **17e**

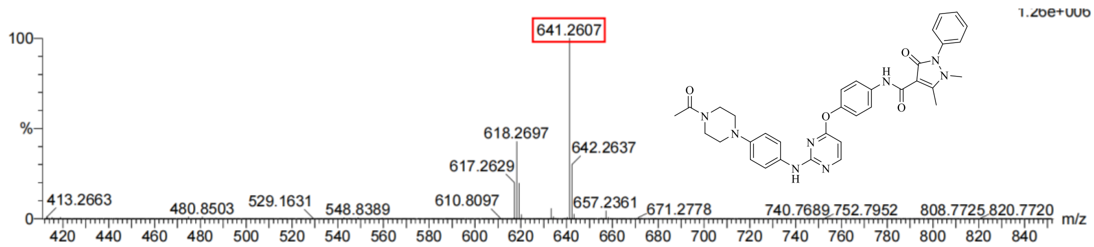

S68: HMRS of **17e**

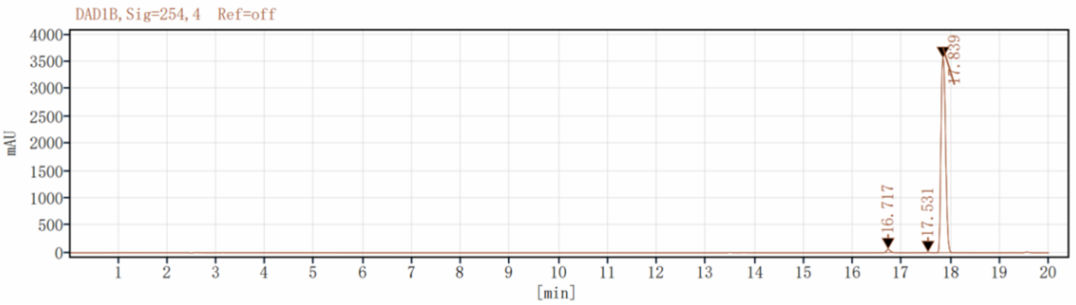

	Retention time (Min)	Peak width (Min)	Area (μV*s)	Height (μV)	% Area
1	16.717	0.20	321.98	60.82	1.37
2	17.531	0.12	28.10	7.31	0.12
3	17.839	0.32	23174.77	3564.80	98.51

S69: HPLC of **17e**

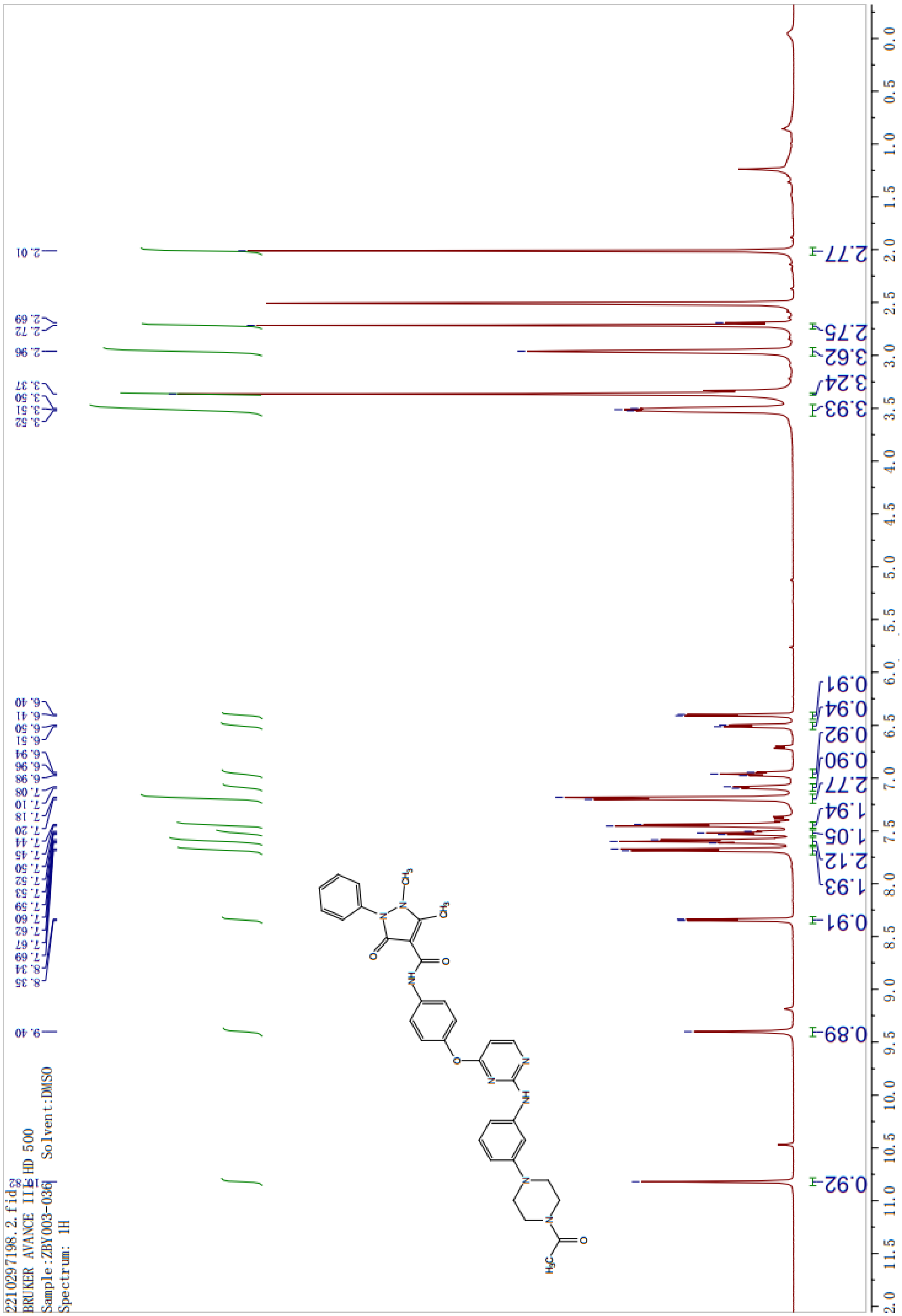

S70: ^{1}H NMR of **17f**

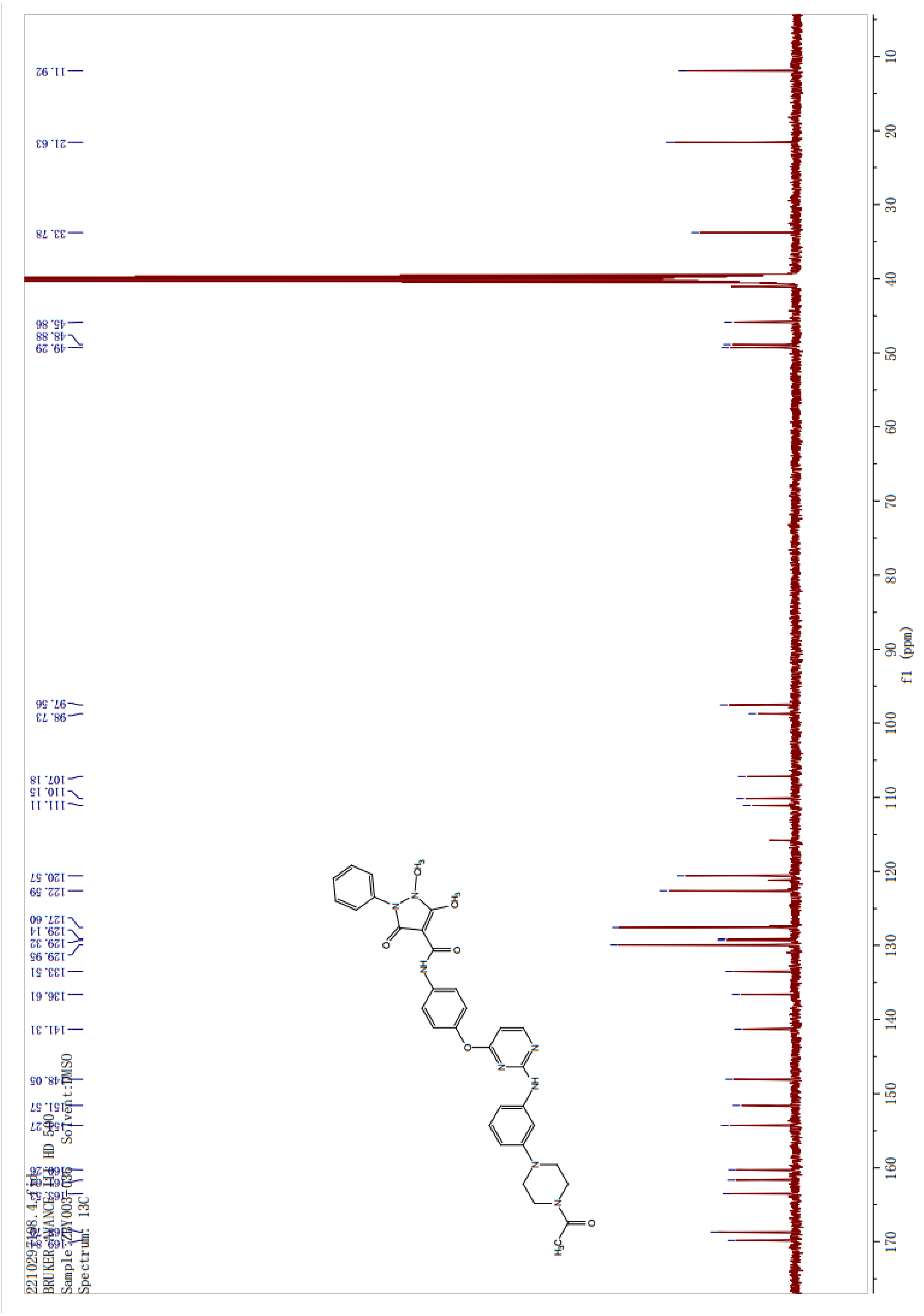

S71: ^{13}C-NMR of **17f**

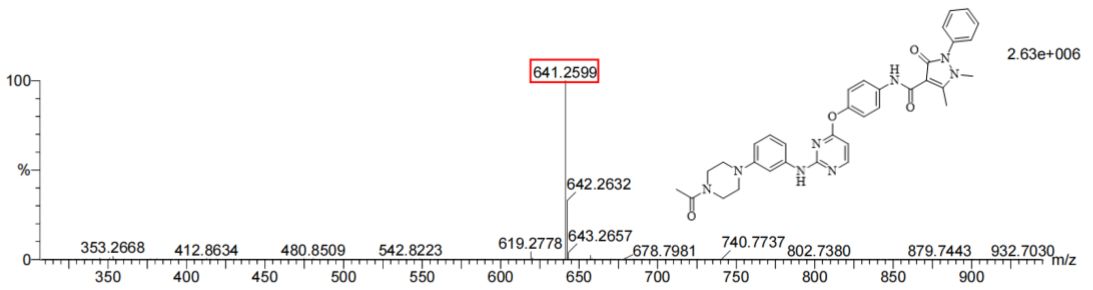

S72: HMRS of **17f**

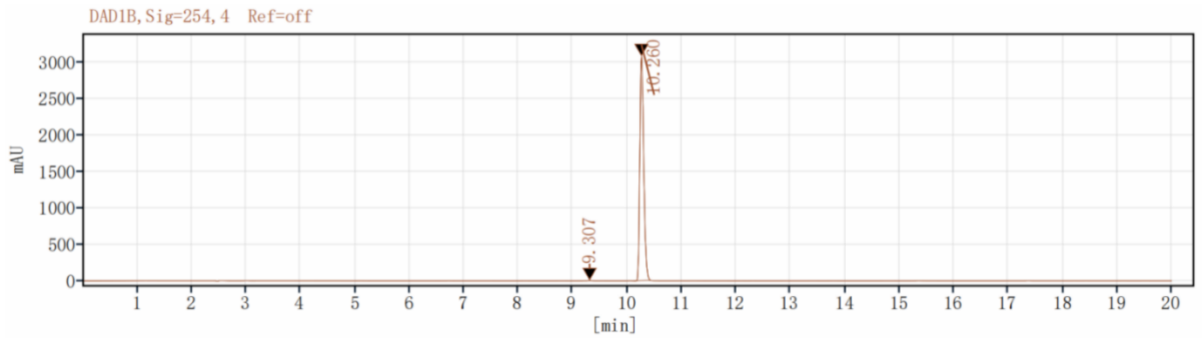

	Retention time (Min)	Peak width (Min)	Area (μV*s)	Height (μV)	% Area
1	9.307	0.09	10.88	3.70	0.07
2	10.260	0.30	15339.84	3078.59	99.93

S73: HPLC of **17f**

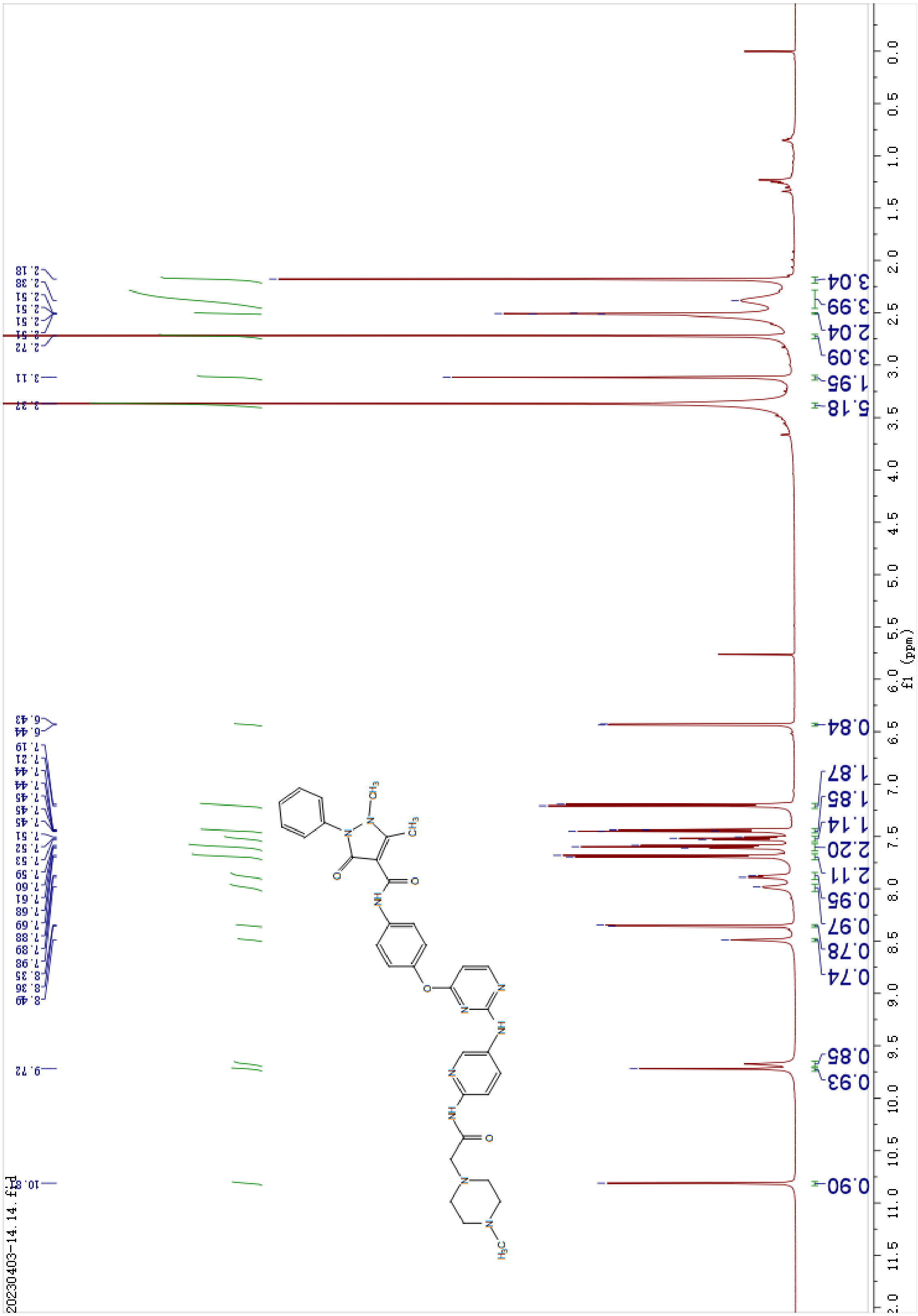

S74: ^{1}H NMR of **17g**

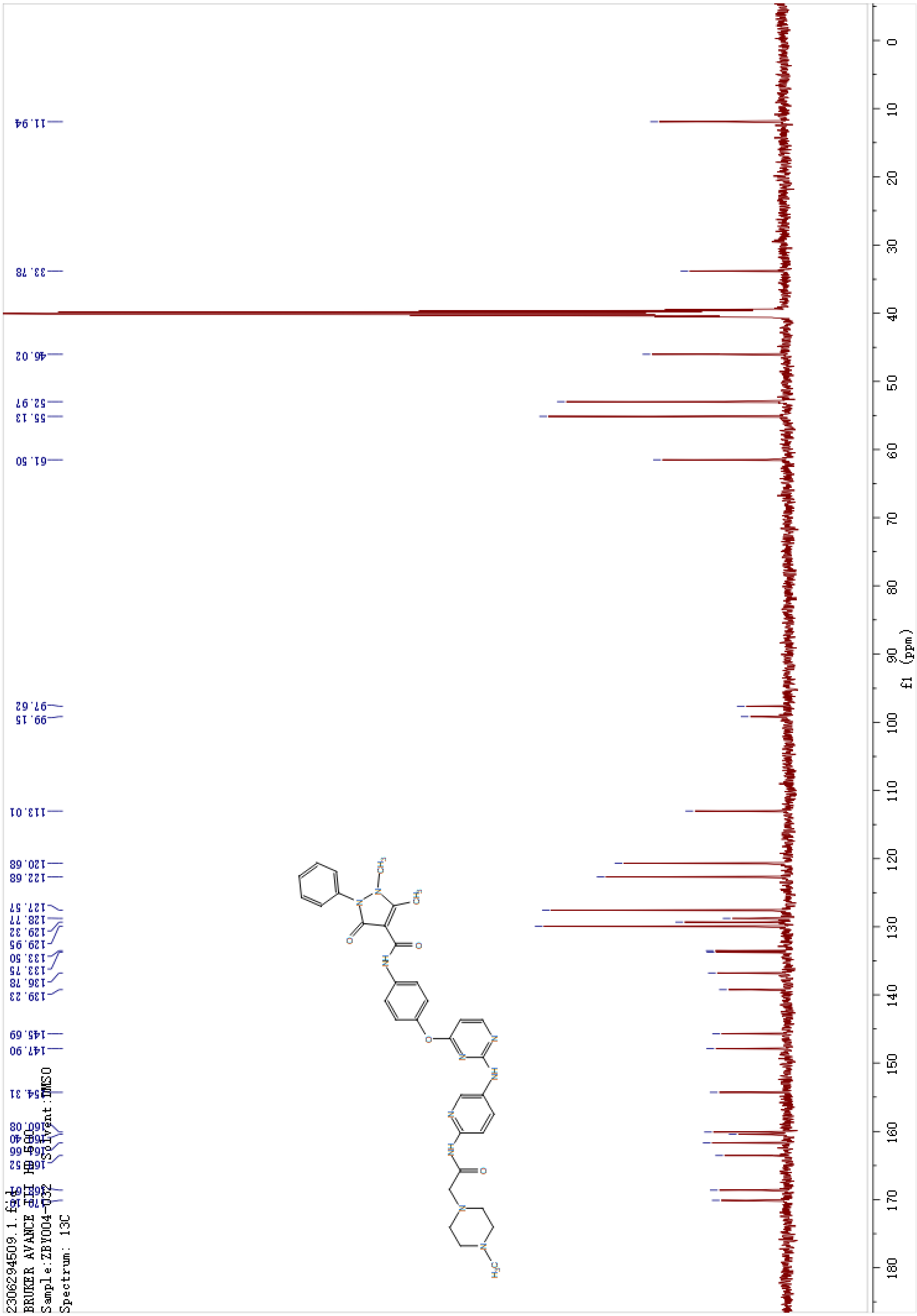

S75: ^{13}C-NMR of **17g**

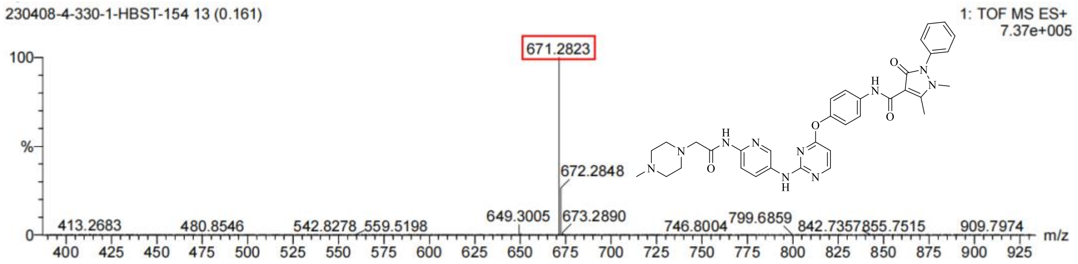

S76: HRMS of **17g**

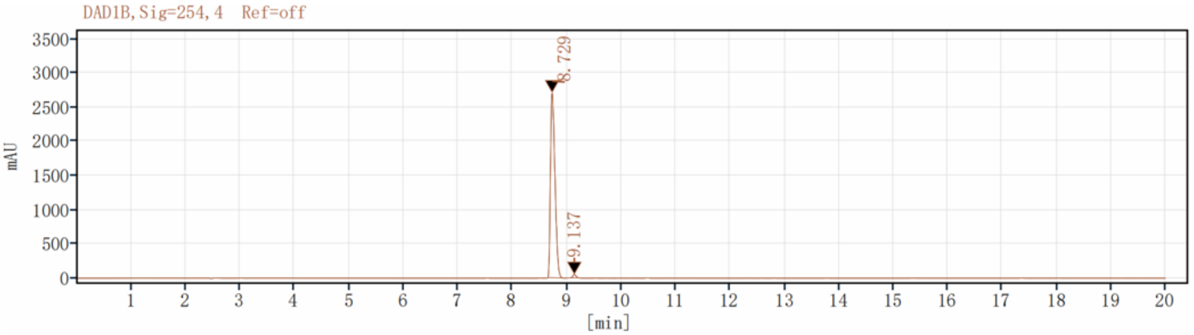

	Retention time (Min)	Peak width (Min)	Area (μV*s)	Height (μV)	% Area
1	8.729	0.30	15590.87	2710.40	98.81
2	9.137	0.26	187.96	48.26	1.19

S77: HPLC of **17g**

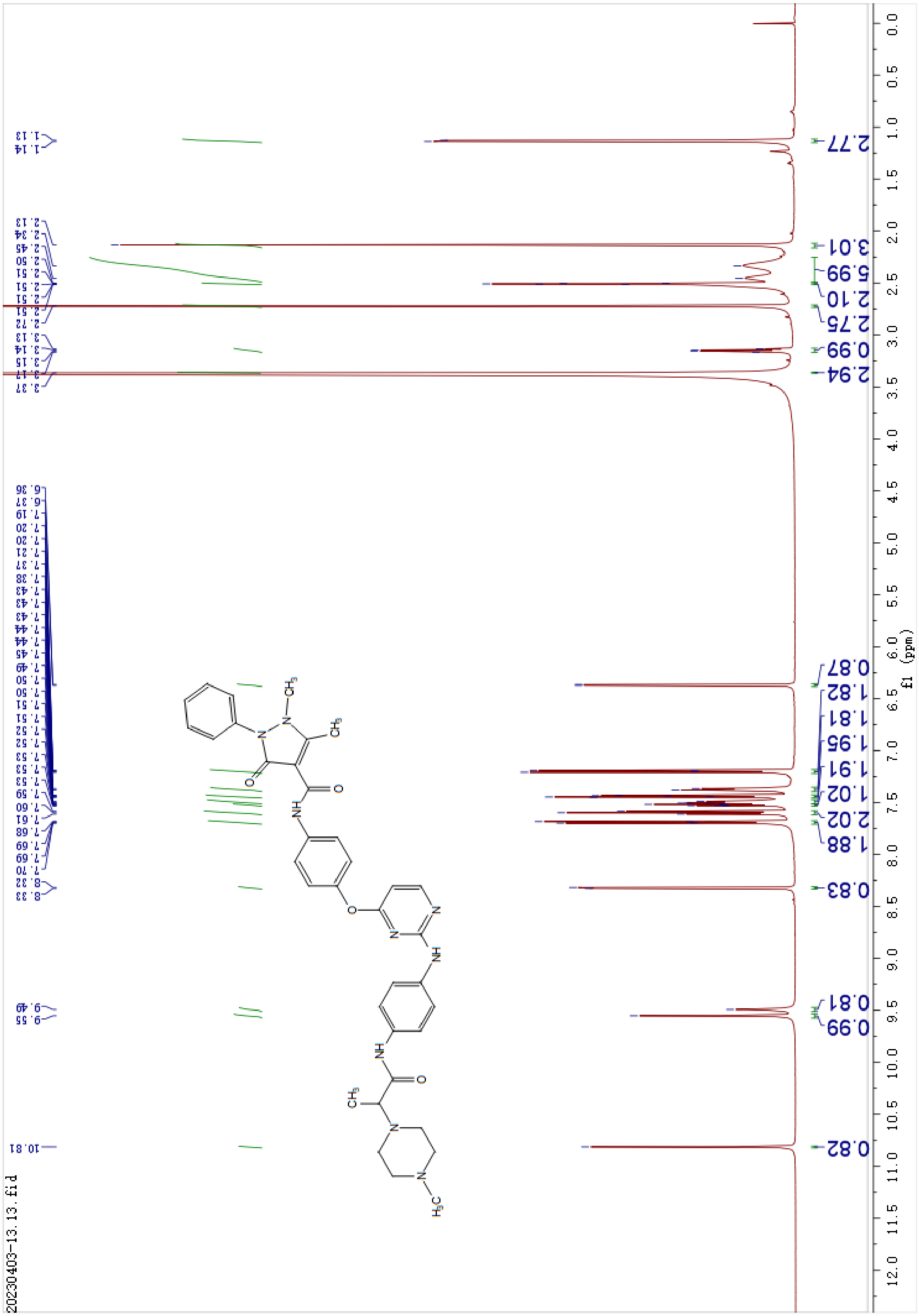

S78: ^{1}H NMR of **17h**

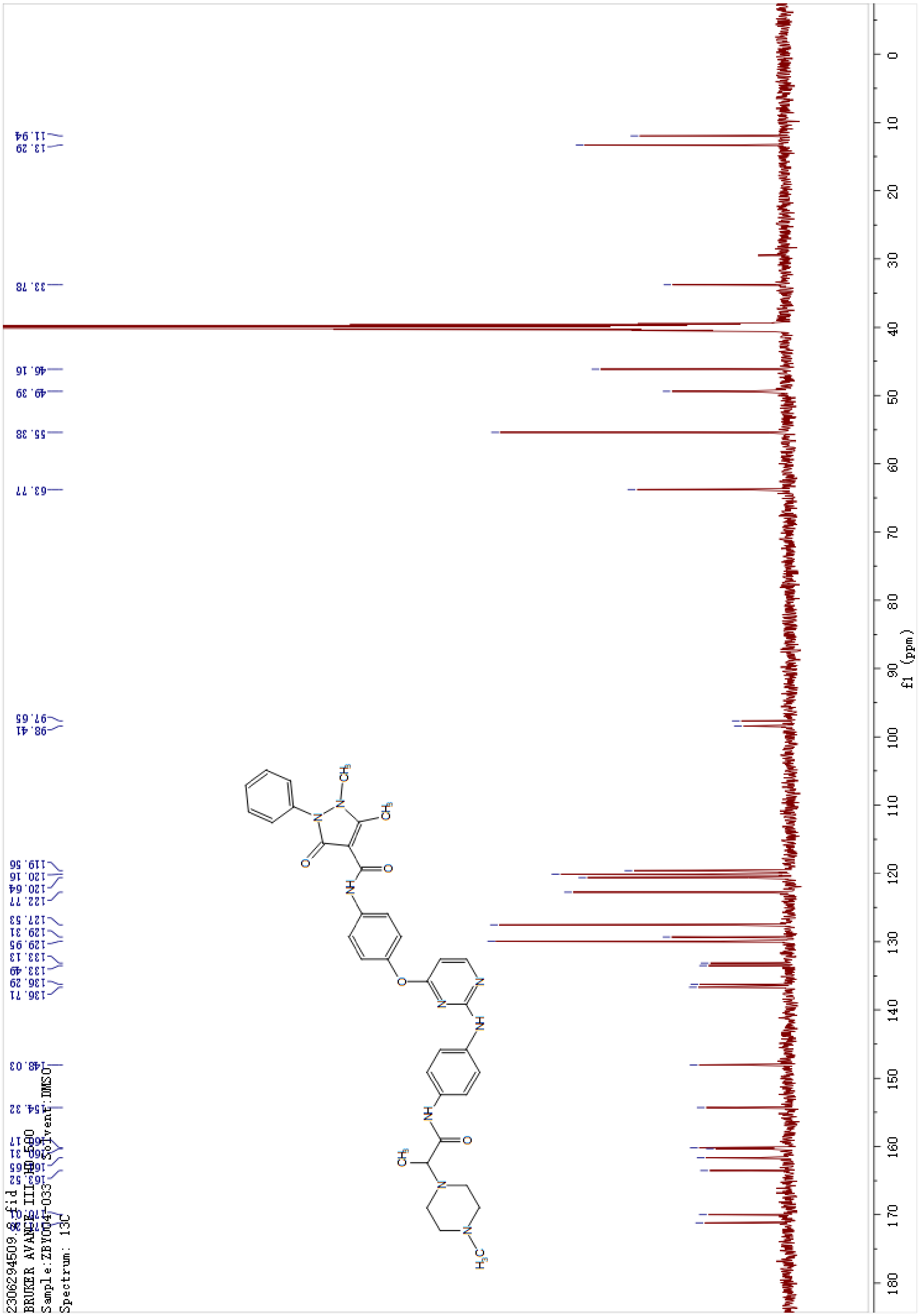

S79: ^{13}C-NMR of **17h**

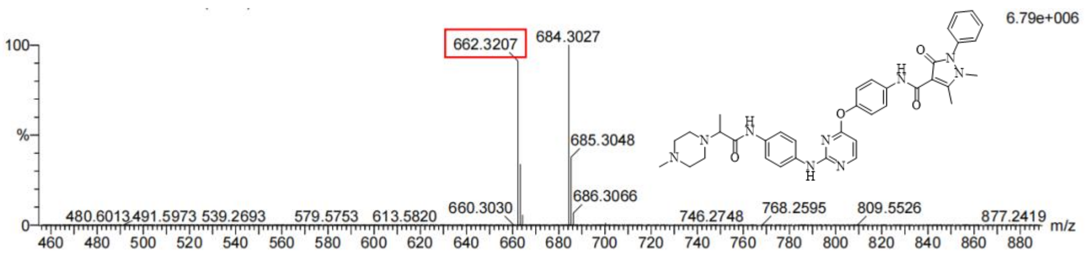

S80: HRMS of **17h**

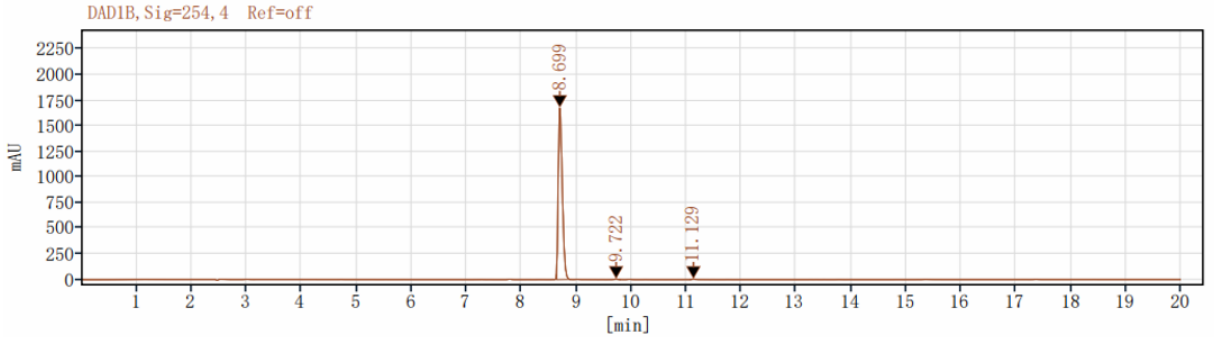

	Retention time (Min)	Peak width (Min)	Area (μV*s)	Height (μV)	% Area
1	8.699	0.26	8525.47	1679.56	99.59
2	9.722	0.13	18.12	4.99	0.21
3	11.129	0.11	16.87	4.78	0.20

S81: HPLC of **17h**

S_{82}: A quantification, a fold change analysis should be desirable

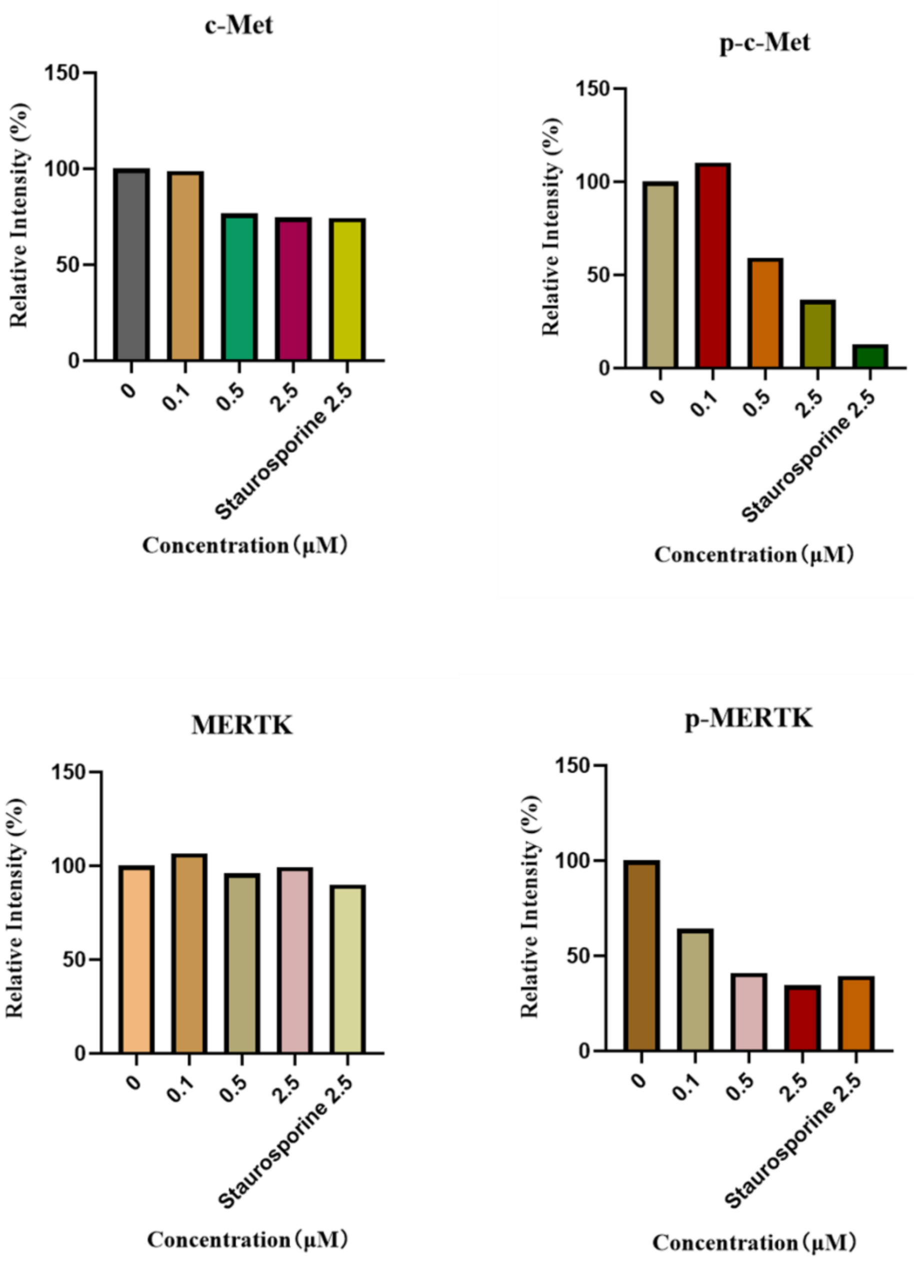

S83: Re-docking Files

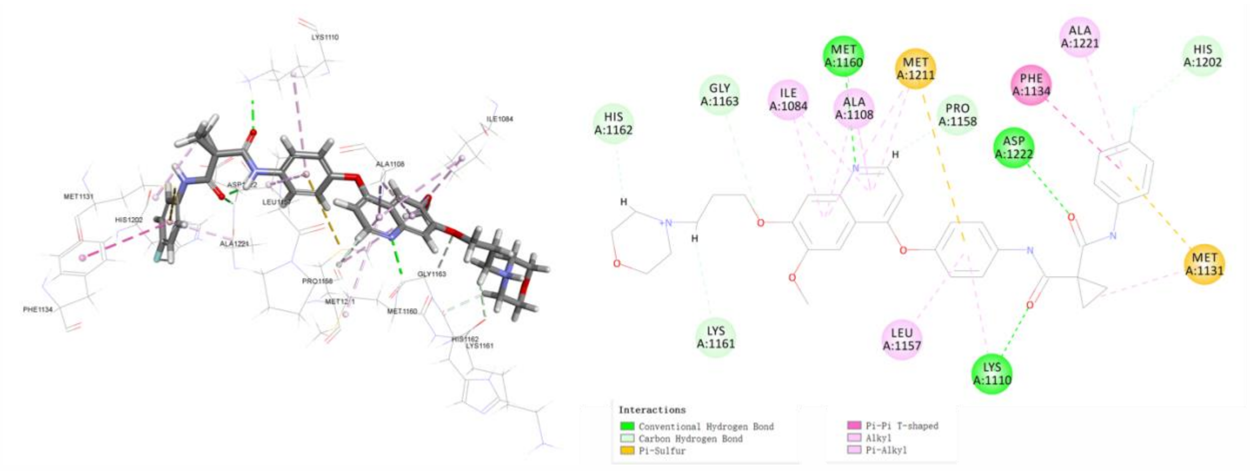

Foretinib with c-Met (PDB: 3LQ8)

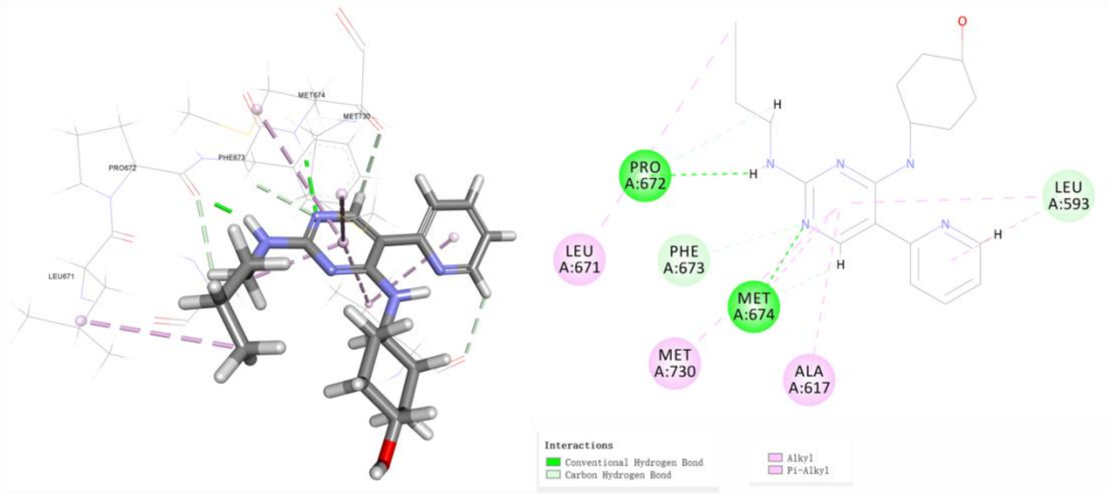

UNC2371 with Mer (PDB: 4M3Q)

S84: Alignment of 17c with foretinib

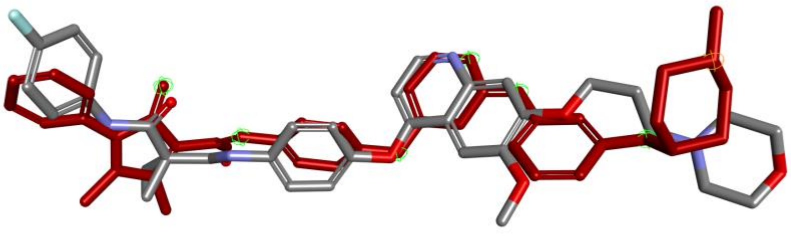

Red: 17c; Gray: foretinib

S85: Original Western blot images

(1) Original Image for Figure 10--c-Met HCT116

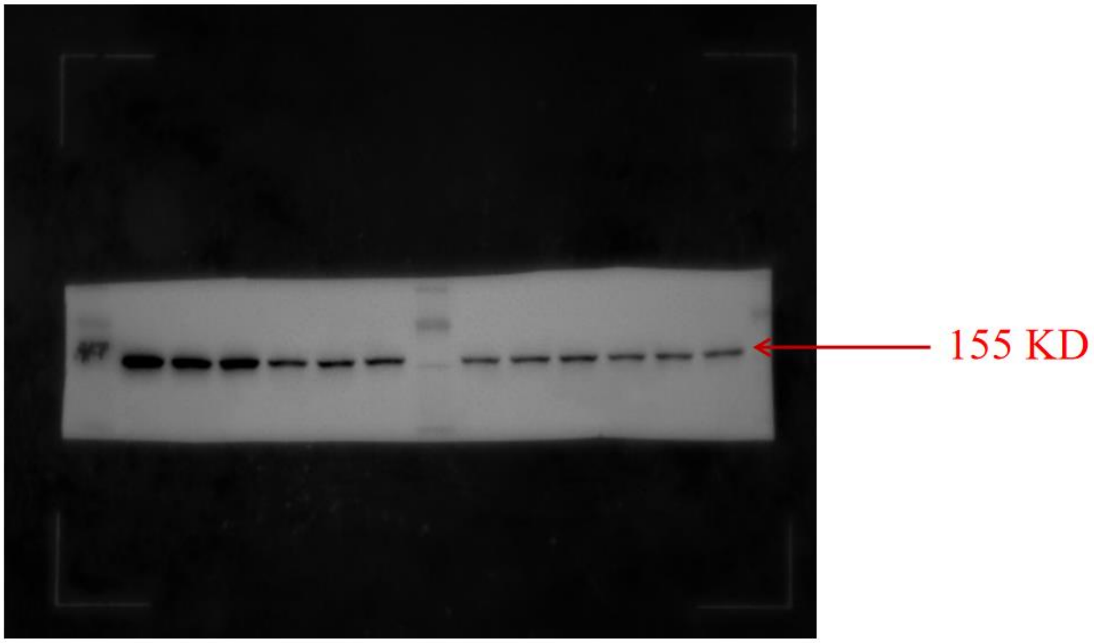

(2) Original Image for Figure 10-MerTK HCT116

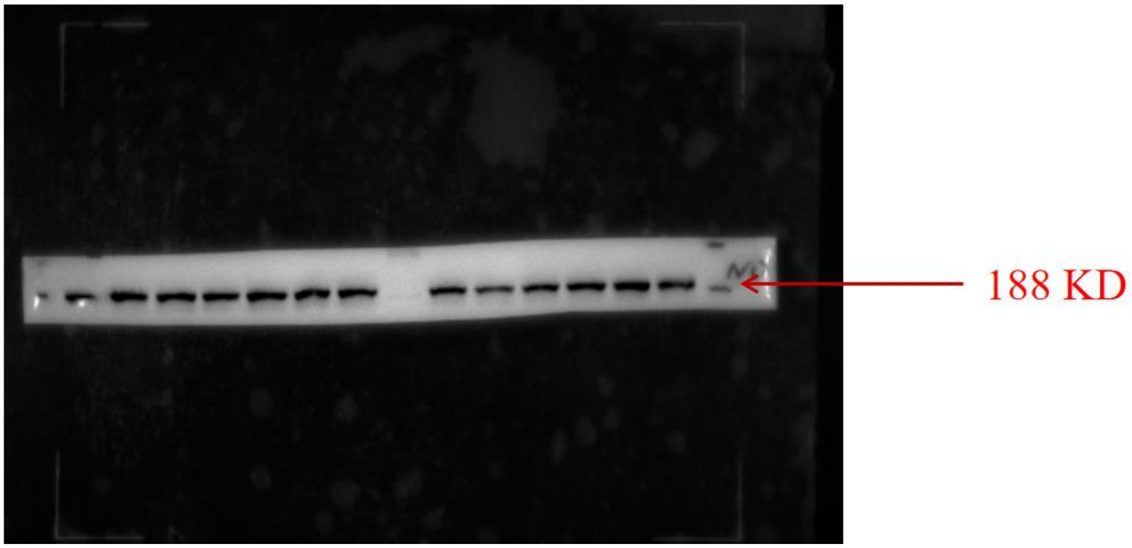

(3) Original Image for Figure 10-p-c-Met HCT116

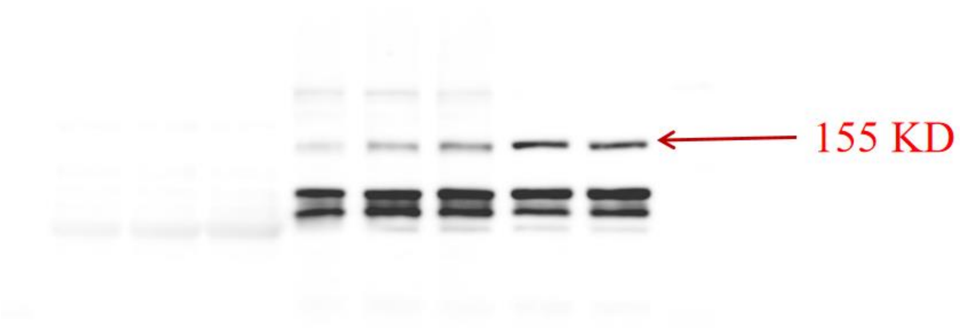

(4) Original Image for Figure 10-p-MerTK HCT116

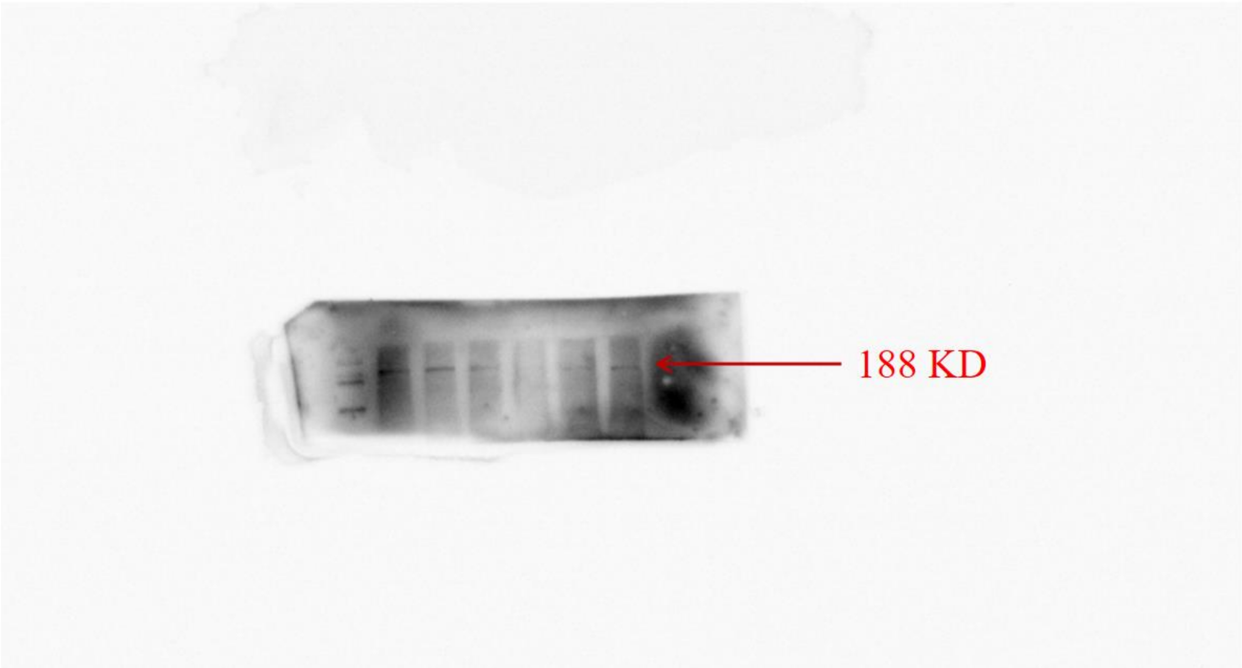

Supplement: Supplementary file 1 [file biomolecules-15-01180-s001.zip › biomolecules-3654458-Supplementary.pdf]
